# Efficient Production of Reactive Oxidants by Atmospheric Bacterial-Derived Organic Matter in the Aqueous Phase

**DOI:** 10.1021/acs.est.5c01526

**Published:** 2025-03-28

**Authors:** Yushuo Liu, Yitao Li, Wing Lam Chan, Yingyu Bao, Patrick K. H. Lee, Theodora Nah

**Affiliations:** † School of Energy and Environment, 53025 City University of Hong Kong, Kowloon, Hong Kong SAR 999077, China; ‡ Shenzhen Research Institute, City University of Hong Kong, Shenzhen 518057, China; § State Key Laboratory of Marine Pollution, City University of Hong Kong, Kowloon Hong Kong SAR 999077, China

**Keywords:** bacterial-derived organic matter, extracellular polymeric substances, singlet oxygen, hydroxyl radical, organic triplet excited states, atmospheric photochemistry

## Abstract

Hydroxyl radicals (•OH), singlet oxygen (^1^O_2_*), and organic triplet excited states (^3^C*) play key roles as oxidants (“reactive intermediates (RIs)”) in forming and oxidizing aqueous organic aerosols. Bioaerosols are ubiquitous in the atmosphere, but little is known about their photochemical behavior and contributions to atmospheric photochemistry. We investigated the photochemical behavior of aqueous-phase cellular organic matter (COM) and extracellular polymeric substances (EPS) from cultured bacteria isolated from atmospheric PM_2.5_, focusing on their photochemical production of ^3^C*, ^1^O_2_*, and •OH. The molecular size and aromaticity of chromophores and fluorophores in COM and EPS increased with molecular weight (MW). Apparent quantum yields (Φ_RI_) of up to 10% and 5% were measured for ^1^O_2_* and ^3^C*, respectively, which are in the upper range of previously reported values. This indicated that COM and EPS contain photosensitizers that are highly efficient at producing ^1^O_2_* and ^3^C*. Φ_RI_ and concentrations ([RI]_ss_) decreased with MW due to higher-MW molecules engaging in charge-transfer interactions that disrupt photochemical processes and oxidant production. Machine learning models were used to understand and predict oxidant production based on measurable optical and chemical properties of COM and EPS. This study provides new insights into the roles that bioaerosols can play in atmospheric aqueous photochemistry.

## Graphical abstract

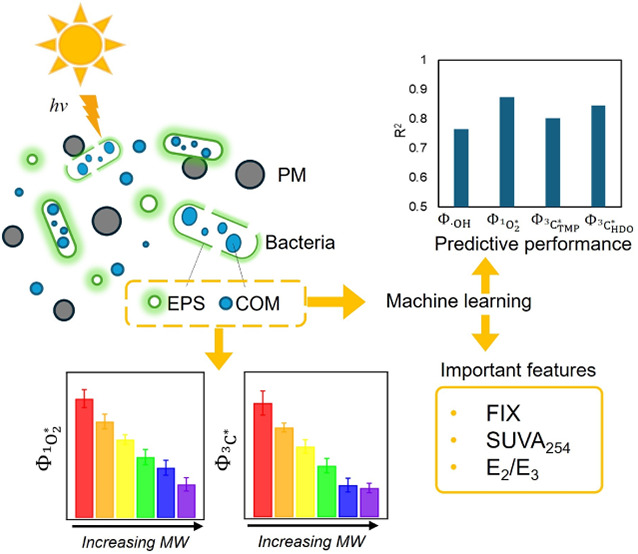

## 1 Introduction

Photochemical reactions drive many major transformation pathways of organic compounds in atmospheric aqueous phases (e.g., aqueous aerosols, cloud and fog droplets).^1^ Hydroxyl radicals (•OH), singlet oxygen (^1^O_2_*), and organic triplet excited states (^3^C*) are important oxidants (also known as “reactive intermediates (RIs)”) that form and oxidize organic matter in atmospheric aqueous phases. Light-absorbing organic matter, brown carbon (BrC), is a key precursor for the photosensitized formation of aqueous oxidants. Upon absorbing sunlight, some BrC chromophores are promoted from their ground states to form ^3^C*, which can undergo oxidative reactions with organic compounds, as well as form other oxidants, including ^1^O_2_* and •OH.^2^ Although •OH typically has the highest reactivity of the three oxidants, the higher concentrations of ^3^C* and ^1^O_2_* can compensate for their lower reactivities, making them competitive oxidants for some aqueous organic compounds.^3–6^ Biomass burning aerosols, anthropogenic secondary organic aerosols, and cooking organic aerosols have been reported to contain BrC chromophores that serve as photosensitizers that produce oxidants efficiently,^5–8^ with quantum yields as high as 5% and 6% reported for ^3^C* and ^1^O_2_*, respectively.^6,7^ However, there are likely other sources of aerosols containing photosensitizers that are efficient producers of ^3^C*, ^1^O_2_*, and •OH that have yet to be identified. Additionally, it is unknown how the optical and chemical characteristics of aerosols correlate with their ability to serve as photosensitizers to produce ^3^C*, ^1^O_2_*, and •OH. Establishing these relationships would be helpful for predicting oxidant production based on measurable aerosol properties.

It is widely recognized that photochemical processes involving photochemically active organic matter in atmospheric aqueous phases and other condensed phases in contact with the atmosphere (e.g., natural ground, surfaces in the built environment) play important roles in atmospheric photochemistry,^1,9^ but little is known about the photochemical behavior of bioaerosols and their contributions to atmospheric photochemistry. Bioaerosols, which include metabolically active and inactive microbial cells and biological debris (e.g., cell fragments, cell-free biomolecules) originating from biological entities, account for about 25% of atmospheric aerosols larger than 200 nm over terrestrial and oceanic surfaces.^10–14^ Bioaerosols may dominate the total aerosol population in geographically isolated regions.^13–15^ While bioaerosols contribute about 30% of the aerosol load in urban and rural air, their contributions could be as high as 80% in clean rainforest air.^14^ Their involvement in various atmospheric biochemical (e.g., biodegradation of organic compounds in atmospheric aqueous phases) and biophysical (e.g., ice and cloud nucleation) processes has significant implications for atmospheric chemistry and physics, climate, ecosystem, and human health.^12,16–25^ Airborne bacterial cells, which can either be free-floating or attached to aerosols, are subjected to various atmospheric stresses, including exposure to sunlight, aerosol acidity, oxidative stress, and low water activity. Consequently, the functioning of metabolically active bacteria in the atmosphere will vary with atmospheric conditions and over spatial and temporal scales. The presence of thriving, functionally diverse, and metabolically active bacterial communities in clouds (10^4^ to 10^6^ cells/mL) has been attributed to clouds being “microbial oases” that provide dissolved organic nutrients and liquid water needed for bacterial survival and activity.^26^ Metabolically active bacteria in clouds have been found to express genes related to different types of bacterial functions, including energy metabolism, processing of carbon and nitrogen compounds, transmembrane transport, intracellular signaling, and stress response.^27–29^ Many of these bacterial functioning processes can lead to the biodegradation of some organic compounds (e.g., carboxylic acids,^17,30^ amino acids,^31^ phenolic compounds^32^) and the biosynthesis and release of chemically complex cellular organic matter (COM),^33^ all of which lead to modifications in the composition of organic matter in clouds. Dominant atmospheric bacterial taxa have biotic traits to counter atmospheric stresses during transport and deposition, including entering cellular dormancy, sporulation, and secreting metabolites as protection against oxidative stress and low water activity.^12,34–37^ Secreting hygroscopic extracellular polymeric substances (EPS) composed of polysaccharides, proteins, and DNA is commonly employed by atmospheric bacteria to mitigate the effects of low water activity and oxidative stress.^27,34^ Failure to withstand atmospheric stress can result in bacterial cell lyse, which will release biomolecules as large as >50 kDa.^30,38^ Some aqueous biomolecules strongly absorb near-UV and visible light,^38^ but, in general, little is known about the photochemical behavior and ability of microbial-derived organic matter (including bacterial COM and EPS) to produce oxidants in atmospheric aqueous phases. Being able to efficiently produce aqueous oxidants will provide bioaerosols with an avenue to be significant contributors to photochemical reactions in atmospheric aqueous phases and interfaces.

Given the ubiquity and abundance of bioaerosols in the atmosphere, there is a need to investigate the photochemical behavior of microbial-derived organic matter and their ability to produce aqueous oxidants to advance our knowledge of the roles that bioaerosols play in atmospheric aqueous photochemistry. Here, we present the first-ever measurements of the optical and chemical characteristics, photochemical behavior, and ^3^C*, ^1^O_2_*, and •OH production by aqueous extracts of COM and EPS from culturable bacteria isolated from PM_2.5_ samples collected in Hong Kong and pure bacterial cultures. We show that atmospheric COM and EPS contain photosensitizers that are efficient oxidant producers, thus highlighting the ability of bioaerosols to contribute to photochemical reactions in atmospheric aqueous phases and interfaces. Additionally, we relate measurements of the optical and chemical characteristics to the apparent quantum yields and steady-state concentrations of ^3^C*, ^1^O_2_*, and •OH using correlation analysis, and develop multiple linear regression and machine learning models to identify key optical and chemical parameters that govern their production and assess the possibility of predicting their apparent quantum yields and steady-state concentrations based on these parameters.

## 2 Materials and Methods

### 2.1 Sample Collection and Preparation

Section S1 lists the chemicals used in this study. Ultrapure water (Milli-Q, Merck, 18.2 MΩ cm) was used to prepare all solutions. Four pure cultures of bacteria were used: *Bacillus subtilis* ATCC 6051-U and *Pseudomonas putida* ATCC 23467 (both from the American Type Culture Collection), *Enterobacter hormaechei* B0910, and *Enterobacter hormaechei* pf0910 (isolated from an ambient air sample in Hong Kong). *B. subtilis* and *P. putida* are common in outdoor environments. The bacterial strains were grown in either Nutrient Broth (NB) (*P. putida* ATCC 23467) or Luria–Bertani (LB) broth (*B. subtilis* ATCC 6051-U, *E. hormaechei* B0910, and *E. hormaechei* pf0910) at 30 °C to the stationary phase. The cultures were then centrifuged at 6000 rpm for 10 min at 4 °C before the cell pellets were extracted.

Culturable bacteria from PM_2.5_ collected in Hong Kong during the summer (3 days) and winter (6 days) were also used in this study (Table S1). The PM_2.5_ samples were collected on prebaked (550 °C for 12 h) 8″ × 10″ quartz filters (Tissuquartz, 2500 QAT-UP, Pall Corporation, USA) using a high-volume sampler (TE-6070-2.5-HVS, Tisch Environmental, USA) located at the ground level of a roadside site (22°20′05″N, 114°10′23′′E) with medium traffic volume, situated in an urbanized area surrounded by residential and commercial facilities. For each sampling day, PM_2.5_ samples were collected for 24 h at a flow rate of 85 m^3^/h. Blank samples were generated in the same way as the PM_2.5_ samples, except the air pump was switched off during sampling. Immediately after collection, the filters were transferred into individual sterilized centrifuge tubes, to which 20 mL of ultrapure water was added. The tubes were then vortexed for homogenization. 2 mL of the resulting homogenate was incubated in 10 mL of culture medium at 30 °C and shaken at 200 rpm until the bacteria were grown to the stationary phase. COM and EPS were biosynthesized by the bacteria during their growth process. Four different media were used to culture the bacteria in each homogenate: LB, NB, Tryptic Soy Broth (TSB), and Yeast Extract Peptone Dextrose (YPD). The cultured bacteria were used either for DNA extraction and amplicon sequencing of the 16S rRNA gene (Section S2) or stored at −80 °C until further analysis for COM and EPS. The amplicon sequencing data was used to determine the abundances and diversities of the culturable bacterial communities in PM_2.5_ (Figure S1). There was no bacterial growth from the blank filters. Each stored bacterial sample was grown to the stationary phase using the same culture medium and method described above. Equal volumes of cultured bacteria obtained from different days within the same season and grown in the same medium were combined. The resulting culture was then centrifuged at 6000 rpm for 10 min at 4 °C, and the cell pellets were rinsed with ultrapure water three times.

The cell pellets from the pure cultures and PM_2.5_ samples were used to extract COM and EPS via ultrasonic and modified heating methods, respectively (Section S3). All the extracted COM and EPS samples were filtered using 0.22 μm pore size PTFE syringe filters (Tianjin Jinteng Experiment Equipment Co. Ltd., China). The extracted bulk COM and EPS samples are denoted as C0 and E0, respectively. To obtain molecular weight (MW)-fractionated COM and EPS samples, the filtered COM and EPS extracts were ultrafiltered using five centrifugal size filters (Pall Corporation, USA): 1 kDa, 3 kDa, 10 kDa, 30 kDa, and 50 kDa. This resulted in six MW-fractionated samples each for COM (C1 to C6) and EPS (E1 to E6): C1 and E1 (<1 kDa), C2 and E2 (1–3 kDa), C3 and E3 (3–10 kDa), C4 and E4 (10–30 kDa), C5 and E5 (30–50 kDa), and C6 and E6 (>50 kDa).

### 2.2 Characterizations of the Bulk and MW-Fractionated COM and EPS Samples

The dissolved organic carbon (DOC) concentrations of all the bulk and MW-fractionated COM and EPS samples were measured using a TOC analyzer (TOC-L CPH, Shimadzu, Japan). The concentration of each sample was then diluted to 5 mg of C/L with ultrapure water for subsequent analysis and photochemistry experiments. Proteins and polysaccharides in each sample were quantified using procedures described in Section S3. UV–visible absorption spectra (200–800 nm with 1 nm increments) were measured by a UV–visible absorption spectrophotometer (UV-33600, Shimadzu Corp., Japan). Excitation–emission matrix (EEM) fluorescence spectra (excitation wavelength: 200–500 nm with 10 nm increments, emission wavelength: 250–600 nm with 5 nm increments) were measured with a spectrofluorometer (FluoroMax-4, Horiba, Japan). Parallel factor (PARAFAC) analysis was performed on the EEM fluorescence spectra to determine the composition of the fluorophores in the COM and EPS samples. Details of the EEM fluorescence measurements and PARAFAC analysis can be found in Section S4, Figure S2, and Table S2. Six components were identified based on comparisons of their extracted spectra to those reported in previous studies:^39–42^ flavin-like, tryptophan-like, tyrosine-like, HULIS-1, fulvic-like, and HULIS-2.

### 2.3 Photochemistry Experiments

The COM (pH ∼ 6.3) and EPS (pH ∼ 7.6) solutions were adjusted to pH 7 using a 10 mM phosphate buffer (Shanghai Macklin Biochemical Co., Ltd.) before the experiments. Quartz tubes containing 10 mL of solutions of COM/EPS spiked with a chemical probe were placed onto a rotating vial rack and irradiated with a 300 W xenon lamp (GZYY300W, Yuye Co., China) equipped with a long-pass filter of 3.3 mm thickness and a wavelength cutoff at 300 nm (ZJB300, Taizhu Co., China). The photon flux under our experimental conditions is shown in Figure S3. The solution temperatures (25 °C) were regulated by a fan directed at the tubes.

The methods used to measure •OH, ^1^O_2_*, and ^3^C* in this study have been described previously.^43^ Benzene (1–3 mM) was used as the chemical probe for •OH, and the formation of phenol was measured during the experiments.^44^ Furfuryl alcohol (FFA) (10 μM) was used as the chemical probe for ^1^O_2_*, with isopropanol added into the solutions to act as the •OH quencher.^45,46^ The decay of FFA was measured during the experiments. ^3^C* is composed of a mixture of organic triplet excited states. Due to the chemical complexity and diversity of ^3^C* precursors, it is difficult to characterize ^3^C* using a single chemical probe. Thus, 2,4,6-trimethylphenol (TMP) (10 μM) and *trans*,*trans*-2,4-hexadien-1-ol (*t*,*t*-HDO) (0.025 to 0.5 mM) were used as chemical probes for electron-transferred ^3^C* (^3^C*_TMP_) and energy-transferred ^3^C* (^3^C*_HDO_), respectively.^47,48^ Only subsets of ^3^C* that react with TMP and *t*,*t*-HDO were quantified in our study. The decay of TMP was measured during the experiments, and the degradation of TMP due to reactions with •OH and ^1^O_2_* was accounted for in calculations. Photoisomerization and the formation of *c*,*c*-HDO were measured during the experiments using *t*,*t*-HDO as the chemical probe. Electron-transferred ^3^C* are considered to be oxidizing ^3^C* since they oxidize organic compounds via single-electron transfer and proton-coupled electron transfer reactions, whereas energy-transferred ^3^C* refers to the larger pool of ^3^C*.^49^ Calculations of the apparent quantum yields (Φ_RI_), and steady-state concentrations ([RI]_ss_) of the oxidants (RI: •OH, ^1^O_2_*, and ^3^C*) are detailed in Section S5. The chemical probes or their products were detected by ultrahigh-performance liquid chromatography coupled to a photodiode array detector (UPLC-PDA, Acquity H-Class, Waters) (Table S3, Figures S4–S6). A separate set of photochemistry experiments was also performed without the addition of chemical probes. Changes in absorbance and fluorescence were measured during these experiments. All measurements and experiments were performed in triplicate.

### 2.4 Identification of Important Features and Predictors

Correlations between measurements for COM and EPS from culturable bacteria in PM_2.5_ were quantified using Spearman’s rank correlation analysis in Origin. Student’s *t*-tests to test for significance were conducted by using Prism 8. Multiple linear regression (MLR) models for Φ_RI_ and [RI]_ss_ were developed in SIMCA 14.1 and Prism 8 by using measured optical and chemical parameters (Section S6). Additionally, five commonly used candidate tree-based machine learning (ML) algorithms (random forest (RF), gradient boosting decision tree (GBDT), light gradient boosting machine (LGB), extreme gradient boost (XGB), and categorical boosting (CB))^50^ in Python 3.11 were used to investigate links between features of the optical and chemical parameters and Φ_RI_ and [RI]_ss_ and to assess the possibility of making Φ_RI_ and [RI]_ss_ predictions based on these parameters (Section S7). The ML algorithms were modified based on scikit-learn (v.1.1.3) and previous studies.^51–53^ 80% of the data was used for training, while the remaining 20% was used for testing. Since the CB algorithm had substantially higher cross-validation scores than the other ML algorithms for ln­(Φ_RI_) and ln­([RI]_ss_) (Table S4), it was used to identify important features and predictors for ln­(Φ_RI_) and ln­([RI]_ss_). The hyperparameters of the ln­(Φ_RI_) and ln­([RI]_ss_) models were subsequently tuned using Bayesian optimization to enhance their predictive performance on the training sets.^54,55^ Table S5 summarizes the predictive scores (coefficient of determination (*R* ^2^) and root-mean-square error (RMSE)) for the ln­(Φ_RI_) and ln­([RI]_ss_) models on the training, test, and cross-validation sets. Details of the tree-based feature importance analysis, Shapley’s additive interpretation (SHAP), and partial dependence plots (PDP) used to interpret the results of the optimized models can be found in Section S7.

## 3 Results and Discussion

### 3.1 Characteristics of COM and EPS

The bulk and MW-fractionated COM and EPS samples from culturable bacteria in PM_2.5_ and pure cultures exhibited similar trends (Figures 1, S7–S10, Tables S6–S9). The bulk and MW-fractionated EPS samples had higher and lower concentrations of polysaccharides and proteins, respectively, than their corresponding COM samples. For both COM and EPS, the polysaccharide and protein concentrations decreased and increased with molecular weight, respectively.

**Figure 1:**
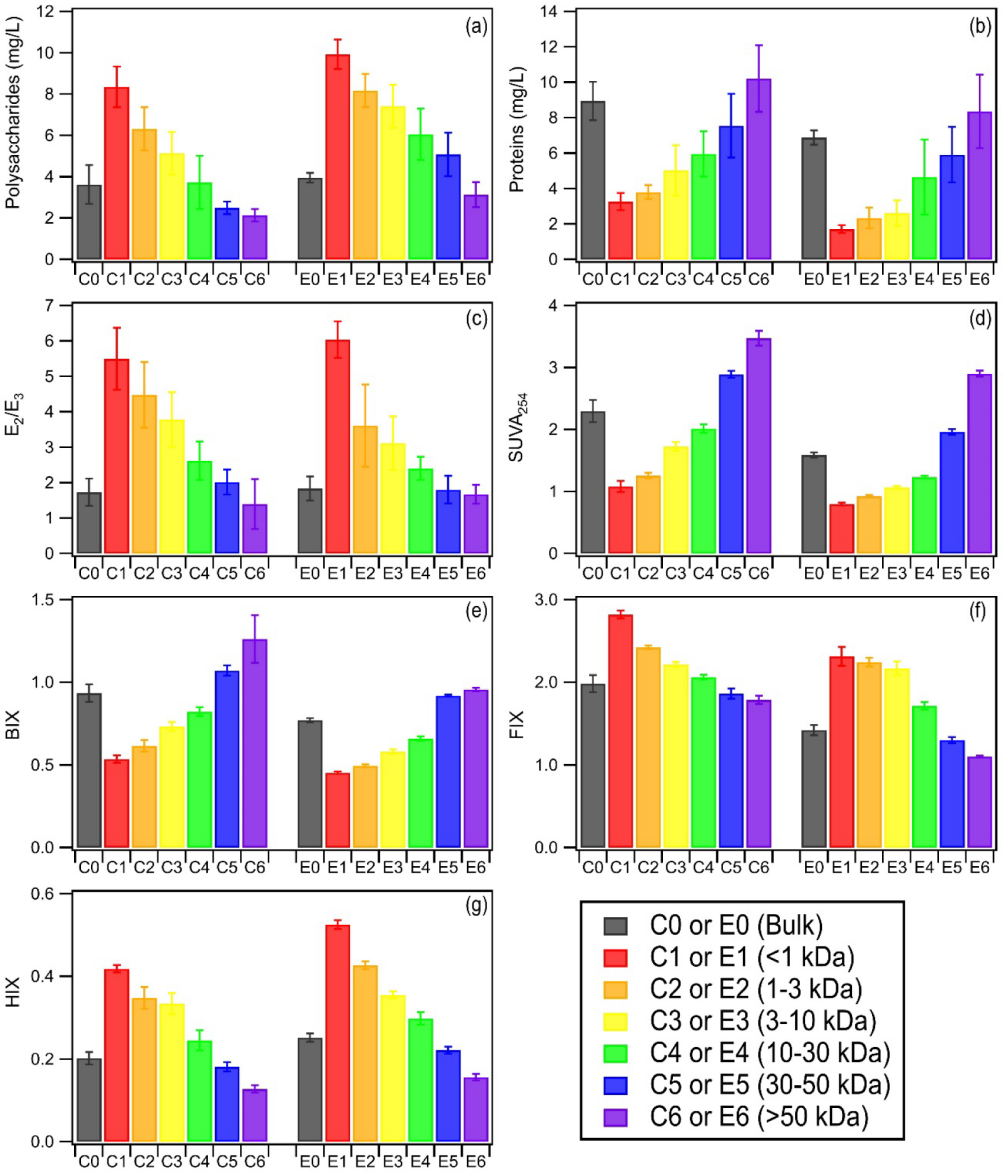
(a) Polysaccharides and (b) protein concentrations, (c) *E*_2_/*E*_3_, (d) SUVA_254_, (e) BIX, (f) FIX, and (g) HIX of bulk and MW-fractionated COM and EPS samples extracted from culturable bacteria in PM_2.5_. Shown here are the averages for the summer and winter data. Error bars denote one standard deviation. The DOC of each fraction was fixed at 5 mg C/L.

Optical indices *E*_2_/*E*_3_, SUVA_254_, BIX, HIX, and FIX (Table S10) were derived from absorption and fluorescence measurements. The *E*_2_/*E*_3_ ratio, which denotes the size of chromophores,^56^ decreased with MW for COM and EPS, indicating that the molecular size of chromophores increased with MW. SUVA_254_, which has a direct relationship with the aromaticity of chromophores,^57^ increased with MW for COM and EPS, indicating that the higher MW fractions exhibited higher aromaticity. Similar observations have been reported for dissolved bacterial-derived organic matter in aquatic systems.^58,59^ The fluorescence index (FIX) has an inverse relationship with the aromaticity of fluorophores, with a smaller FIX value indicating a higher degree of aromaticity.^60,61^ FIX decreased with MW for COM and EPS, indicating that the higher MW fractions exhibited higher aromaticity, which is consistent with their SUVA_254_ measurements. The bulk and MW-fractionated COM samples had SUVA_254_ and FIX values higher than those of their corresponding EPS samples. This suggested that while the chromophores in the COM samples had higher aromaticity than the EPS samples, the fluorophores in the COM samples had lower aromaticity than the EPS samples. Fluorophores comprise a subset of chromophores in water-soluble organic matter,^62^ but the size of this subset is currently unknown. The humification (HIX) and biological (BIX) indices are typically used to determine the humification of fluorphores and microbial-derived contribution to fluorophores.^60^ HIX decreased with MW for COM and EPS, indicating that the higher MW fractions exhibited lower humification, which is consistent with the lower levels of humic-like components found in the higher MW fractions. BIX increased with MW for COM and EPS, which is consistent with the higher levels of tyrosine-like and tryptophan-like components (i.e., F2 and F3) found in the higher MW fractions. The bulk and MW-fractionated COM samples had higher BIX and lower HIX values than their corresponding EPS samples, which is consistent with the PARAFAC results.

There was little difference in the protein and polysaccharide concentrations, absorption, and fluorescence measurements for the COM and EPS extracted from culturable bacteria in summer vs winter PM_2.5_ and extracted using different culture media (Figure S11). This could be due to the dominance of the *Bacillus* genus in both summer and winter PM_2.5_ (Figure S1), which resulted in similar COM and EPS production. Previous studies have similarly reported the predominance of the *Bacillus* genus in cultivable bacteria in PM_2.5_ in other locations.^63–65^

### 3.2 Photochemical Behavior of COM and EPS

Six components were extracted from the EEM fluorescence spectra of COM and EPS by PARAFAC analysis (Table S2). Protein-like components F1, F2, and F3 were denoted as flavin-like, tyrosine-like, and tryptophan-like components, respectively.^42,66^ Components F2 and F3 for the higher MW fractions were likely contributed by proteins and/or protein-like compounds, whereas free amino acids, peptides, and aliphatic amines likely contributed to components F2 and F3 for the lower MW fractions.^67^ Component F1 likely originated from riboflavin-like compounds.^68,69^ The contributions of components F2 and F3 to the overall fluorescence level increased with MW (Figures 2 and S12), which is consistent with previous studies on bacterial-derived organic matter in atmospheric and aquatic systems.^38,58,70^ The opposite trend was observed for component F1. Humic-like components F4, F5, and F6 were denoted as HULIS-1 (highly oxygenated species), fulvic-like, and HULIS-2 (less oxygenated species) components, respectively.^40,71^ Components F4 and F5 are considered to be substantially more oxygenated than component F6, which has a higher aliphatic content.^40,42,71^ The bulk and MW-fractionated EPS samples had higher levels of humic-like components and lower levels of protein-like components than their corresponding COM samples. Nevertheless, it is important to note that these terms (tryptophan-like, tyrosine-like, etc.) are generalized terms corresponding to highly complex groups of unresolved organic compounds that are categorized by their fluorescence spectra. For instance, non-nitrogen-containing compounds with fluorescence similar to these protein/protein-like compounds (e.g., phenolic compounds) can still contribute to the fluorescence levels of components F1, F2, and F3.^40,42,72^

**Figure 2:**
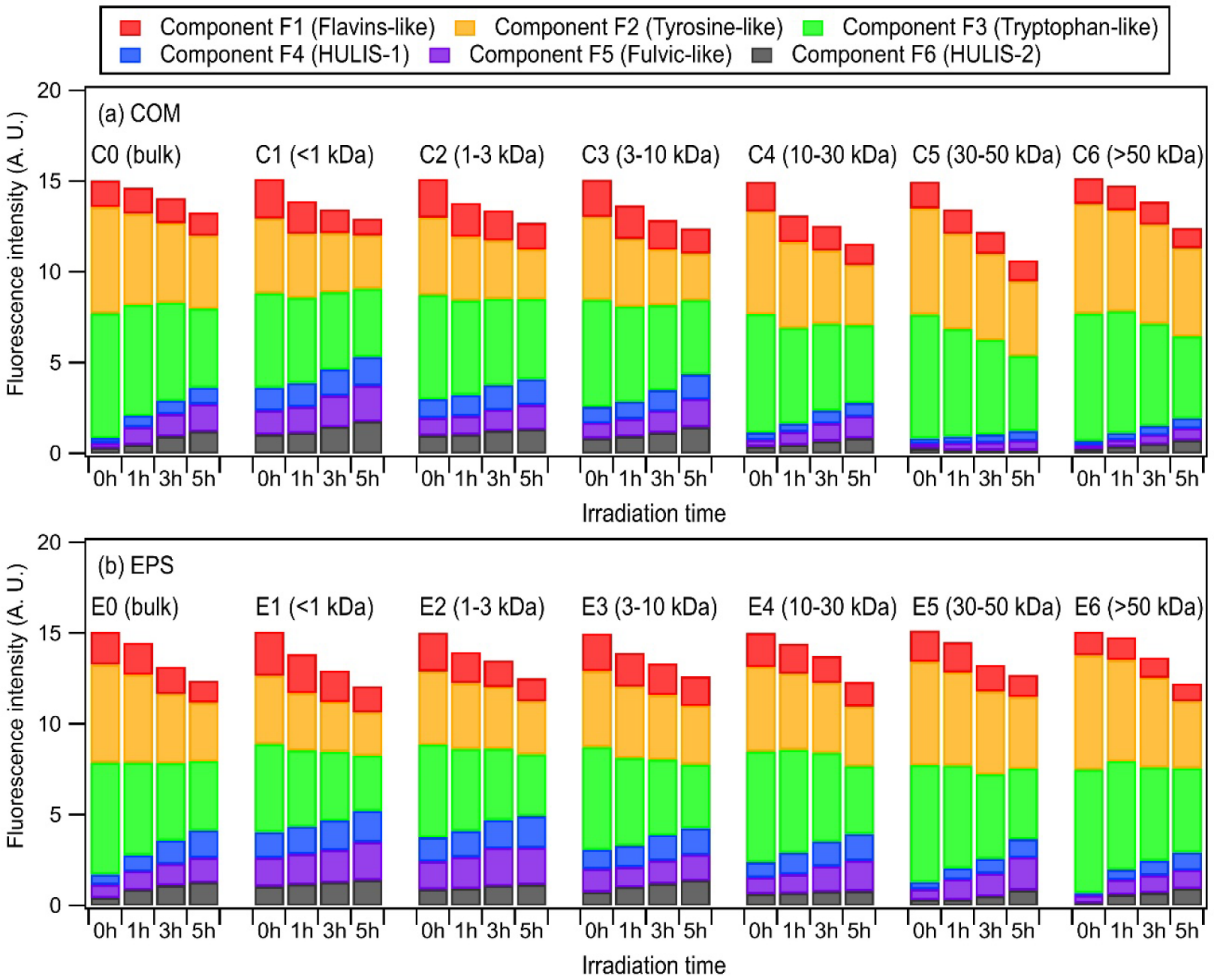
Time evolution of the six PARAFAC-extracted components for bulk and MW-fractionated (a) COM and (b) EPS samples extracted from culturable bacteria in PM_2.5_ during irradiation. The DOC of each fraction was fixed at 5 mg C/L before irradiation.

The overall absorbance and fluorescence for all of the COM and EPS samples extracted from culturable bacteria in PM_2.5_ and pure cultures decreased with irradiation in the absence of chemical probes (Figures 2, S12–S25). Similar observations have been reported for the irradiation of the water-soluble fraction of PM_2.5_.^73^ Components F1, F2, and F3 exhibited significant decreases in their levels across all of the COM and EPS samples, indicating their photodegradations. In contrast, the levels of components F4, F5, and F6 increased with irradiation. This indicated that the photodegradations of flavin-like and protein/protein-like compounds led to the photochemical formation of HULIS and fulvic-like compounds. Significant increases (*p* < 0.05, Student’s *t*-test) in the levels of the highly oxygenated components F4 and F5 from 0 to 5 h could be attributed to the formation of phenolic-like compounds from photochemical reactions.^74,75^ Increases in the levels of the less oxygenated component F6 from 0 to 5 h were statistically insignificant (*p* > 0.05, Student’s *t*-test). This could be due to the conversion of less oxygenated humic-like components to more oxygenated humic-like components, leading to small increases in component F6.

### 3.3 Production of •OH, ^1^O_2_*, and ^3^C* from COM and EPS

Figure 1 highlights the optical properties of chromophores in COM and EPS biosynthesized by culturable bacteria in PM_2.5_. Most notably, the MW-dependent trends of SUVA_254_ indicated that the higher MW fractions had higher aromaticity. Aromatic structures are known to be important chromophoric moieties that serve as ^3^C* photosensitizers.^76^ This suggested that the chromophores in COM and EPS have the ability to form ^3^C* and other oxidants via photosensitization. Chromophoric nonaromatic compounds such as imidazoles, pyrazines, quinones, and chromones can serve as ^3^C* photosensitizers as well.^2,76–78^ Liang et al. (2024) previously provided a comprehensive review of the processes governing atmospheric photosensitization.^76^ Briefly, upon absorption of a photon, the ground-state chromophore is excited to form the organic singlet excited state (^1^C*). In addition to nonradiative and radiative decays via internal conversion and fluorescence, respectively, ^1^C* can be converted to ^3^C* via intersystem crossing. Compared to the very short-lived ^1^C* (estimated lifetime of 150 ps to 6 ns),^79^ ^3^C* has a longer lifetime (estimated lifetime of 10–30 μs),^80^ allowing it to participate in bimolecular reactions before deactivation to the ground state. The chemically complex ^3^C* pool has a wide range of reactivities, with higher- and lower-energy ^3^C* species capable of undergoing energy transfer and electron transfer reactions, respectively. ^3^C* can be deactivated to the ground state by O_2_-independent and O_2_-dependent pathways. Under air-saturated conditions, energy transfer from ^3^C* to O_2_ to form ^1^O_2_* supposedly dominates since the energy required to promote ground-state O_2_ to ^1^O_2_* (94 kJ/mol) is substantially smaller than the energies of most ^3^C* species (180–320 kJ/mol).^2^ •OH is formed through electron transfer reactions,^81,82^ though the exact pathways that lead to •OH formation are not as well understood as those that lead to ^1^O_2_* formation.

Different chemical probes were used to investigate the production of •OH, ^1^O_2_*, electron-transferred (aka oxidizing), and energy-transferred ^3^C* during irradiation of the bulk and MW-fractionated COM and EPS samples extracted from culturable bacteria in PM_2.5_ (Figures 3 and Tables S11–S14). Seasonal variations in oxidant production were not significant (Figure S11). Although the [RI]_ss_ values depend on the initial COM/EPS concentration (set to 5 mg C/L here), it is worth noting that the abundance of [RI]_ss_ for the three oxidants followed the same general order (i.e., •OH < oxidizing ^3^C* < ^1^O_2_*) as those previously reported for PM.^4,7^ Interestingly, while (ΦCTMP*3) values were larger than ΦCHDO*3 values, [CTMP*3]ss values were smaller than [CHDO*3]ss values. This could be due to the larger pool of energy-transferred ^3^C* compared to that of oxidizing ^3^C* produced by COM and EPS.

**Figure 3:**
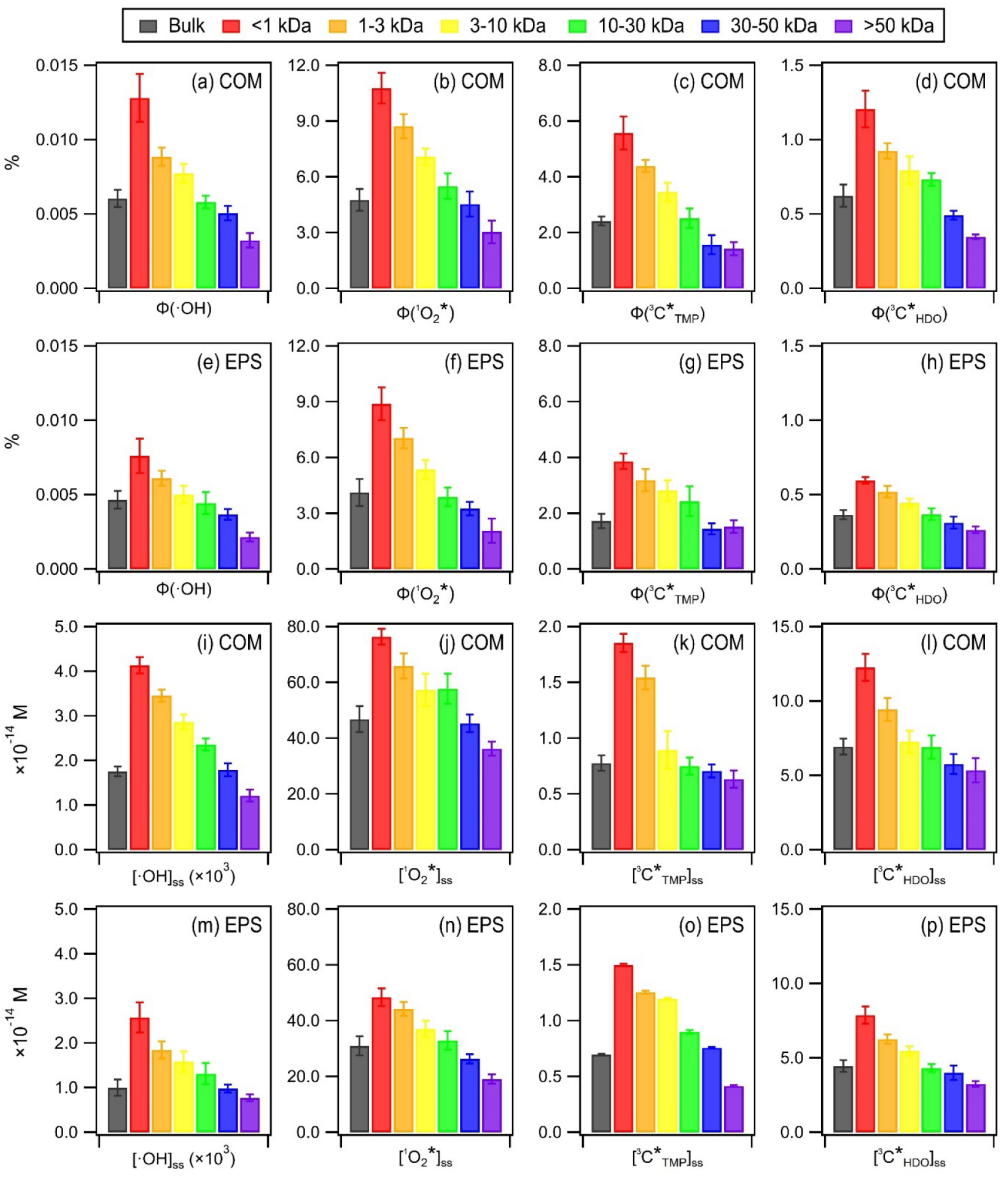
(a–h) Φ_RI_ and, (i–p) [RI]_ss_ of •OH, ^1^O_2_*, and ^3^C* for bulk and MW-fractionated COM and EPS samples extracted from culturable bacteria in PM_2.5_. Shown here are the averages of the summer and winter data. Error bars denote one standard deviation. The DOC of each fraction was fixed to 5 mg C/L before irradiation.

We focused on Φ_RI_, which describes the efficiency of oxidant production and is defined as the number of moles of oxidants produced divided by the number of moles of photons absorbed by the photosensitizer.^46^ The average ΦO2*1 for the bulk COM and EPS samples was (4.77 ± 0.58)% and (4.12 ± 0.73)%, respectively, which are in the upper range of those previously reported for PM (0.3 to 8.4%).^3–5,7,73,83^ Remarkably, the average ΦO2*1 for the MW-fractionated COM and EPS samples was as high as (10.78 ± 0.82)% and (8.89 ± 0.88)%, respectively. The average ΦCTMP*3 for the bulk COM and EPS samples ((2.41 ± 0.15)% and (1.73 ± 0.26)%, respectively) was within the range of Φ_RI_ for oxidizing ^3^C* previously reported for PM (0.3 to 8.8%).^3,4,7^ This indicated that atmospheric bacterial-derived organic matter (in this case, COM and EPS) contains photosensitizers that are efficient at producing ^1^O_2_* and oxidizing ^3^C*, thus giving them an avenue to contribute to photochemical reactions in atmospheric aqueous phases and interfaces.

The Φ_RI_ and [RI]_ss_ for the MW-fractionated COM and EPS samples extracted from culturable bacteria in PM_2.5_ and pure bacteria cultures had similar MW-dependent trends (Figures 3, S26–S29, Tables S11–S14). The highest Φ_RI_ and [RI]_ss_ values were obtained for the lowest MW fractions of COM and EPS. Φ_RI_ and [RI]_ss_ decreased with MW for COM and EPS. Similar observations have been reported for aquatic dissolved organic matter and peat humic substances.^58,79,84–86^ The MW-dependent trends for Φ_RI_ and [RI]_ss_ could be due to higher MW molecules being more likely to engage in charge-transfer (CT) interactions, since these molecules could have multiple charge-transfer contacts within them. CT interactions could result in electron quenching, which would disrupt photochemical processes and oxidant production.^79,87,88^ The bulk and MW-fractionated COM samples had higher Φ_RI_ and [RI]_ss_ than their corresponding EPS samples, indicating that COM are more efficient at producing •OH, ^1^O_2_*, and ^3^C* than EPS. Reasons for the differences in the oxidant production efficiencies for COM vs EPS are beyond the scope of this study.

Results from Spearman’s rank correlation analysis showed that the Φ_RI_ and [RI]_ss_ values had significant positive correlations with each other (*p* < 0.05) for both COM and EPS samples extracted from culturable bacteria in PM_2.5_ (Figures 4 and S30), indicating that some photosensitizers that produced ^3^C* were also precursors for •OH and ^1^O_2_* production. Φ_RI_ was positively correlated with *E*_2_/*E*_3_ and FIX (*p* < 0.05) but negatively correlated with SUVA_254_ (*p* < 0.05), indicating the key roles that the degree of aromaticity and molecular size play in influencing the abilities of photosensitizers to produce oxidants. The correlations between fluorescence components were significant (*p* < 0.05). Components F2 and F3 were positively correlated with each other but negatively correlated with components F1, F4, F5, and F6. Higher levels of components F2 and F3 combined with the lower levels of components F4, F5, and F6 were associated with higher BIX and lower HIX values. In contrast to components F2 and F3, component F1 was positively correlated with Φ_RI_ values (*p* < 0.05). This suggested that small flavin-like compounds of low aromaticity could be key photosensitizers that produce oxidants.

**Figure 4:**
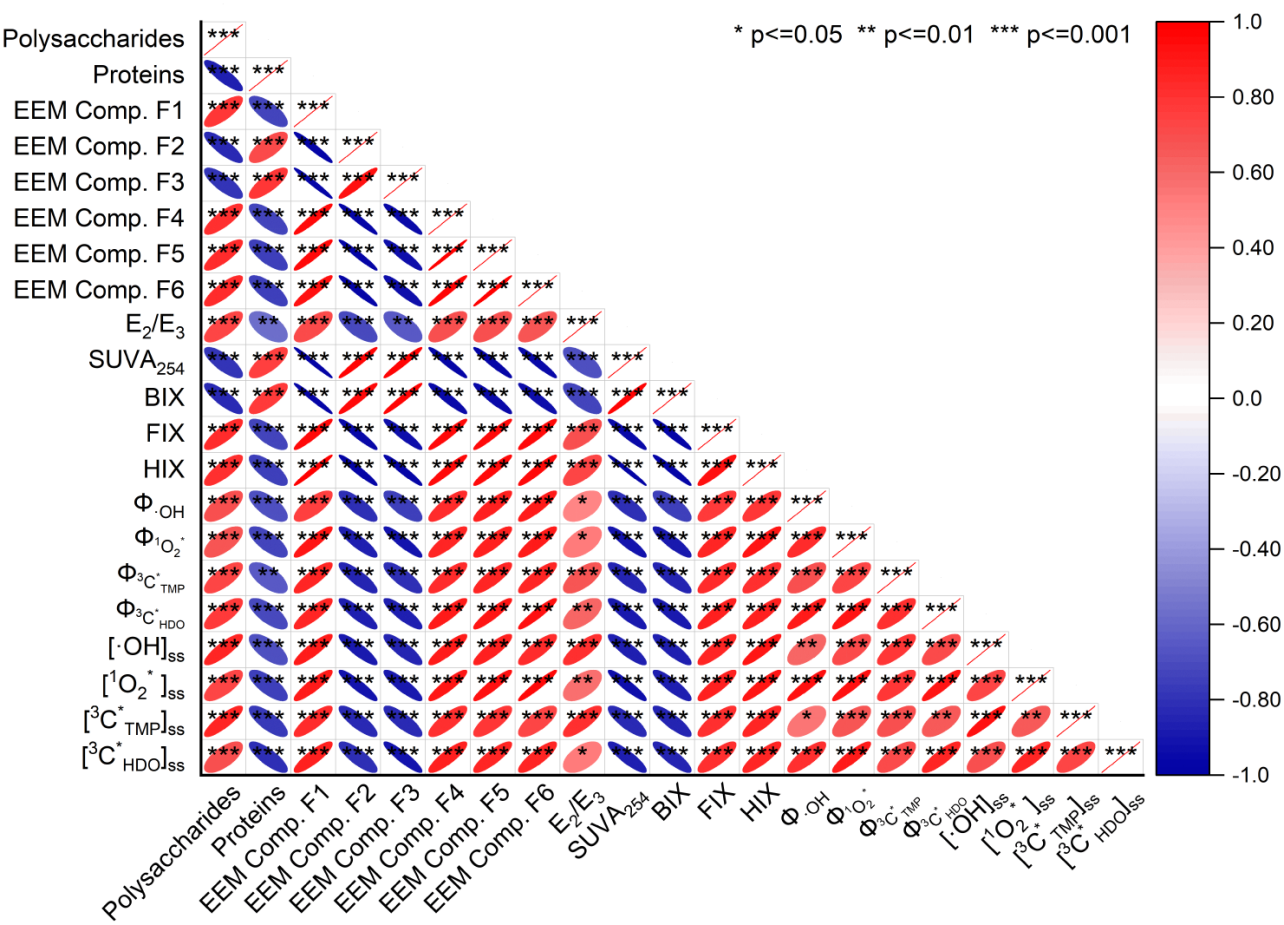
Correlation matrix between Φ_RI_, [RI]_ss_, protein and polysaccharide concentrations, optical and fluorescence parameters compiled from the bulk and MW-fractionated EPS samples extracted from culturable bacteria in PM_2.5_ (*p* < 0.05, *; *p* < 0.01, **; *p* < 0.001, ***). The color and size of the squares indicate the correlation coefficients. Red and blue indicate positive and negative correlations, respectively.

To assess the possibility of predicting Φ_RI_ and [RI]_ss_ from the aforementioned measurable optical and chemical characteristics, we developed empirical fits using MLR. This approach allowed us to quantify oxidant production from each contributing optical and chemical characteristics captured in the fit’s slope coefficient. All of the optical and chemical parameters for the bulk and MW-fractionated COM and EPS samples extracted from culturable bacteria in PM_2.5_ were combined together and used for MLR model development. The predictors were chosen using orthogonal partial least-squares analysis to eliminate systematic variation in the predictors that was not correlated with the response variables (Section S6).^89^ Φ_RI_ and [RI]_ss_ predicted using MLR models agreed well with the measured values (Figure S31), with *R* ^2^ values ranging from 0.70 to 0.85. The coefficients for the MLR fits highlighted the important roles of FIX and *E*_2_/*E*_3_ in predicting Φ_RI_ and [RI]_ss_ (Table S15), indicating that the degree of aromaticity and molecular size are key predictors of oxidant production.

### 3.4 Application of ML Models to Investigate Links between the Optical and Chemical Parameters of COM and EPS and Φ_RI_

Despite the promising predictive strengths demonstrated by the MLR models, they provided limited insights into the relationships between Φ_RI_/[RI]_ss_ and optical and chemical characteristics, which led to questions about their generalization abilities. Thus, ML models were also developed to investigate the complex relationships (e.g., positive vs negative, linear vs nonlinear vs monotonic) between Φ_RI_/[RI]_ss_ and the optical and chemical characteristics and for predictive modeling (Section S7). Due to its high cross-validation scores using default hyperparameters (Table S4), the CB algorithm was selected to identify important features and predictors of ln­(Φ_RI_) and ln­([RI]_ss_) from inputted optical and chemical parameters. The optimized ln­(Φ_RI_) and ln­([RI]_ss_) models demonstrated strong predictive performance on the test sets, with *R* ^2^ values between 0.76 and 0.90 (Figures S32 and S33). The similar *R* ^2^ and RMSE values between the test and cross-validation sets demonstrated the strong predictive performances of the optimized ln­(Φ_RI_) and ln­([RI]_ss_) models (Table S5). The positive/negative relationships between the different optical and chemical parameters and Φ_RI_ and [RI]_ss_ revealed by the PDP (Figures S34 and S35) were consistent with the results from Spearman’s rank correlation analysis (Figure 4). The PDP showed that many of the relationships between the key optical/chemical parameters and Φ_RI_/[RI]_ss_ were nonlinear.

We focused on the ln­(Φ_RI_) models to relate optical and chemical parameters to oxidant production efficiency. Feature importance rankings from tree-based feature importance analysis and SHAP showed that parameters related to the degree of aromaticity (i.e., FIX and SUVA_254_) and molecular size (i.e., *E*_2_/*E*_3_) had strong effects on the ln­(Φ_RI_) models’ predictive performances (Figure 5). This is consistent with the MLR modeling results (Table S15). FIX and E_2_/E_3_ were positively correlated with the Φ_RI_ predictions, whereas SUVA_254_ was negatively correlated (Figure S34). This suggests that photosensitizers of lower aromaticity and smaller molecular size produce oxidants more efficiently. The inverse relationship between molecular size and oxidant production efficiency could be explained by the occurrence of CT interactions within large molecules, which likely have multiple electron donor (e.g., hydroxylated groups) and acceptor (e.g., aromatic carbonyls) moieties present closely within the molecules.^87^ While electron acceptor moieties are key players in oxidant production (especially for ^1^O_2_* and oxidizing ^3^C*), electron donor and acceptor moieties can interact with each other in the ground or excited states through electron transfer to form CT complexes that may not produce oxidants during irradiation, thus leading to lower oxidant production efficiency.^87,88^ Conversely, smaller molecules have fewer CT interactions. Thus, fewer CT complexes are formed, resulting in smaller molecules having oxidant production efficiencies higher than those of larger molecules. The inverse relationship between the degree of aromaticity and oxidant production efficiency could be explained by the decrease in the aromatic moieties in molecules, resulting in lower light absorption rates compared to oxidant production rates (eqs S12, S16, S20, and S32) and consequently higher oxidant production efficiencies.

**Figure 5:**
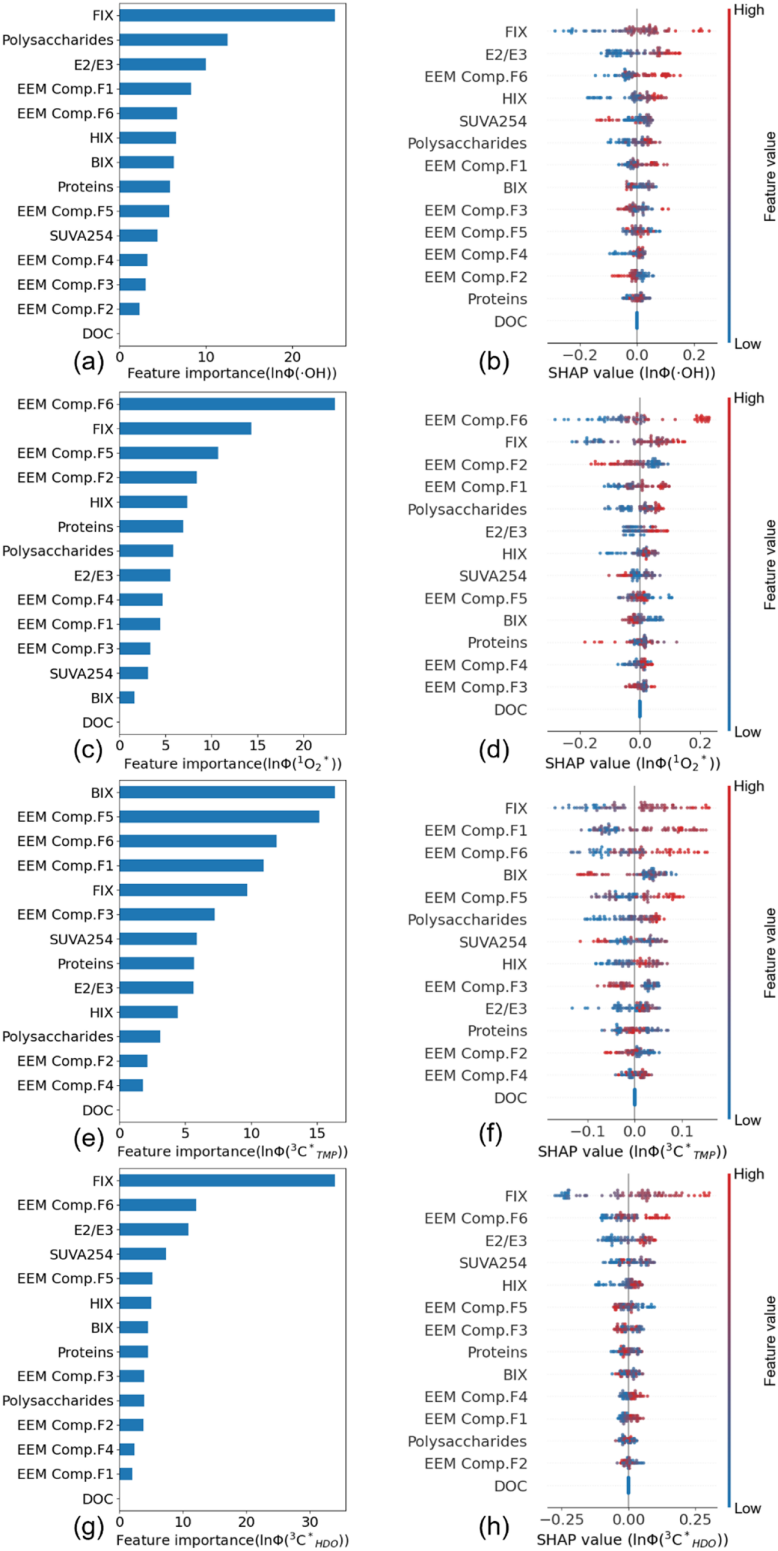
Feature importance rankings from tree-based feature importance and SHAP analysis of (a, b) ln­(Φ_•OH_), (c, d) ln(ΦO2*1) , (e, f) ln(ΦCTMP*1) , and (g, h) ΦCHDO*3 . Feature importance rankings for ln­([RI]_ss_) can be found in Figure S36. For the SHAP summary interpretation plots (b, d, f, and h), the *x*-axes are the SHAP values, and the *y*-axes are the features ranked based on their importance. A positive (negative) SHAP value of a feature in a scenario denotes it positive (negative) contribution to the model prediction. A greater absolute SHAP value implies a greater contribution to the model prediction. The scatter pattern for each feature is composed of the SHAP values for the training data in that feature.

Some fluorescence components (e.g., F6) were also identified as important features by tree-based feature importance analysis and SHAP. This suggested that some organic compounds that contributed to these fluorescence components serve as key photosensitizers for oxidant production. Although there have been some studies on the molecular characteristics of these fluorescence components,^40,71,72^ the molecular structures of these highly complex groups of unresolved fluorophores remain ambiguous. Additionally, not all fluorophores are photosensitizers,^62^ and the fraction of organic matter in PM that exhibits photosensitization and/or fluorescence remains unknown. While addressing these questions is outside the scope of this paper, such investigations are necessary to further enhance our overall understanding of oxidant production by photosensitizers from different aerosol sources and should be a focus of future studies.

## 4 Environmental Implications

Even though bioaerosols are ubiquitous in the atmosphere and can even dominate the total aerosol population in geographically isolated regions,^13–15^ many questions remain about how bioaerosols affect atmospheric processes and vice versa, particularly the roles of functionally diverse and metabolically active bacterial communities in the atmosphere. Despite the atmosphere being considered a harsh environment for bacteria, atmospheric bacteria possess transcripts related to different types of bacterial functions that can result in the biosynthesis and release of COM and EPS, which can contribute to atmospheric organic matter.^12,33–37^ However, little is currently known about the optical and chemical characteristics and photochemical behavior of this bacterial-derived organic matter. In this study, we investigated the optical and chemical characteristics, photochemical behavior, and oxidant production by aqueous extracts of COM and EPS from culturable bacteria from summer and winter PM_2.5_ collected at an urban site in Hong Kong. Despite having distinctive compositions and optical properties, COM and EPS demonstrated similar MW-dependent trends. Most noticeably, the molecular size and degree of aromaticity of chromophores and fluorophores in both COM and EPS increased with MW. ^3^C*, ^1^O_2_*, and •OH production occurred during the irradiation of COM and EPS. We showed that COM and EPS contained photosensitizers that are highly efficient at producing ^1^O_2_* and oxidizing ^3^C*, with Φ_RI_ values as high 10% and 5% measured for ^1^O_2_* and oxidizing ^3^C*, respectively. Φ_RI_ and [RI]_ss_ decreased with MW for both COM and EPS. This was due to higher MW molecules engaging in charge-transfer interactions that disrupted photochemical processes and oxidant production. Despite the complex nonlinear relationships that Φ_RI_ and [RI]_ss_ had with the measurable optical and chemical properties of COM and EPS, ML models were able to make robust predictions of Φ_RI_ and [RI]_ss_ using fluorescence and absorbance spectral parameters. Spectral parameters related to the degree of aromaticity and molecular size had the strongest effects on the models’ predictive performances.

The significance of our results lies foremost in our observations that atmospheric COM and EPS are efficient producers of aqueous-phase oxidants. This is especially the case for ^1^O_2_* and oxidizing ^3^C*, whose measured Φ_RI_ values are comparable to those for biomass burning aerosols, anthropogenic secondary organic aerosols, and cooking organic aerosols, which contain photosensitizers that produce oxidants efficiently.^5–8^ It is worth noting that only water-soluble COM and EPS extracted from culturable bacteria from PM_2.5_ were investigated in this study. It is possible that other forms of water-soluble and water-insoluble chromophoric organic matter derived from culturable and nonculturable bacteria are efficient producers of oxidants as well. There are some caveats that should be noted. First, we used high concentrations of bacteria to obtain sufficient quantities of COM and EPS for photochemistry experiments. The DOC concentrations (5 mg C/L) of COM and EPS used in the photochemistry experiments fall within the range of total DOC concentration (2–108.6 mg C/L) in ambient cloudwater in Hong Kong,^90^ which indicated that our experiments were conducted under dilute conditions akin to ambient cloud and fog, instead of ambient aerosols in which the DOC concentrations are higher.^4,91^ Despite the lack of ambient measurements, the DOC concentrations of COM and EPS used in our study are likely higher than those of COM and EPS in atmospheric aqueous phases. Previous studies showed that [RI]_ss_ increases with DOC concentration in extracts of ambient aerosols, albeit nonlinearly.^4,7,91^ The effects that the DOC concentration have on Φ_RI_ are less clear, with a single study reporting that only the formation rates of oxidizing ^3^C* (and hence ΦC*3 ) consistently changed with DOC concentration in extracts of 13 different ambient aerosol samples.^7^ Nevertheless, high DOC concentrations could potentially cause significant light screening and probe inhibition, in addition to enhanced photosensitization, all of which could affect Φ_RI_. Second, it is possible that not all bacteria produce EPS whenever they encounter stress, and some may only produce EPS under specific conditions. Additionally, different bacteria species likely produce different quantities of COM during different bacterial functioning processes. The diverse metabolically active bacterial communities present in the atmosphere complicates our ability to directly quantify COM and EPS in atmospheric aqueous phases even though several studies (in addition to ours) have reported the production of COM and EPS from bacteria isolated from ambient samples.^27,33,34^ Quantifying the extent of the photochemical processing of COM and EPS in the atmosphere would require a larger-scale field study to fully understand their implications and variabilities under diverse atmospheric conditions. Third, although this study highlighted the efficient production of aqueous-phase oxidants by COM and EPS from culturable atmospheric bacterial isolates, we were limited by the number of samples collected from a single urban site during two seasons. Even though our results showed the diversity of culturable bacteria in summer and winter (Figure S1), a previous study showed that the microbial composition in outdoor tropical air sampled from a single location exhibits a diel cycle.^92^ Thus, future studies could consider deploying temporal sampling at finer resolution in different types of locations (e.g., urban vs remote vs rural) across all four seasons in order to investigate the photochemical behaviors of COM and EPS produced by different compositions of atmospheric bacteria driven by seasonal and diel cycles. Fourth, while the use of culturable bacterial isolates is a valid approach for the initial investigation of the photochemical behavior of atmospheric bacterial-derived organic matter and its ability to produce aqueous oxidants, it may result in an underestimation of bacterial diversity and abundance due to the inherent biases of culture-based techniques that favor the growth of certain bacterial taxa over others. Nevertheless, we expect that many of the insights obtained from this study can also be applicable to nonculturable bacterial COM and EPS, owing to the apparent similarities in their chemical composition. Overall, our results indicate that bacterial-derived organic matter can potentially serve as significant contributors to photochemical reactions in atmospheric aqueous phases and interfaces through their ability to produce oxidants in areas where bioaerosols comprise a substantial fraction of the aerosol population. Although the typical fractions by which organic matter in atmospheric aerosols are comprised of bacterial-derived organic matter remain unknown, data compiled by Fröhlich-Nowoisky et al. (2016) indicate that bioaerosols contribute about 30% of the aerosol load in urban and rural air and up to 80% of the aerosol load in clean rainforest air.^14^

Our results also highlighted the possibility of applying ML models to reveal complex relationships that Φ_RI_ and [RI]_ss_ have with measurable optical and chemical properties. Parameters related to the degree of aromaticity and molecular size were found to have strong (albeit nonlinear) effects on the ln­(Φ_RI_) models’ predictive performances. Through these relationships, we deduced that photosensitizers of lower aromaticity and smaller molecular size produce oxidants more efficiently. Despite these promising results, the exact molecular structures of photosensitizers that produce oxidants remain ambiguous due to the limitations of these absorbance and fluorescence techniques. Hence, it is necessary to combine these spectroscopic tools with other analytical methodologies (e.g., mass spectrometry) to develop a molecular-level understanding of the subsets of chromophoric compounds that serve as photosensitizers and form oxidants in atmospheric aqueous phases and interfaces. Nevertheless, we show that ML models can make robust predictions of Φ_RI_ and [RI]_ss_. Although the focus of our study is on atmospheric COM and EPS, we anticipate that ML models can similarly be applied to the more commonly investigated water-soluble fractions in PM. Thus, there is an opportunity to combine absorbance and EEM fluorescence data sets with ML models to identify key optical and chemical parameters that govern oxidant production in aqueous PM, as well as to make robust predictions of Φ_RI_ and [RI]_ss_ based on these parameters. This will be especially helpful for determining Φ_RI_ values for a large number of PM samples from different sources while reducing the amount of time spent on photochemistry experiments.

## Supplementary Material



## Data Availability

The data and source codes can be accessed upon request (theodora.nah@cityu.edu.hk).

## Notes

Additional experimental details, materials, and methods; additional details on machine learning model development, evaluation, and interpretation; additional details, data, and figures on the composition and optical properties of bulk and MW-fractionated COM and EPS from culturable bacteria in PM_2.5_ and model bacteria strains; additional data and figures of Φ_RI_ and [RI]_ss_ of •OH, ^1^O_2_*, and ^3^C* produced from bulk and MW-fractionated COM and EPS from culturable bacteria in atmospheric PM_2.5_ and from pure laboratory bacterial cultures (PDF)
