# Supplementary material for: Efficient Production of Reactive Oxidants by Atmospheric Bacterial-Derived Organic Matter in the Aqueous Phase
Source: Environ Sci Technol. 2025 Mar 28;59(13):6757–70. doi: 10.1021/acs.est.5c01526 (PMC12709572; doi:10.1021/acs.est.5c01526)
Supplement: Supplementary file 1 [file es5c01526_si_001.pdf]

Supplementary Information

Efficient production of reactive oxidants by atmospheric bacterial-derived organic matter in the aqueous phase

Yushuo Liu [a,b], Yitao Li [a,b], Wing Lam Chan [a], Yingyu Bao [a], Patrick K. H. Lee [a,c], and Theodora Nah [a,b,c*]

[a] *School of Energy and Environment, City University of Hong Kong, Hong Kong SAR, China*
[b] *City University of Hong Kong Shenzhen Research Institute, Shenzhen 518057, China*
[c] *State Key Laboratory of Marine Pollution, City University of Hong Kong, Hong Kong SAR, China*

* Email: theodora.nah@cityu.edu.hk

Summary: 67 pages, 6 text sections, 41 figures, 15 tables

Section S1. Chemicals and reagents

Chemicals were used as received unless noted. Ultrapure water (Milli-Q®, Merck, 18.2 MΩ-cm) was used to prepare all the solutions used for bacteria cultivation, sample preparation, experiments, and chemical analysis. Acetonitrile (ACN, HPLC grade) was purchased from RCI Labscan Co. Ltd. (Thailand). Isopropanol (IPA, LCMS grade) was purchased from Optima (USA). Formic acid (FA, Analytical grade) and benzene (99.5%, Analytical grade) were purchased from J&K Scientific (China). Furfuryl alcohol (FFA, 98%) was purchased from Acros Organics (USA), and was redistilled before use. *p*-nitroanisole (PNA, 97%), pyridine (pyr, 99.8%), phenol (~99%), 2,4,6-trimethylphenol (TMP, 97%), and trans,trans-2,4-Hexadien-1-ol (t,t-HDO, 97%) were purchased from Sigma-Aldrich (USA). The Luria-Bertani broth (LB) culture medium contained 1 g tryptone, 0.5 g yeast extract, and 1 g NaCl in 100 mL of water. The Nutrient Broth (NB) culture medium contained 0.5 g peptone, 0.3 g meat extract in 100 mL of water. The Yeast Extract Peptone Dextrose medium (YPD) culture medium contained 1 g peptone, 0.5 g yeast extract, and 1 g NaCl in 100 mL of water. The Tryptic Soy Broth (TSB, BD Bacto™) was purchased from Fisher Scientific Co. Ltd. (USA), and it contained 3 g of powder in 100 mL water.

Section S2. DNA extraction, amplicon sequencing and bioinformatic analysis

The genomic DNA was extracted from the cultured bacteria using the GeneJET Genomic DNA Purification Kit (Thermo Fisher Scientific, USA) following the manufacturer's protocols. Amplification of the 16S rRNA gene was carried out using the 515F/806R primer pair.[1, 2] The sequencing was performed on an Illumina MiSeq platform (Novogene Technology Co., Ltd., China), producing 250 bp paired-end reads. The sequence data has been deposited in the Sequence Read Archive of the National Center for Biotechnology Information (NCBI) under accession number PRJNA1152763.

The sequencing reads were analyzed using the QIIME2 platform (v2021.11),[1] following previously described methods.[3, 4] Briefly, reads shorter than 240 bp and with an average Phred quality score below 25 were discarded. The remaining reads were processed with the DADA2 plugin in QIIME2 for denoising, merging paired-end reads, removing chimeras, and generating amplicon sequence variants (ASVs).[5] An average of 90,299 ± 16,857 paired-end reads per sample was used for downstream analysis of the summer samples, while 99,119 ± 23,179 paired-end reads per sample were used for the winter samples. Taxonomic classification of ASVs was performed using the SILVA 138 reference database (99 %

similarity).[6] The blank samples that were processed in parallel with the field samples as negative controls did not yield a sufficient quantity of DNA for sequencing. The relative abundance of ASVs was calculated using the "dplyr" package (v.1.0.8) in R (v.4.1.2).

Section S3. Extraction of the bacterial cellular organic matter (COM) and extracellular polymeric substances (EPS)

The COM were obtained using the ultrasonic method: The washed bacterial pellets were resuspended in ultrapure water and then lysed by sonication (Ultrasonic Cell Crusher, Lichen Co., China) at 400 W for 10 min (3 sec working, 2 sec free) in an ice water bath. The supernatant was then obtained as a raw COM solution and separated from the cell debris via centrifugation at 10,000g for 20 min. The EPS were collected using a modified heating extraction method: The washed bacterial pellets were resuspended in ultrapure water and heated in a 60 °C water bath for 30 min. The supernatant was obtained as a raw EPS solution via centrifugation at 10,000g for 20 min. Both the resulting raw COM and EPS solutions were filtered through 0.22 μm membrane filters to remove the unsettled cells. All the COM and EPS solutions were stored at 4 °C in the dark prior to use for experiments and chemical analysis.

The Bradford method was used to quantify the protein concentrations in the COM and EPS samples. Bovine serum albumin (BSA) and Coomassie Brilliant Blue were used as the standard and colorant, respectively. The colorant was mixed with the COM/EPS samples and the absorbance at 595 nm was measured using a UV–Visible spectrophotometer. The protein concentrations were subsequently determined by comparing the measured absorbance against the calibration curve of the BSA standard. The polysaccharide concentrations in the COM and EPS samples were determined using the phenol-sulfuric acid method with glucose as the standard. Briefly, 5 % phenol and sulfuric acid were mixed at a volume ratio of 1:5 to form the colorant, which was then cooled in ice water. The COM/EPS samples were then mixed with the colorant at a ratio of 1:3 (v/v), and the absorbance at 490 nm was measured using a UV–Visible spectrophotometer.

Section S4. Excitation-emission matrix (EEM) fluorescence measurements

The excitation and emission wavelength ranges were from 220 to 500 nm (5 nm step) and 200 to 600 nm (10 nm step), respectively. The choice of wavelength spacing was selected based on previous similar studies of this nature.[7-9] The fluorescence spectra were obtained in the "Signal/Reference" mode with instrumental bias correction. The standard practices for

EEM fluorescence spectra collection and processing were used. The inner filter effect was corrected by the absorbance spectrum measured with the UV–Visible spectrophotometer.[10] The EEM fluorescence spectra of all the COM and EPS samples were corrected by subtracting the blank sample. Interpolation was used to remove the first- and second-order Rayleigh scattering. Parallel factor analysis (PARAFAC) with non-negativity constraint was performed on all the EEM spectra using the R package staRdom version 1.1.25.[11] Six components were identified based on comparisons of their extracted spectra to those reported in previous studies.[12-15] These six components were identified as Flavin-like, Tryptophan-like, Tyrosine-like, HULIS-1, fulvic-like, and HULIS-2 (Figure S2 and Table S2).

Section S5. Determination of the formation rates (R_{RI}), quantum yields (Φ_{RI}), and steady-state concentrations ($[RI]_{ss}$) of the photooxidants from photochemistry experiments using chemical probes

The UV-Visible spectra data was used for the calculation of the light absorption rates (R_a) for the COM/EPS samples:

$$R_a = \frac{\sum_\lambda \mathrm{I}(\lambda)\times\Delta\lambda\times(1-10^{-\alpha_\lambda l})}{l} \text{(S1)}$$

where I(λ) is the absolute spectral irradiance (mol-photons cm^{-2} s^{-1} nm^{-1}), which is shown in Figure S3, $\Delta\lambda$ is the wavelength interval (1 nm), and α_λ is the decadic absorption coefficient of the samples, which can be calculated as follows:

$$\alpha_\lambda = \frac{A(\lambda)}{l} = \frac{a(\lambda)}{2.303} \text{(S2)}$$

where A(λ) is the measured absorbance at wavelength λ nm, l is the pathlength of cuvette (0.01 m), and $a(\lambda)$ is the Napierian absorption coefficient. Noted that equation S1 takes the self-screening of chromophoric dissolved organic matter into consideration.[16]

The concentrations of phenol (product from ·OH + benzene), FFA, TMP, t,t-HDO and c,c-HDO (products from $^3C^*$ + HDO) were determined using ultrahigh performance liquid chromatography coupled with a photodiode array detector (UPLC-PDA). The instrumental methods are shown in Table S3.

To characterize the photogeneration of ·OH, benzene of different initial concentrations was spiked into the samples: $[benzene]_0$ = 1 mM, $[benzene]_0$ = 1.5 mM, $[benzene]_0$ = 2 mM, $[benzene]_0$ = 3 mM. The following equations were subsequently used:[17]

$$\frac{d[\cdot OH]}{dt} = 0 = R_{\cdot OH} - \sum k_{Si}[\cdot OH]_{ss}[S_i] - k_{benzene,\ \cdot OH}[\cdot OH]_{ss}[benzene] \quad (S3)$$

$$[\cdot OH]_{ss} = \frac{R_{\cdot OH}}{\sum k_{Si}[S_i] + k_{benzene,\ \cdot OH}[benzene]} \quad (S4)$$

$$R_{phenol} = Y \times k_{benzene,\ \cdot OH} \times [\cdot OH]_{ss} \times [benzene] = \frac{R_{\cdot OH} \times Y \times k_{benzene,\ \cdot OH} \times [benzene]}{\sum k_{Si}[S_i] + k_{benzene,\ \cdot OH}[benzene]} \quad (S5)$$

$$\frac{1}{R_{phenol}} = \frac{\sum k_{Si}[S_i]}{R_{\cdot OH} \times Y \times k_{benzene,\ \cdot OH}} \times \frac{1}{[benzene]} + \frac{1}{R_{\cdot OH} \times Y} \quad (S6)$$

where $R_{\cdot OH}$ is the formation rate of ·OH (M s^{-1}), $\sum k_{Si}[S_i]$ is the scavenge rate constant of ·OH by natural sinks in the sample (s^{-1}), $k_{benzene,\ \cdot OH}$ is the second-order rate constant between benzene and ·OH ($k_{benzene,\ \cdot OH}$= 7.8×10^9 M^{-1} s^{-1}),[18] $[\cdot OH]_{ss}$ is the steady-state concentration of ·OH (M), and [benzene] is the concentration of benzene. Y is the yield of phenol from the reaction of benzene with ·OH (0.73).[19] By plotting $\frac{1}{R_{phenol}}$ vs. $\frac{1}{[benzene]}$, $R_{\cdot OH}$, $[\cdot OH]_{ss}$, and $\sum k_{Si}[S_i]$ could subsequently be calculated as follows:[20, 21]

$$R_{\cdot OH} = \frac{1}{y\text{-}intercept \times Y} \quad (S7)$$

$$\sum k_{Si}[S_i] = \frac{k_{benzene,\ \cdot OH} \times slope}{y\text{-}intercept} \quad (S8)$$

$$[\cdot OH]_{ss} = \frac{1}{k_{benzene,\ \cdot OH} \times slope \times Y} \quad (S9)$$

For R_{phenol}, the plot of the concentration of phenol ([phenol]) vs. reaction time (t) was first fitted with the following equation:

$$[Phenol] = a \times (1 - e^{-bt}) \quad (S10)$$

R_{phenol} was then calculated as follows:

$$R_{phenol} = a \times \quad (S11)$$

The apparent quantum yields of ·OH ($\Phi_{\cdot OH}$, mol mol-photons^{-1}) was calculated as follows:

$$\Phi_{\cdot OH} = \frac{R_{\cdot OH}}{R_a} \quad (S12)$$

To characterize the photogeneration of $^1O_2^*$, 10 μM FFA was spiked into the samples. The effect of the concentration of added FFA on the concentration of $^1O_2^*$ photogenerated were observed to be negligible (<5 % variation in FFA-quenching $^1O_2^*$ with $[FFA]_0$ < 145 μM).[16]

Direct photolysis and dark reactions of FFA were also negligible on the time scale of our experiments. For the reactivity of FFA with ·OH, 10 mM isopropanol was added as a ·OH quencher.[16] The pseudo-first order rate constants for the degradation of FFA ($k_{obs,FFA}$, s^{-1}) were determined from the loss of FFA:

$$-\frac{d[FFA]}{dt} = k_{obs,FFA}[FFA] = k_{FFA,\ ^1O_2}[FFA][^1O_2^*]_{ss} \quad (S13)$$

$$[^1O_2^*]_{ss} = \frac{k_{obs,FFA}}{k_{FFA,\ ^1O_2}} \quad (S14)$$

$$R_{^1O_2^*} = [^1O_2^*]_{ss} \times k_d^{\Delta} \quad (S15)$$

$$\Phi_{^1O_2^*} = \frac{R_{^1O_2^*}}{R_a} \quad (S16)$$

where, $k_{obs,FFA}$ is the pseudo-first order rate constants for the degradation of FFA (s^{-1}), $k_{FFA,^1O_2^*}$ is the second-order reaction rate constant of FFA with $^1O_2^*$ (1.0×10^8 M^{-1} s^{-1}),[22] $[^1O_2^*]_{ss}$ is the steady-state concentration of $^1O_2^*$ (M^{-1}), $R_{^1O_2^*}$ is the formation rate of $^1O_2^*$ (M s^{-1}), k_d^{Δ} is the aqueous singlet oxygen deactivation rate constant ($k_d^{\Delta} = 2.78\times10^5$ s^{-1}, $\tau_{\Delta} = 3.5$ μs at 25 °C),[23] and $\Phi_{^1O_2^*}$ is the apparent quantum yields of $^1O_2^*$ (mol mol-photons^{-1}).

To characterize the photogeneration of electron-transferred $^3C^*$, 10 μM TMP was spiked into the samples as the electron-transferred probe of $^3C^*$. Direct photolysis and dark reactions of TMP were negligible on the time scale of our experiments. The pseudo-first order rate constants for the degradation of TMP ($k_{obs,TMP}$, s^{-1}) were determined from the loss of TMP:

$$-\frac{d[TMP]}{dt} = k_{obs,TMP}[TMP] = k_{TMP,^3C_{TMP}^*}[TMP]\left[^3C_{TMP}^*\right]_{ss}$$

$$+ k_{TMP,^1O_2}[TMP][^1O_2^*]_{ss} + k_{TMP,\bullet OH}[TMP][\bullet OH]_{ss} \quad (S17)$$

$$\left[^3C_{TMP}^*\right]_{ss} = \frac{k_{obs,TMP}^{C}}{k_{TMP,^3C_{TMP}^*}} \quad (S18)$$

$$R_{^3C_{TMP}^*} = \left[^3C_{TMP}^*\right]_{ss} \times k_{q,^3C_{TMP}^*} \quad (S19)$$

$$\Phi_{^3C_{TMP}^*} = \frac{R_{^3C_{TMP}^*}}{R_a} \quad (S20)$$

where, $k_{obs,TMP}$ is the pseudo-first order rate constant for the degradation of TMP (s^{-1}), and

$k_{TMP,^3C^*_{TMP}}$ is the second-order reaction rate constant of TMP with $^3C^*_{TMP}$ ($3 \times 10^9\ M^{-1}s^{-1}$).[24, 25] $[^3C^*_{TMP}]_{ss}$ is the steady-state concentration of $^3C^*_{TMP}$ (M^{-1}). $k^c_{obs,TMP,}$ is the corrected pseudo-first order rate constant for the degradation of TMP due to $^3C^*_{TMP}$ using Equations S21 and S22. $R_{^3C^*_{TMP}}$ is the formation rate of $^3C^*_{TMP}$ ($M\ s^{-1}$). $k_{q,^3C^*_{TMP}}$ is the $^3C^*_{TMP}$ quenching rate constant due to dissolved oxygen and non-oxygen pathways ($k_{q,^3C^*_{TMP}} = 3 \times 10^5\ s^{-1}$ [26]). $\Phi_{^3C^*_{TMP}}$ is the apparent quantum yields of $^3C^*_{TMP}$ (mol mol-photons^{-1}).

$$k^c_{obs,\,TMP} = \frac{k_{obs,TMP} - k_{TMP,\,\cdot OH}[\cdot OH]_{ss} - k_{TMP,\,^1O_2}[^1O_2]_{ss}}{IF_{TMP}} \quad (S21)$$

$$\frac{1}{IF_{TMP}} = 0.021[DOC] + 0.965 \quad (S22)$$

where $k_{TMP,\cdot\cdot OH}$ is the second-order reaction rate constant of TMP with ·OH ($1.6 \times 10^{10}\ M^{-1}s^{-1}$),[18] $k_{TMP,^1O^*_2}$ is the second-order reaction rate constant of TMP with $^1O_2$ ($5.1 \times 10^7\ M^{-1}s^{-1}$),[27] IF_{TMP} is the predicted inhibition factor induced by chromophoric dissolved organic matter in samples.[28, 29]

t, t-HDO was selected as the chemical probe for energy-transferred $^3C^*$. Different concentrations of t, t-HDO was spiked into the samples: $[\text{t,t-HDO}]_0 = 0.025$ mM, $[\text{t, t-HDO}]_0 = 0.05$ mM, $[\text{t, t-HDO}]_0 = 0.1$ mM, $[\text{t, t-HDO}]_0 = 0.2$ mM, and $[\text{t, t-HDO}]_0 = 0.5$ mM. It should be noted that the direct photodegradation of t, t-HDO under sunlight is negligible.[30] Photo-isomerization and the formation of c, c-HDO were monitored. Concentrations of c, c-HDO were determined from a calibration curve of t, t-HDO and the molar absorption coefficient correction at 230 nm relative to t, t-HDO ($a_{\text{t,t-HDO}} = (1.72\pm0.09) \times 10^4\ M^{-1}cm^{-1}$, $a_{\text{c,c-HDO}} = (1.36\pm0.05)\times10^4\ M^{-1}$).[30] The following equations were subsequently used:

$$R_{^3C^*_{HDO}} = \sum k_{S'_i}[^3C^*_{HDO}]_{ss}[S'_i] - k_{t,\,t-HDO,\,^3C^*_{HDO}}[^3C^*_{HDO}]_{ss}[t,t-HDO] \quad (S23)$$

$$[^3C^*_{HDO}]_{ss} = \frac{R_{^3C^*_{HDO}}}{\sum k_{S'_i}[S'_i] + k_{t,\,t-HDO,\,^3C^*_{HDO}}[t,\,t-HDO]} \quad (S24)$$

$$R_{c,c-HDO} = Y' \times k_{t,\,t-HDO,\,^3C^*_{HDO}}[t,t-HDO] \times [^3C^*_{HDO}]_{ss}$$

$$= \frac{Y' \times k_{t,\,t-HDO,\,^3C^*_{HDO}}[t,\,t-HDO] \times R_{^3C^*_{HDO}}}{\sum k_{S'_i}[S'_i] + k_{t,\,t-HDO,\,^3C^*_{HDO}}[t,\,t-HDO]} \quad (S25)$$

$$\frac{1}{R_{c,c-HDO}} = \frac{\sum \mathrm{k}_{S_i'}[S_i']}{Y' \times k_{t,\,t-HDO,\,^3C^*_{HDO}} \times R_{^3C^*_{HDO}}} \times \frac{1}{[t,\,t-HDO]} + \frac{1}{Y' \times R_{^3C^*_{HDO}}} \text{(S26)}$$

where $R_{^3C^*_{HDO}}$ is the formation rate of $^3C^*_{HDO}$ (M s^{-1}), $\sum \mathrm{k}_{S_i'}[S_i']$ is a scavenge rate constant of $^3C^*_{HDO}$ by natural sinks in the sample (s^{-1}), $k_{t,\,t-HDO,\,^3C^*_{HDO}}$ is the second-order rate constant between t,t-HDO and $^3C^*_{HDO}$ ($k_{t,\,t-HDO,\,^3C^*_{HDO}}$= (1.1 ± 0.1) × 10^9 M^{-1} s^{-1}) [30], [$^3C^*_{HDO}$]$_{ss}$ and [t,t-HDO] are the steady-state concentrations of [$^3C^*_{HDO}$]$_{ss}$ (M) and the concentrations of t, t-HDO, respectively. Y' is the yield of c,c-HDO from the reaction of t, t-HDO with $^3C^*_{HDO}$ (0.07).[30] By plotting $\frac{1}{R_{c,c-HDO}}$ vs. $\frac{1}{[t,\,t-HDO]}$, $R_{^3C^*_{HDO}}$, [$^3C^*_{HDO}$]$_{ss}$, and $\sum \mathrm{k}_{S_i'}[S_i']$ were determined using the following equations:

$$R_{^3C^*_{HDO}} = \frac{1}{y-intercept \times Y'} \text{(S27)}$$

$$\sum \mathrm{k}_{S_i'}[S_i'] = \frac{k_{t,\,t-HDO,\,^3C^*_{HDO}} \times slope}{y-intercept} \text{(S28)}$$

$$\left[{}^3C^*_{HDO}\right]_{ss} = \frac{1}{k_{t,\,t-HDO,\,^3C^*_{HDO}} \times slope \times Y'} \text{(S29)}$$

For $R_{c,c-HDO}$, the plot of the concentration of c, c-HDO ([c,c-HDO]) vs. reaction time (t) was first fitted with the following equation:

$$[c,c - HDO] = a \times (1 - e^{-bt}) \text{(S30)}$$

The $R_{c,c-HDO}$ was calculated using the following equation:

$$R_{c,c-HDO} = a \times \text{(S31)}$$

The apparent quantum yields of $^3C^*_{HDO}$ ($\Phi_{^3C^*_{HDO}}$, mol mol-photons^{-1}) was calculated using the following equation:

$$\Phi_{^3C^*_{HDO}} = \frac{R_{^3C^*_{HDO}}}{R_a} \text{(S32)}$$

Section S6. Development of MLR models for Φ_{RI} and [RI]$_{ss}$ predictions

The polysaccharides and protein concentrations, E_2/E_3, $SUVA_{254}$, BIX, HIX, and FIX of the bulk and MW-fractionated COM and EPS samples from culturable bacteria in $PM_{2.5}$ were combined together for MLR model development. Orthogonal partial least squares (OPLS) analysis was performed with SIMCA 14.1 to eliminate systematic variation in the predictors

that was not correlated with the response variables.[31] The significance of a predictor variable in explaining the response variables was determined by its variable importance in the projection (VIP) score, with scores above 1.0 being the most influential.[7, 32, 33] MLR analysis was conducted using Prism 8 through stepwise selection of OPLS-prioritized predictor variables until all variance inflation factors (VIF) were reduced to below 5.0, and parsimony measures were minimized. The intercepts of the fits were set to 0.

Section S7. Evaluation and interpretation of the ML models

The polysaccharides and protein concentrations, E_2/E_3, $SUVA_{254}$, BIX, HIX, and FIX of the bulk and MW-fractionated COM and EPS samples from culturable bacteria in $PM_{2.5}$ were combined together for ML model development. The combined datasets were then randomly divided into two parts: the training set and the test set. The training set constituted 80 % of the dataset, and it was used to develop the ML models. The test set constituted the remaining 20 % of the dataset, and it was used to evaluate the performance of the model. During the model development and evaluation stages, two metrics, the coefficient of determination (R^2) and root mean square error (RMSE), were utilized to evaluate the predictive performance of the model on either the training or test set. The higher the R^2 value and the lower the RMSE value, the better the predictive performance of the model.[34] During the development process, a 5-fold cross-validation method was applied to the training set to improve the predictive and generalization ability of the model.

ML models are essentially black-boxes; thus, it is difficult to understand the internal mechanisms of the model and explain how the model makes predictions. Instead, the model could be interpreted by analyzing the relationships between feature-target pairs to determine the importance or contribution of each input feature to the model's predictions. Here, the models were interpreted from different perspectives using tree-based feature importance analysis, the partial dependence plot (PDP), and Shapley's additive interpretation (SHAP).[35-39] As explained in detail by Liao et al. (2023),[39] the tree-based feature importance, which is a built-in function in the ML algorithm, is determined from the amount by which each feature split point enhances the performance metrics and is weighted by the number of observations that the feature accounts for. A greater improvement of the performance metric denotes a higher importance of the feature in the model. PDP analyzes how the input features affect the predictive target by mapping the effect of the selected feature to reveal the relationship (e.g., positive vs. negative, linear vs. nonlinear vs. monotonic) between the predictive target and each

feature. SHAP assesses the contribution of each feature to the model prediction by calculating the average absolute SHAP value of all the training scenarios in each feature. A positive (negative) SHAP value of a feature in a scenario denotes it positive (negative) contribution to the model prediction. A greater absolute SHAP value implies a greater contribution to the model prediction. The conclusions drawn from these three interpretation methods were validated to determine whether the predictions of the optimized model were consistent with the existing domain knowledge. Data processing and analysis as well as ML model development and interpretation were performed in Python (version 3.11).

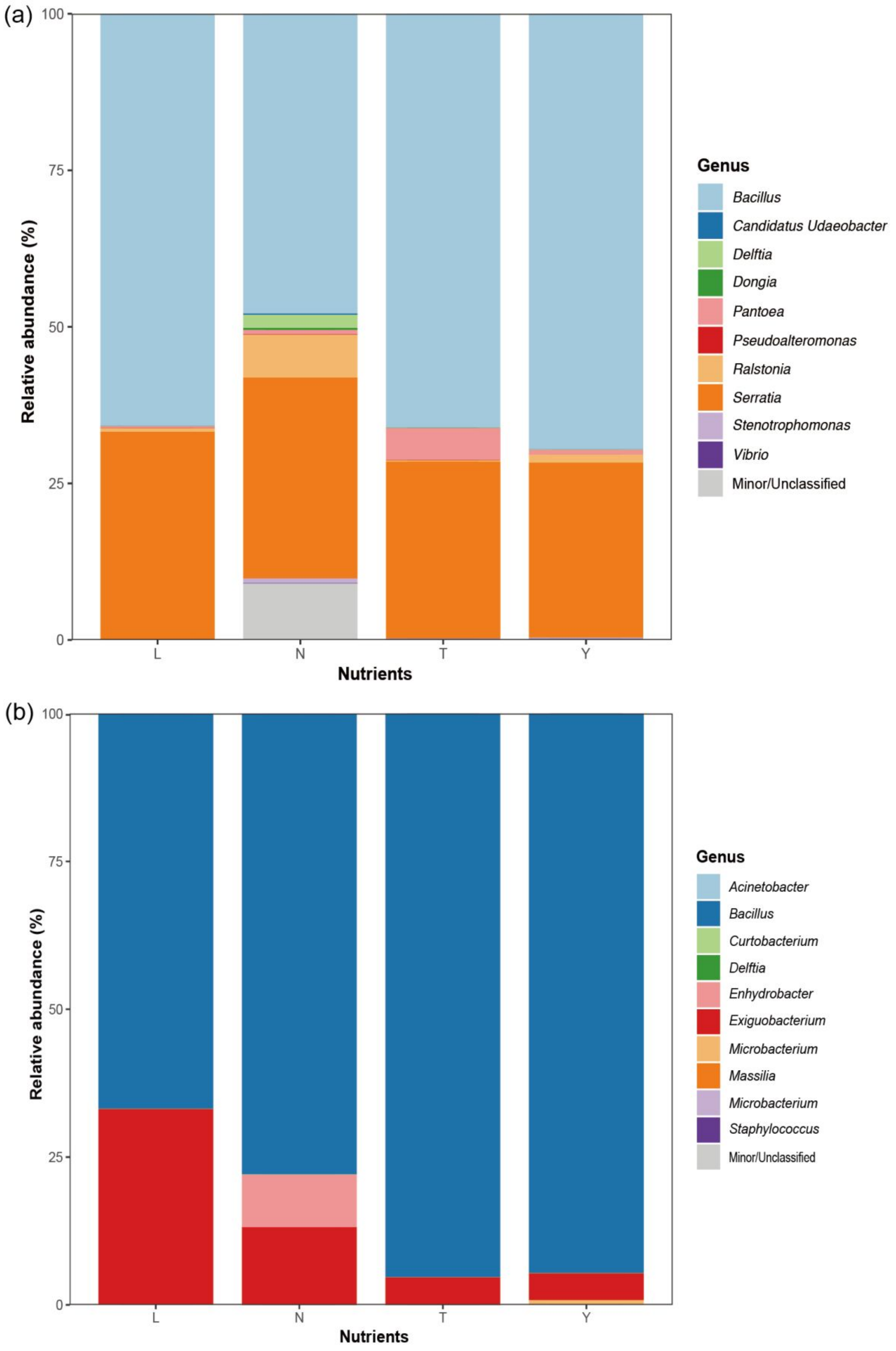

Figure S1. The average relative abundances of the top 10 genera in the bacterial community cultured from (a) summer and (b) winter $PM_{2.5}$ samples using four different culture media (N: Nutrient broth, L: Luria-Bertani broth, T: Tryptic Soy Broth, and Y: Yeast Extract Peptone Dextrose).

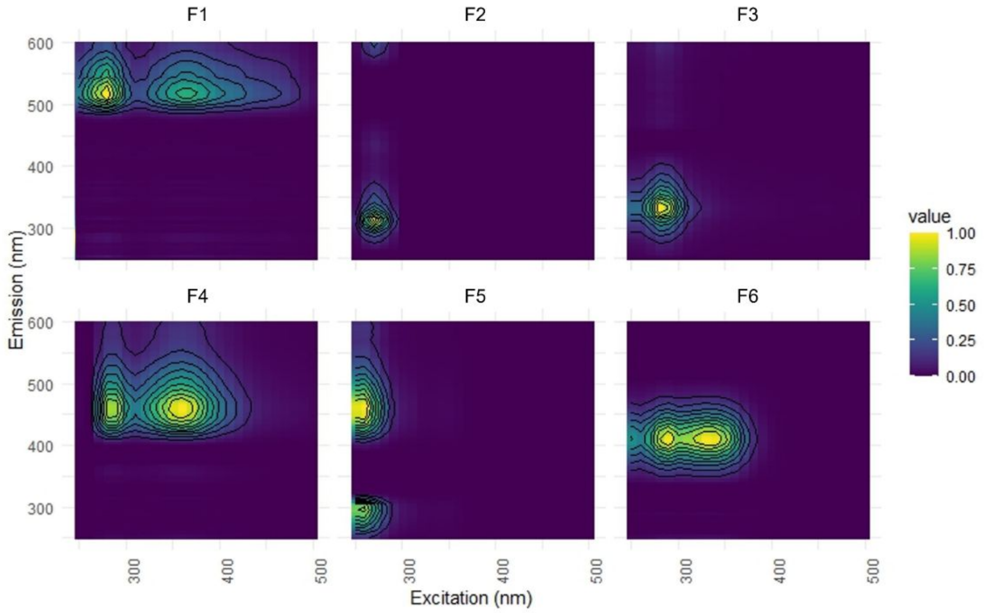

Figure S2. EEM contour plots of the six components identified by PARAFAC analysis.

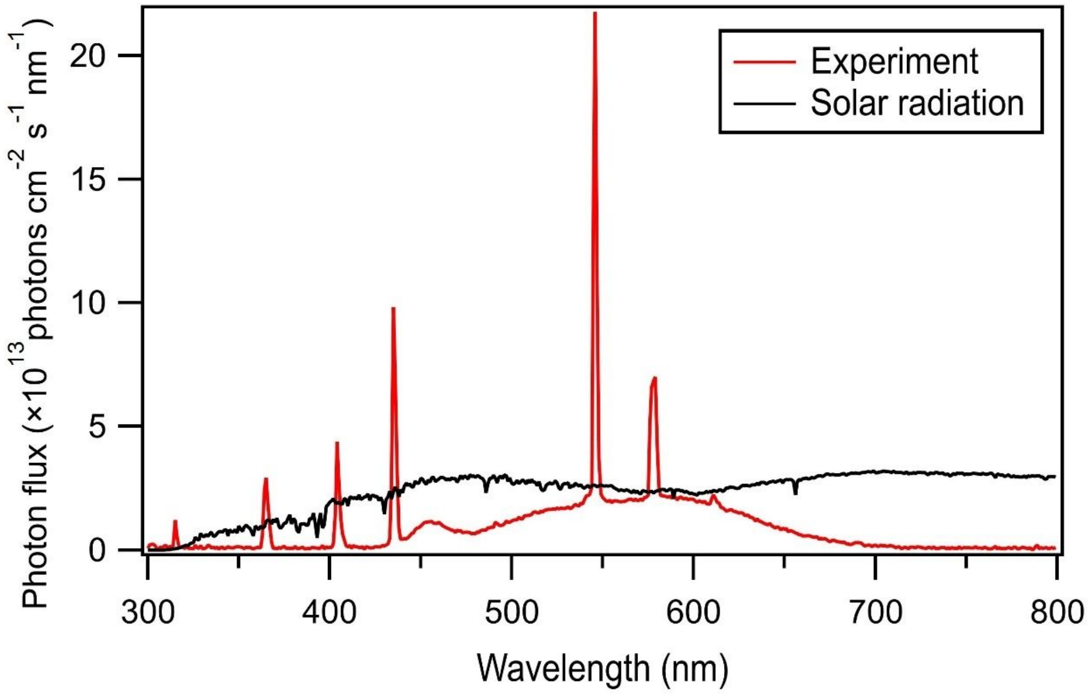

Figure S3. Photon flux inside of the quartz tubes (black) under our experimental conditions, and the actinic flux for a fall day in Hong Kong in the morning simulated using the "Quick TUV Calculator" available at https://www.acom.ucar.edu/Models/TUV/Interactive_TUV/ (Atmospheric Chemistry Observation & Modeling 5.3 version, National Center for Atmospheric Research) (red). The photon flux inside of the quartz tubes were determined via chemical actinometry PNA/pyr using the protocol detailed in our previous study.[7]

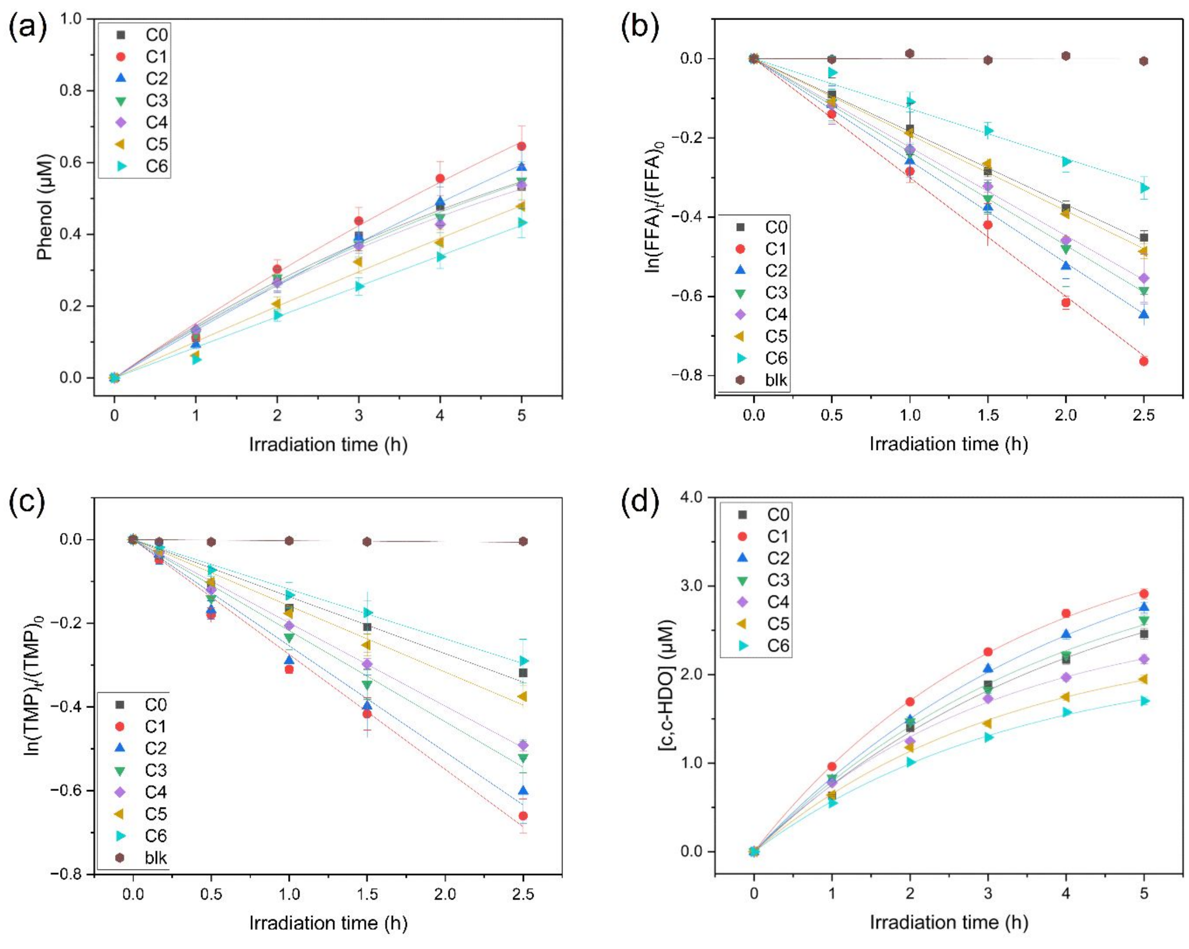

Figure S4. Examples of the types of measurements and kinetic analyses performed during the photochemistry experiments using different chemical probes: (a) formation of phenol using different initial concentrations of benzene, (b) pseudo first-order fits of the FFA decays, (c) pseudo first-order fits of the TMP decays, and (d) formation of c,c-HDO using different initial concentrations of t, t-HDO.

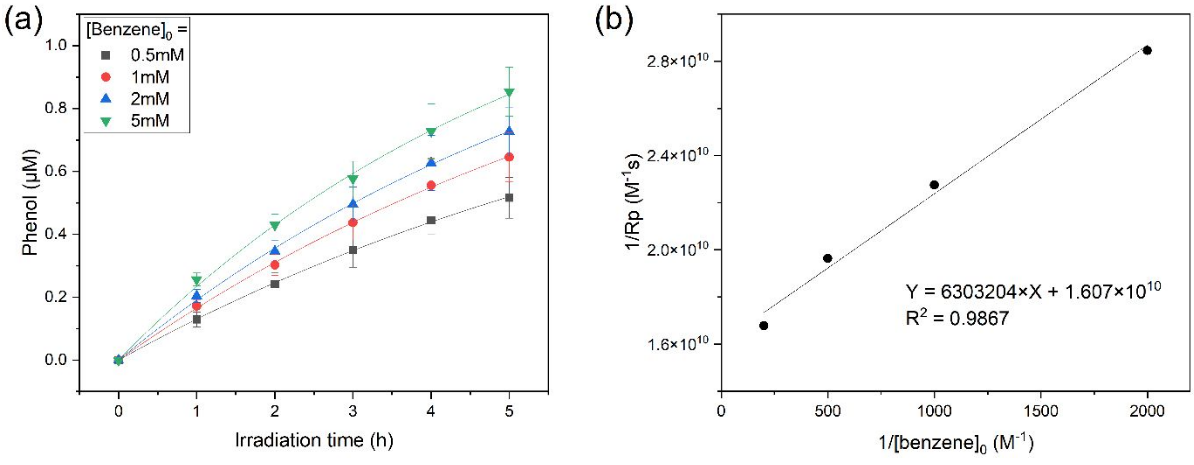

Figure S5. (a) Formation of phenol using different initial concentrations of benzene, and (b)

the plot $1/R_{phenol}$ vs. 1/[benzene] for the C0 samples.

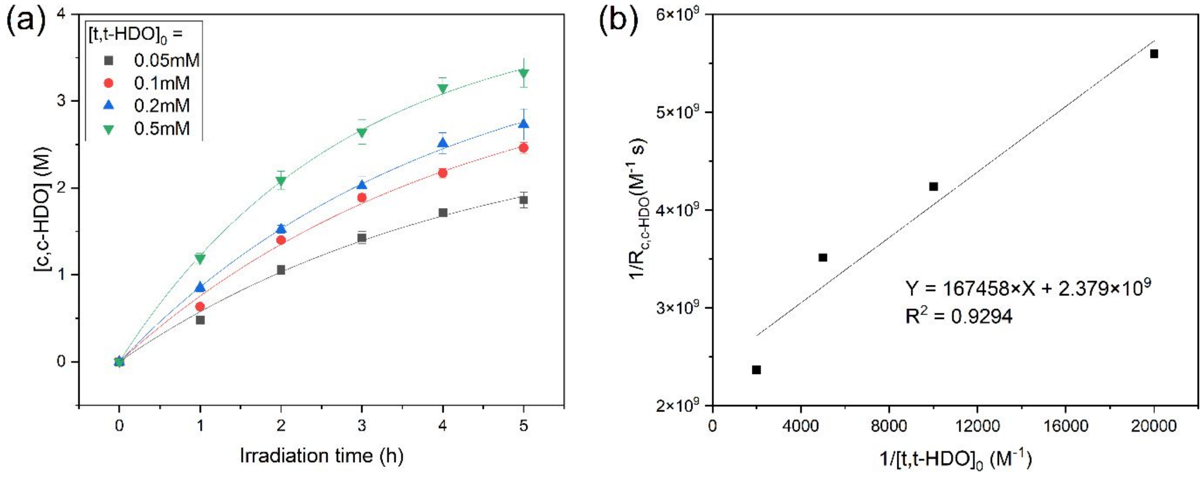

Figure S6. (a) Formation of c, c-HDO with different initial concentrations of t, t-HDO, and (b) the plot $1/R_{c,c\text{-}HDO}$ vs. 1/[t, t-HDO] for the C0 samples.

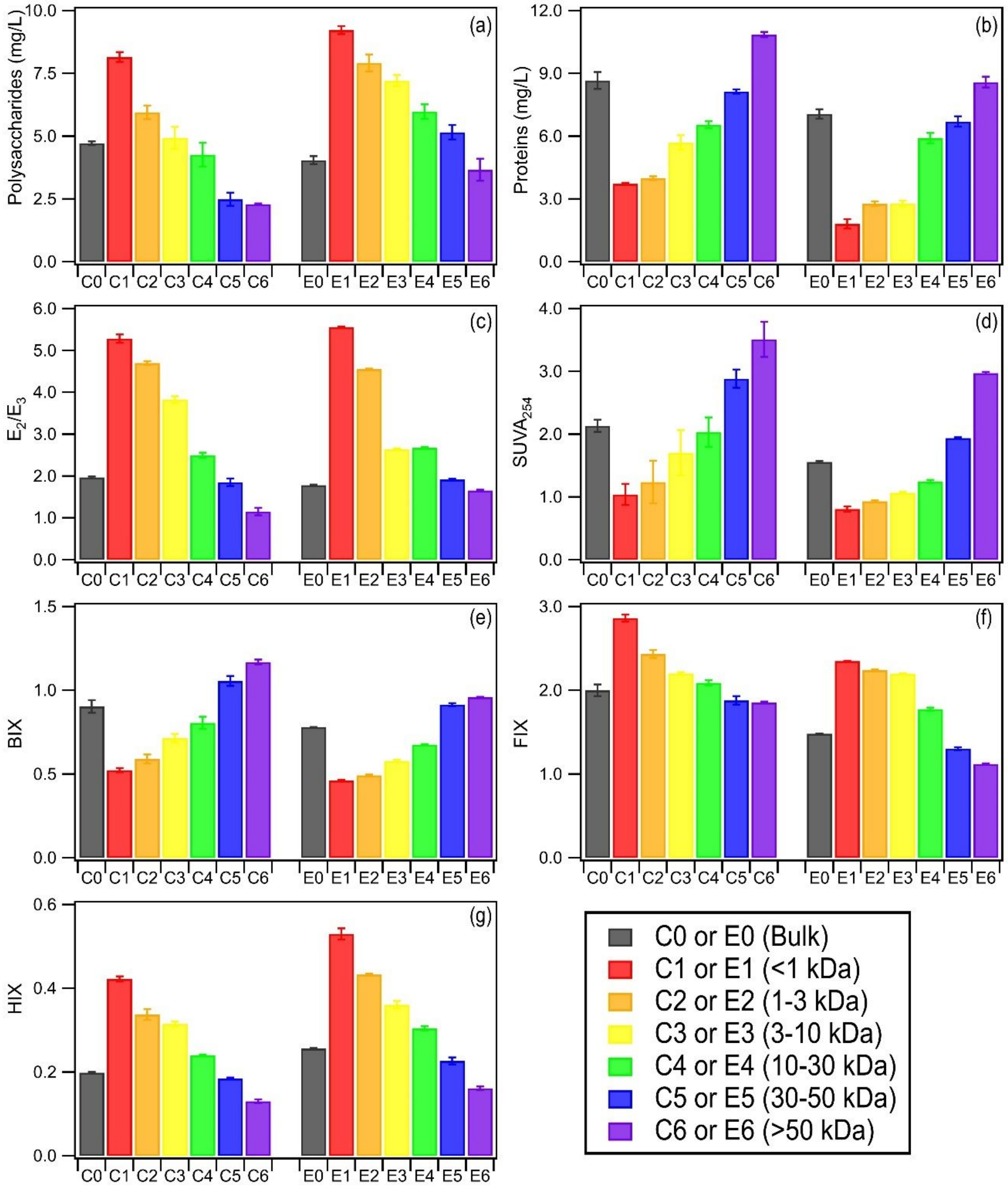

Figure S7. (a) Polysaccharides and (b) protein concentrations, (c) E_2/E_3, (d) $SUVA_{254}$, (e) BIX, (f) FIX, and (g) HIX of bulk and MW-fractionated COM and EPS samples extracted from *B. subtilis* ATCC 6051-U. Error bars denote one standard deviation. The DOC of each fraction was fixed to 5 mg C/L.

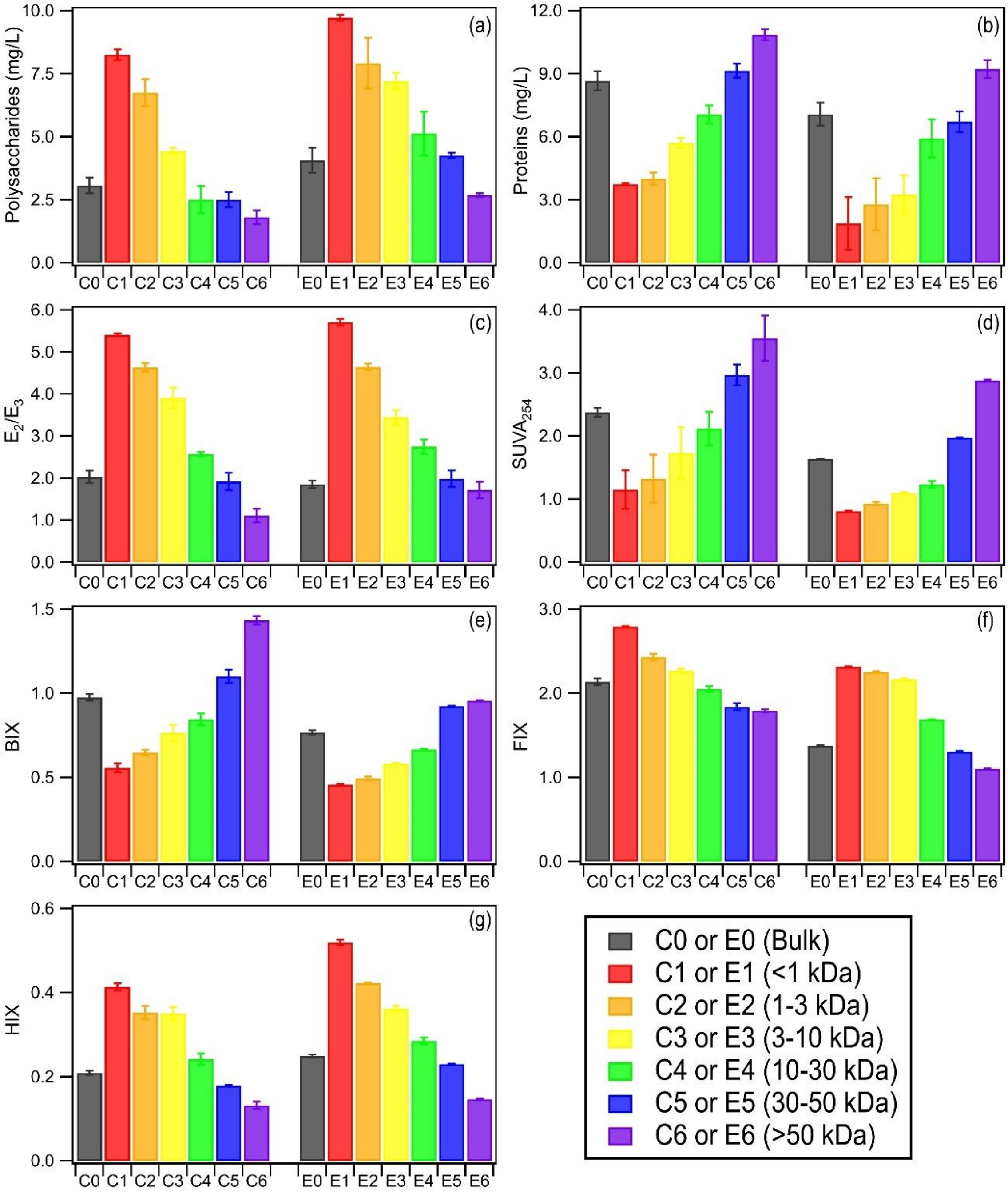

Figure S8. (a) Polysaccharides and (b) protein concentrations, (c) E_2/E_3, (d) $SUVA_{254}$, (e) BIX, (f) FIX, and (g) HIX of bulk and MW-fractionated COM and EPS samples extracted from *P. putida* ATCC 23467. Error bars denote one standard deviation. The DOC of each fraction was fixed to 5 mg C/L.

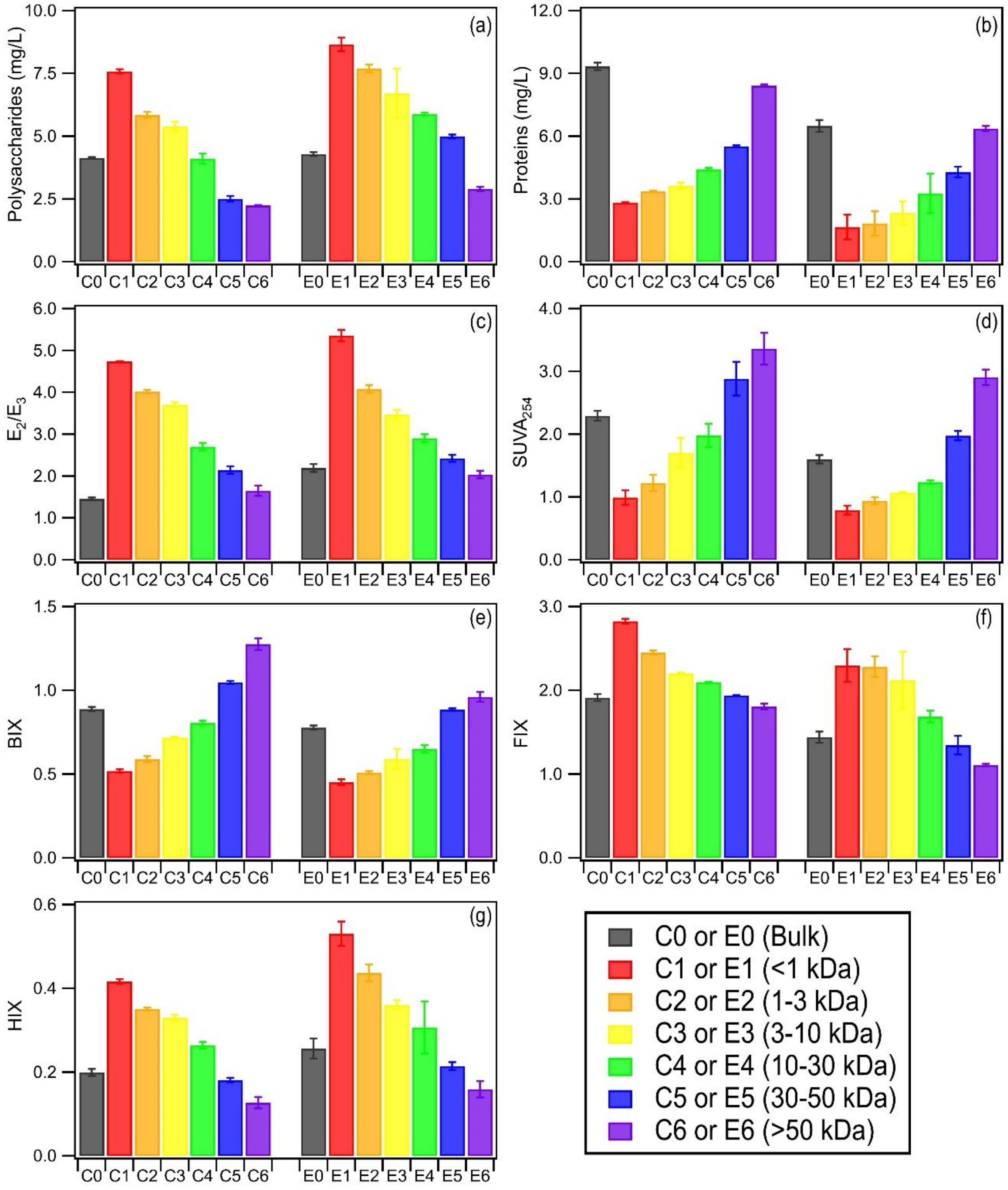

Figure S9. (a) Polysaccharides and (b) protein concentrations, (c) E_2/E_3, (d) $SUVA_{254}$, (e) BIX, (f) FIX, and (g) HIX of bulk and MW-fractionated COM and EPS samples extracted from *E. hormaechei* B0910. Error bars denote one standard deviation. The DOC of each fraction was fixed to 5 mg C/L.

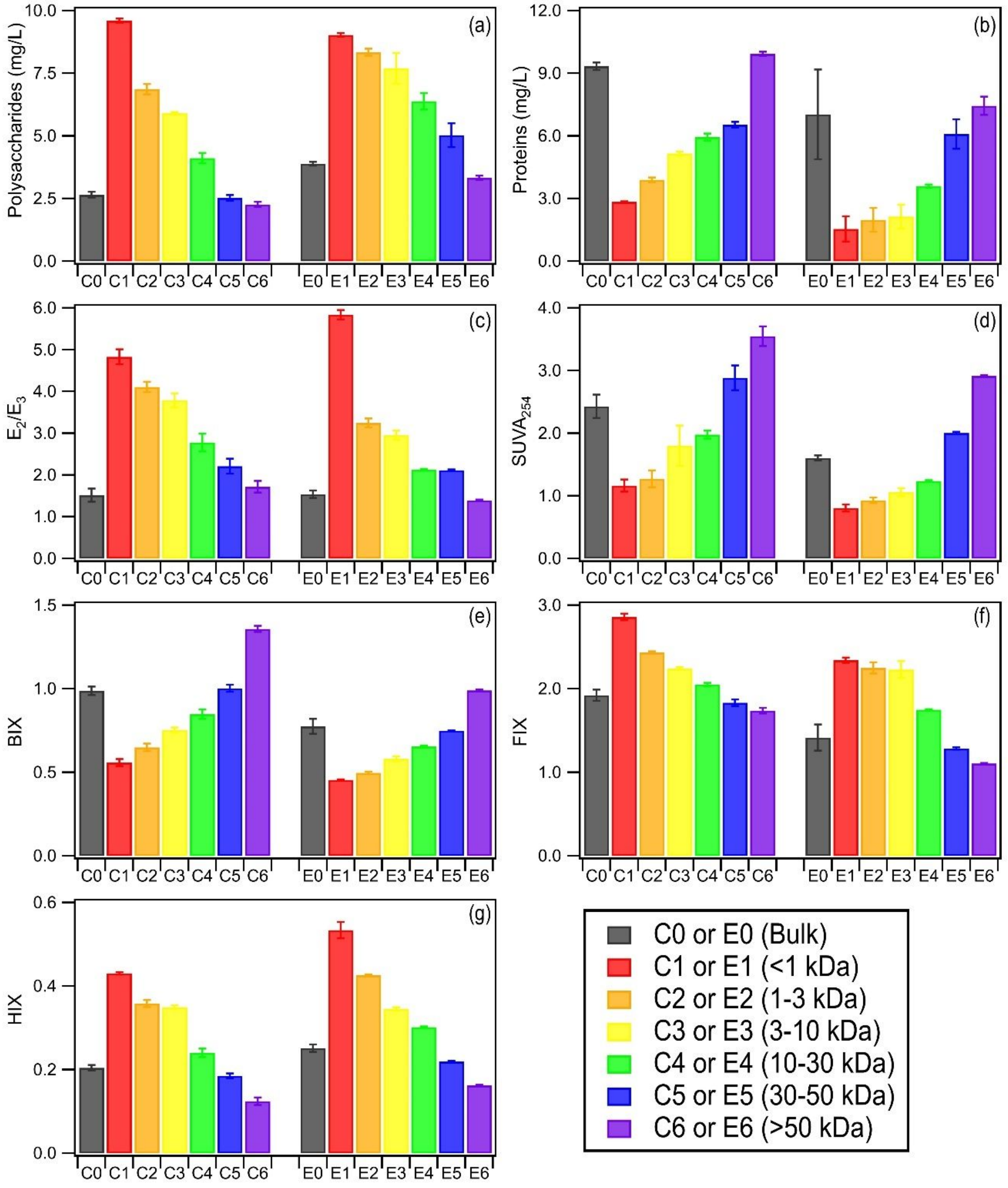

Figure S10. (a) Polysaccharides and (b) protein concentrations, (c) E_2/E_3, (d) $SUVA_{254}$, (e) BIX, (f) FIX, and (g) HIX of bulk and MW-fractionated COM and EPS samples extracted from *E. hormaechei* pf0910. Error bars denote one standard deviation. The DOC of each fraction was fixed to 5 mg C/L.

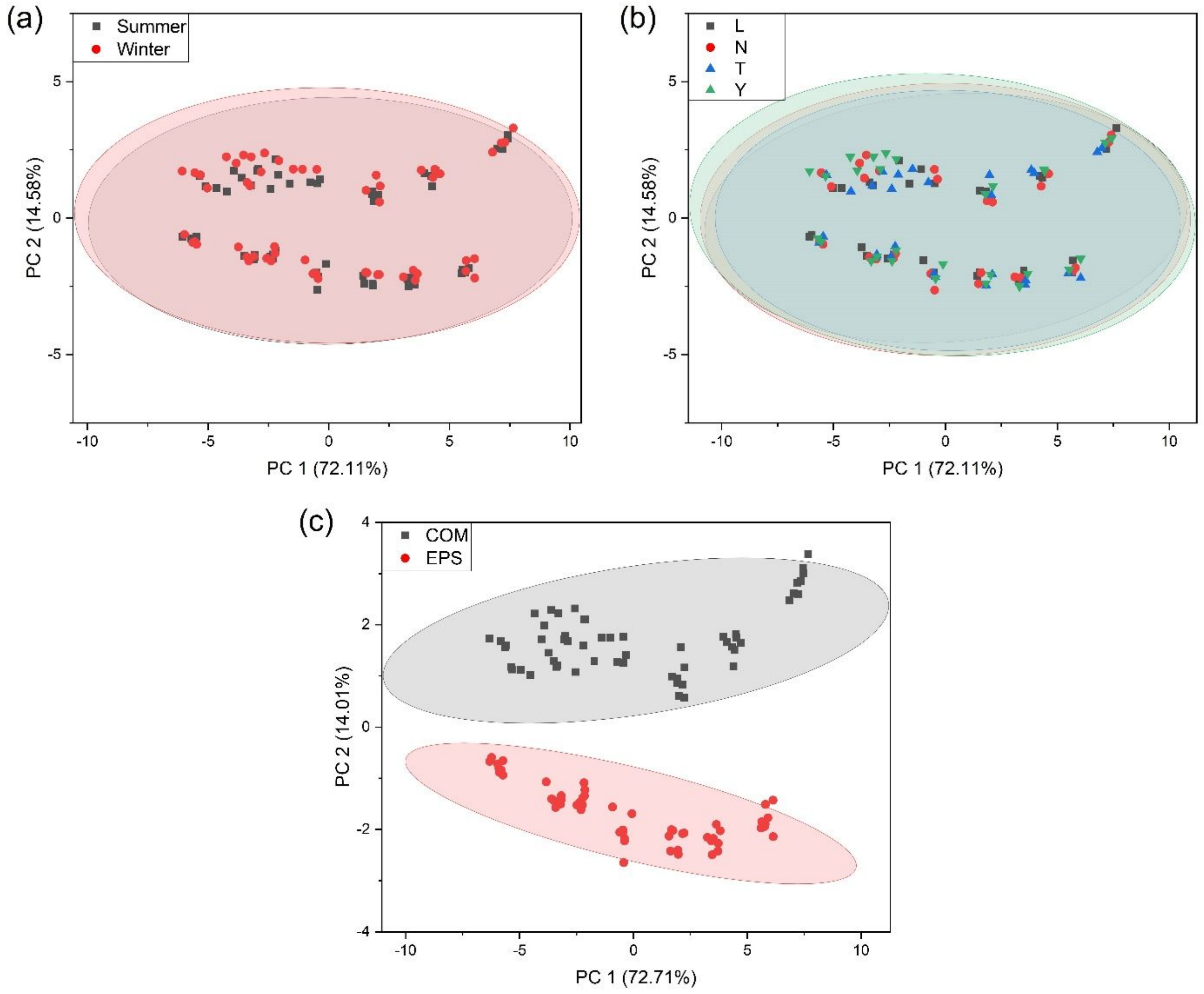

Figure S11. PCA Score plots obtained for Φ_{RI}, $[RI]_{ss}$, protein and polysaccharide concentrations, optical and fluorescence measurements grouped according to (a) summer vs. winter, (b) different culture media, and (c) EPS vs. COM. Comparisons of the Φ_{RI}, $[RI]_{ss}$, protein and polysaccharide concentrations, optical and fluorescence measurements for COM and EPS from culturable bacteria in $PM_{2.5}$ collected in summer vs. winter can be found in Figures S37 to S41.

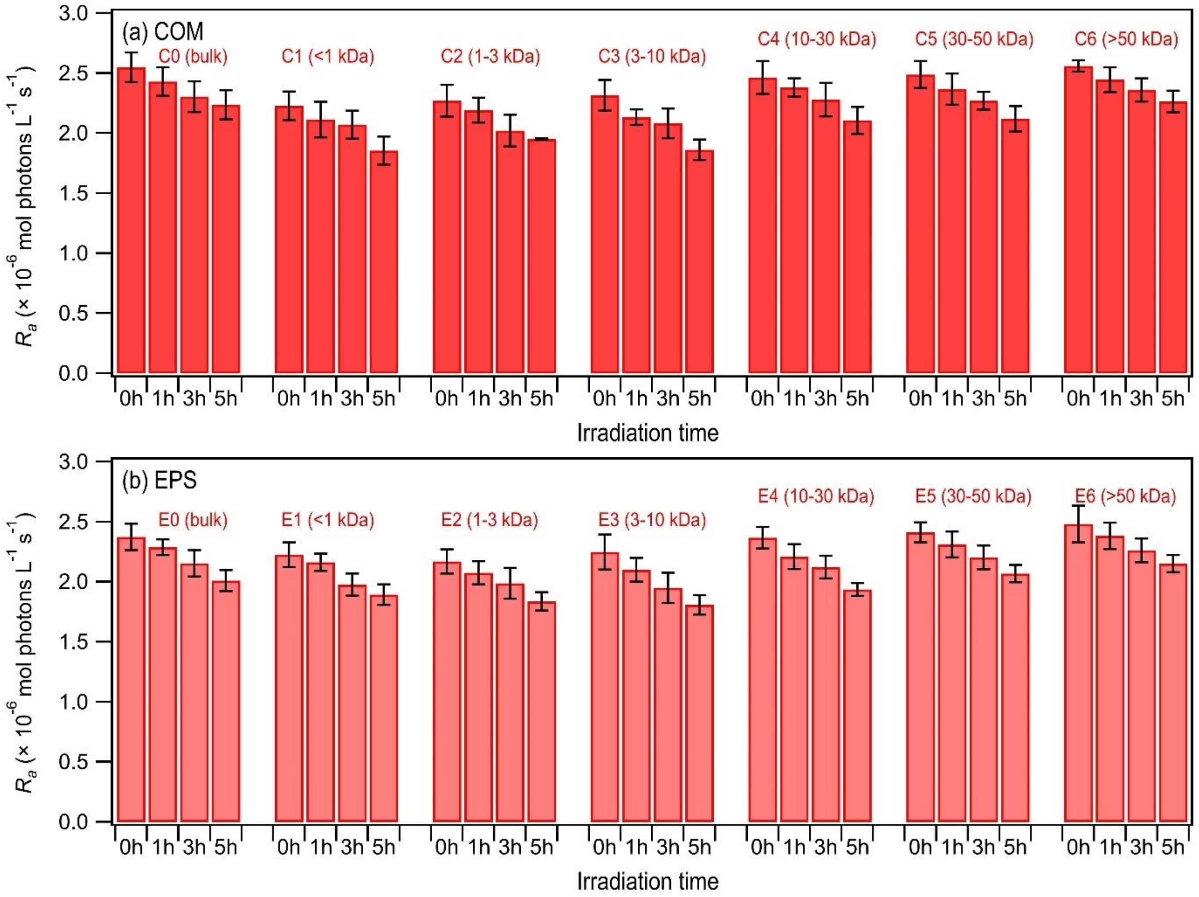

Figure S12. Time evolution of the R_a values for the bulk and MW-fractionated (a) COM and (b) EPS samples from culturable bacteria in $PM_{2.5}$ during irradiation. The DOC of each fraction was fixed to 5 mg C/L before irradiation.

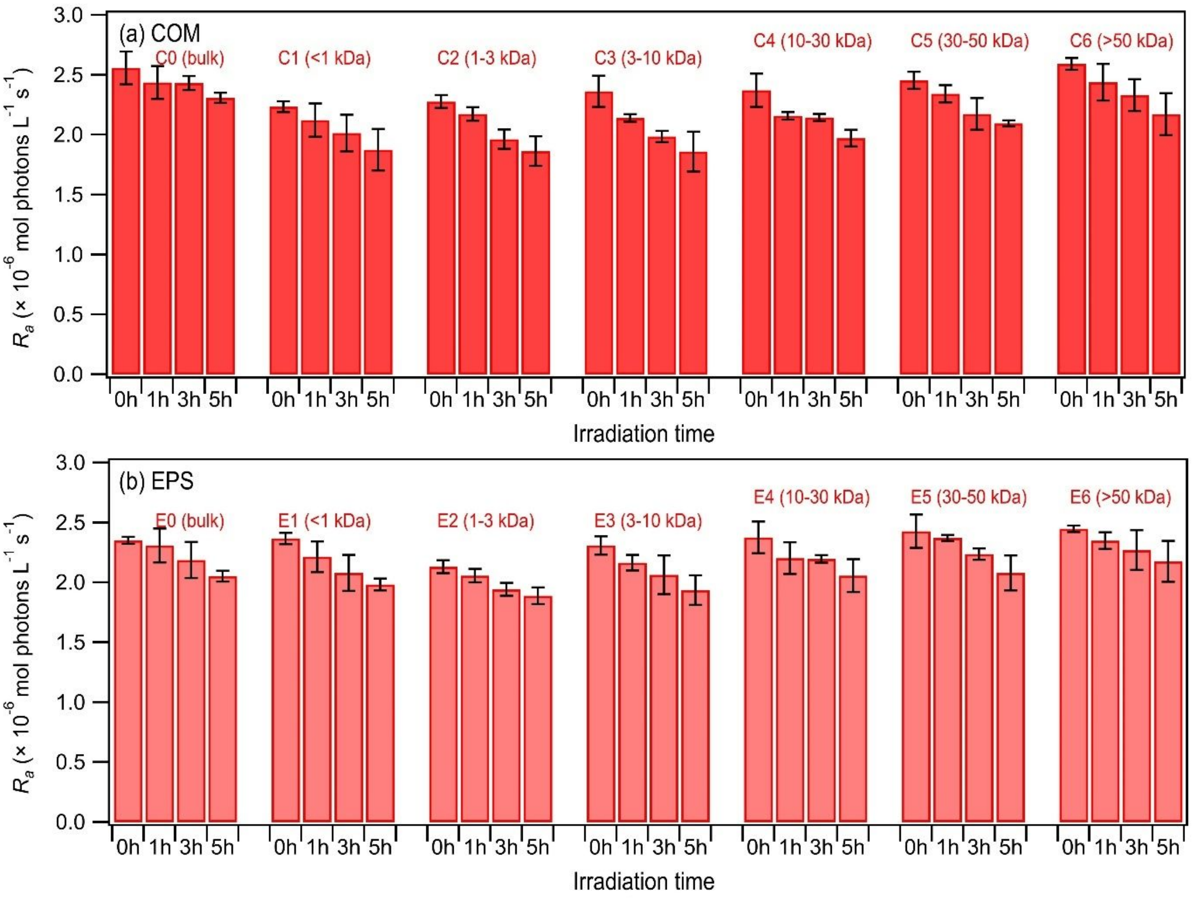

Figure S13. Time evolution of the R_a values for the bulk and MW-fractionated (a) COM and (b) EPS samples extracted from *B. subtilis* ATCC 6051-U during irradiation. The DOC of each fraction was fixed to 5 mg C/L before irradiation.

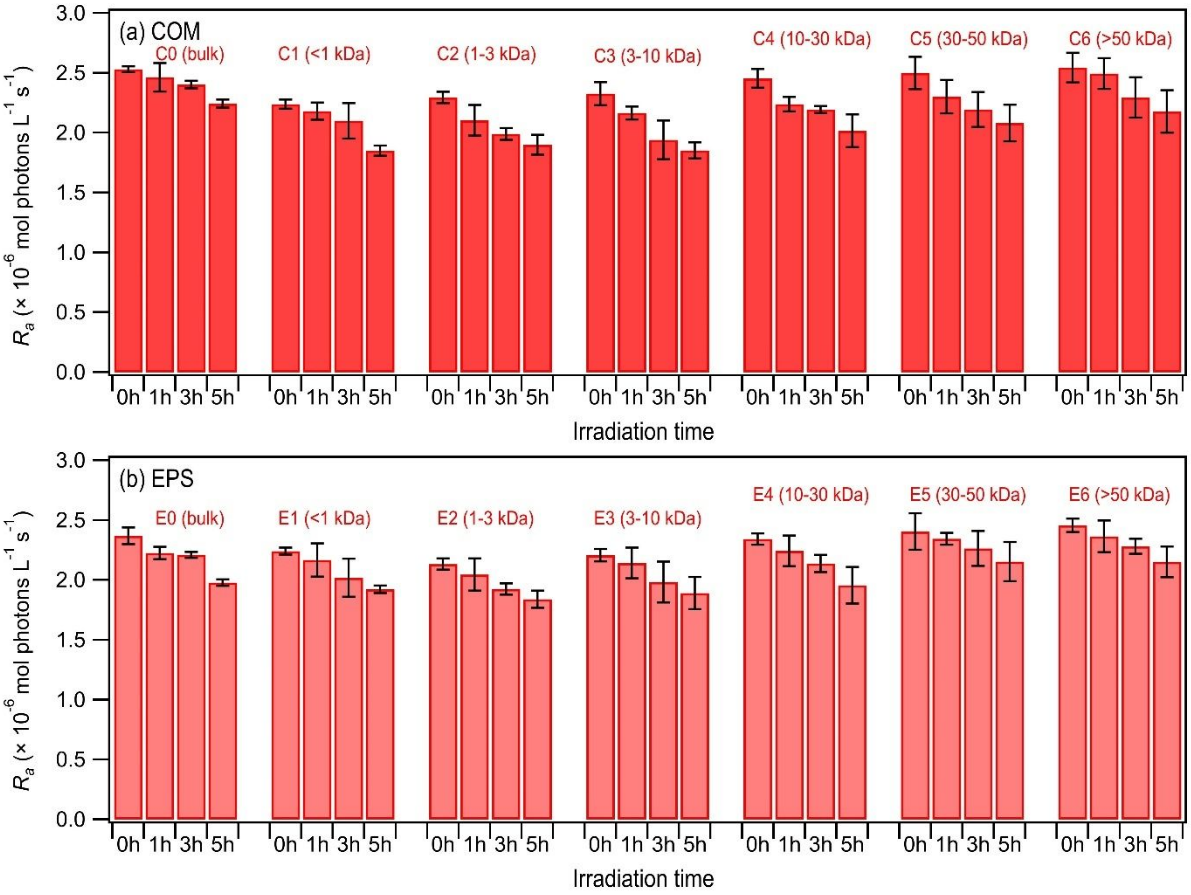

Figure S14. Time evolution of the R_a values for the bulk and MW-fractionated (a) COM and (b) EPS samples extracted from *P. putida* ATCC 23467 during irradiation. The DOC of each fraction was fixed to 5 mg C/L before irradiation.

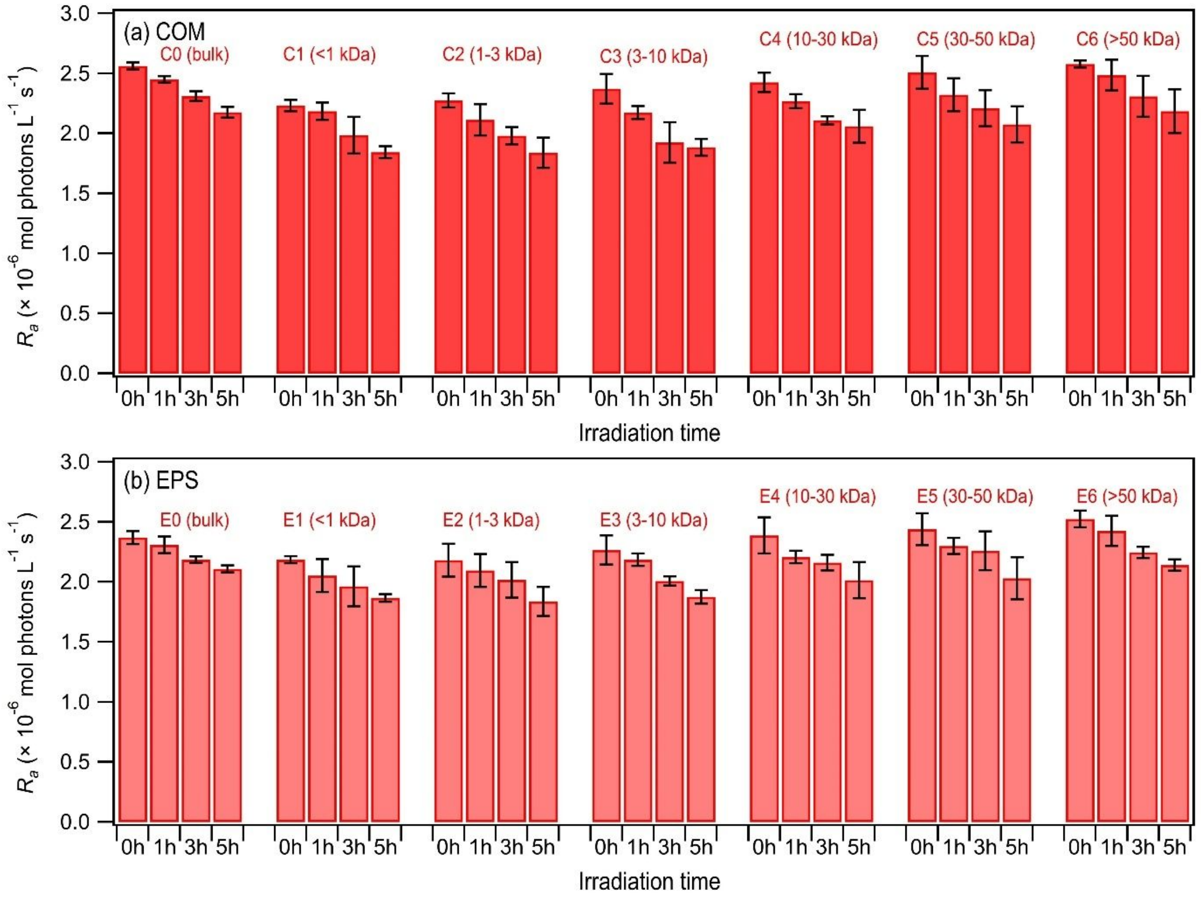

Figure S15. Time evolution of the R_a values for the bulk and MW-fractionated (a) COM and (b) EPS samples extracted from *E. hormaechei* B0910 during irradiation. The DOC of each fraction was fixed to 5 mg C/L before irradiation.

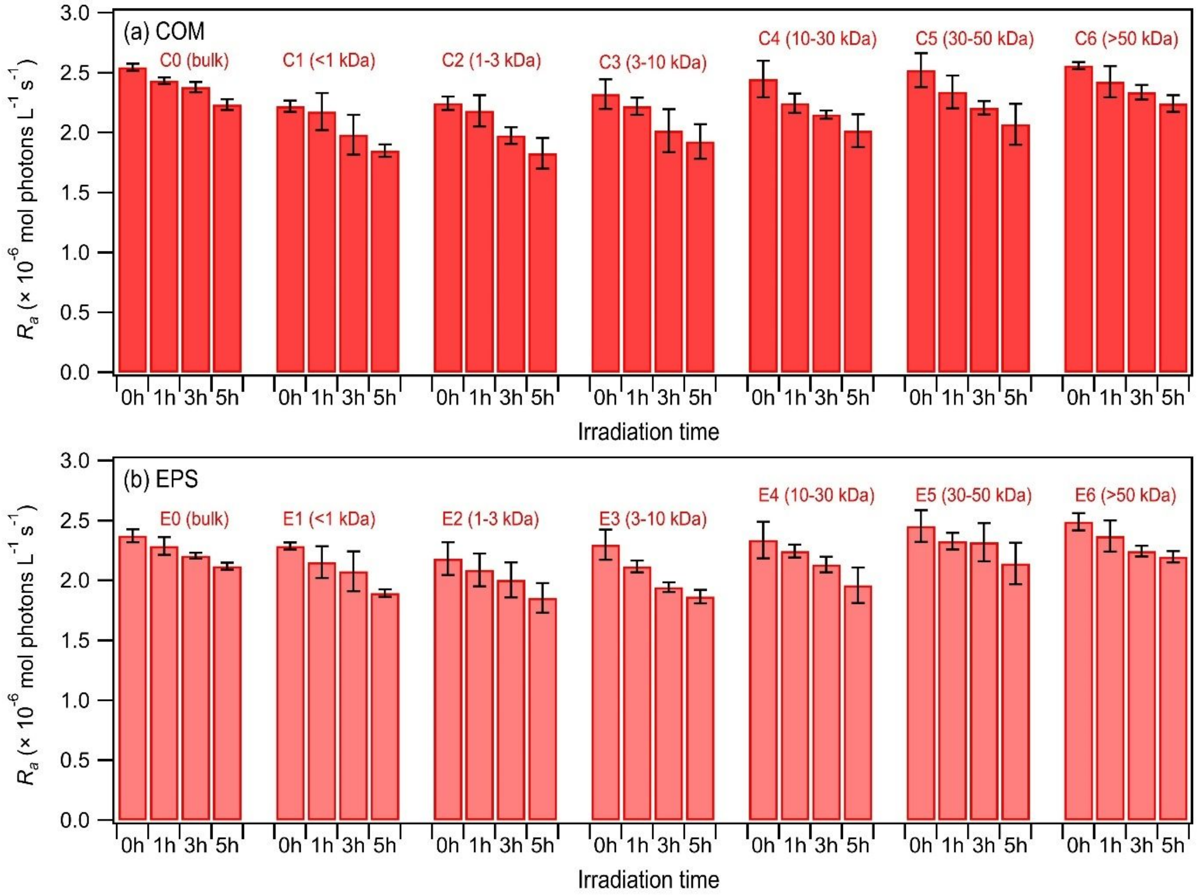

Figure S16. Time evolution of the R_a values for the bulk and MW-fractionated (a) COM and (b) EPS samples extracted from *E. hormaechei* pf0910 during irradiation. The DOC of each fraction was fixed to 5 mg C/L before irradiation.

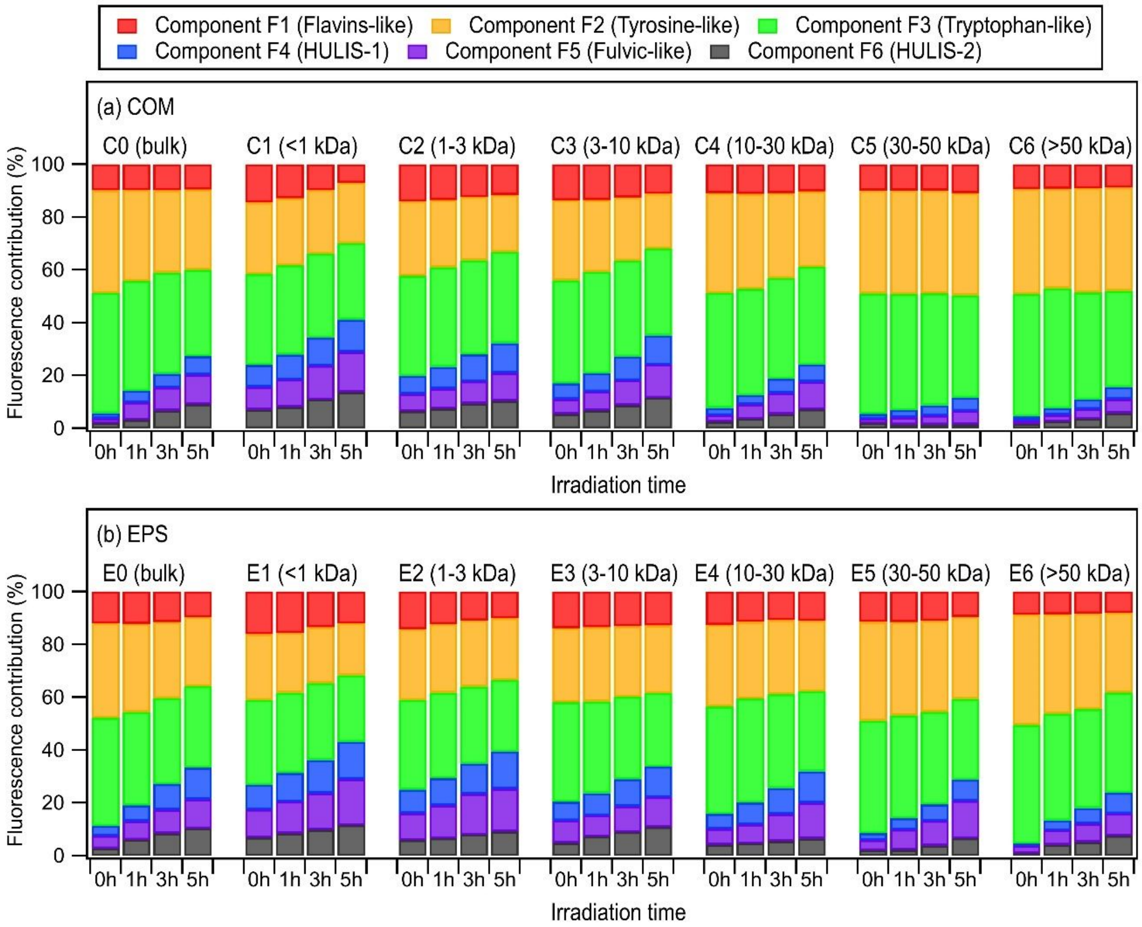

Figure S17. Time evolution of the relative contributions of the six PARAFAC-extracted components for bulk and MW-fractionated (a) COM and (b) EPS samples from culturable bacteria in $PM_{2.5}$ during irradiation. The DOC of each fraction was fixed to 5 mg C/L before irradiation.

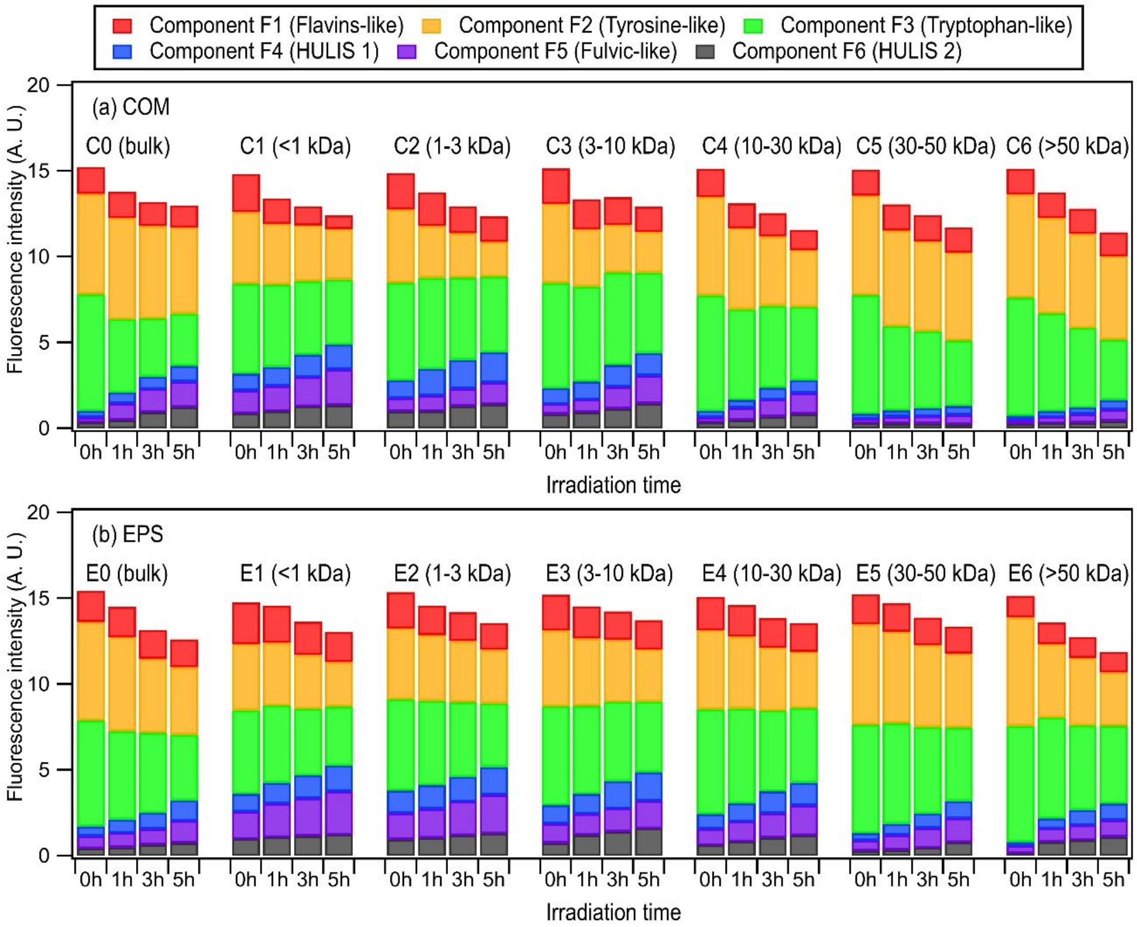

Figure S18. Time evolution of the six PARAFAC-extracted components for bulk and MW-fractionated (a) COM and (b) EPS samples extracted from *B. subtilis* ATCC 6051-U during irradiation. The DOC of each fraction was fixed to 5 mg C/L before irradiation.

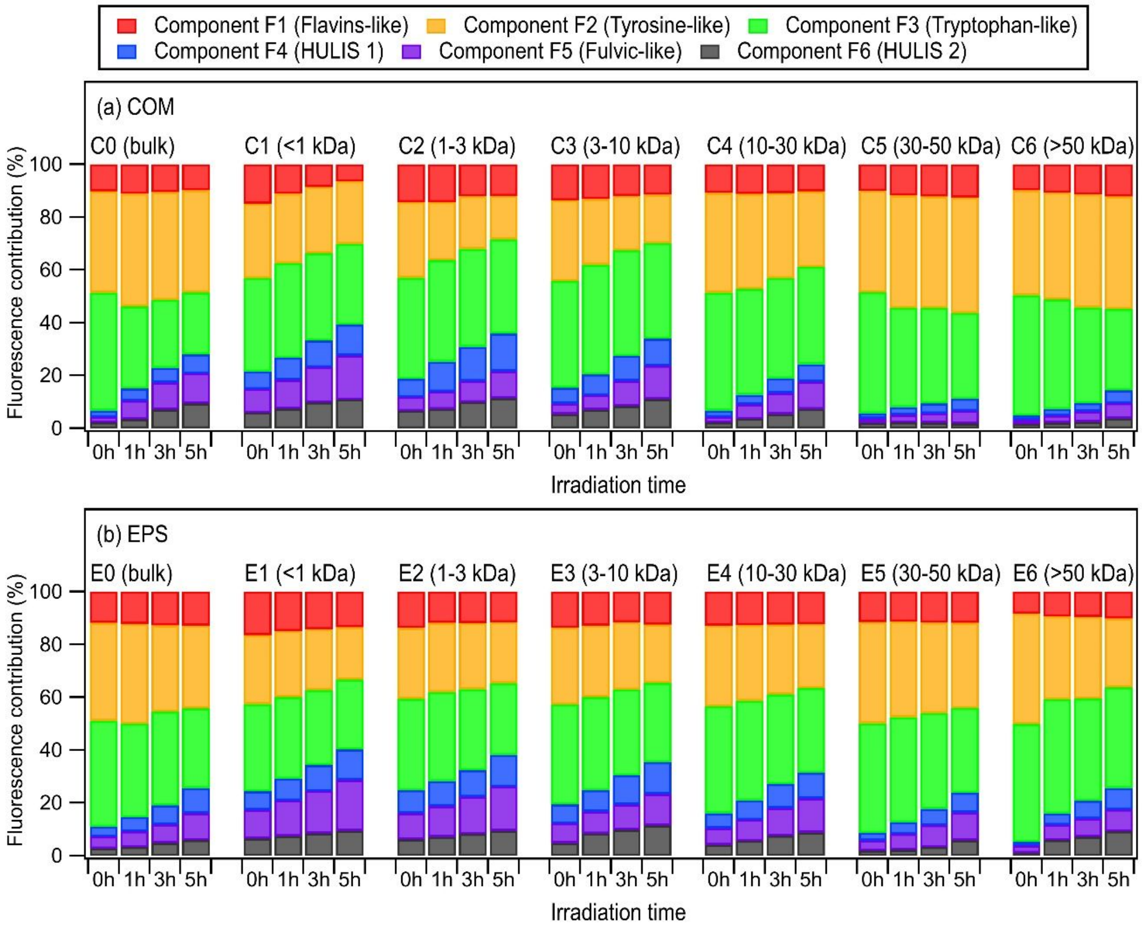

Figure S19. Time evolution of the relative contributions of the six PARAFAC-extracted components for bulk and MW-fractionated (a) COM and (b) EPS samples extracted from *B. subtilis* ATCC 6051-U during irradiation. The DOC of each fraction was fixed to 5 mg C/L before irradiation.

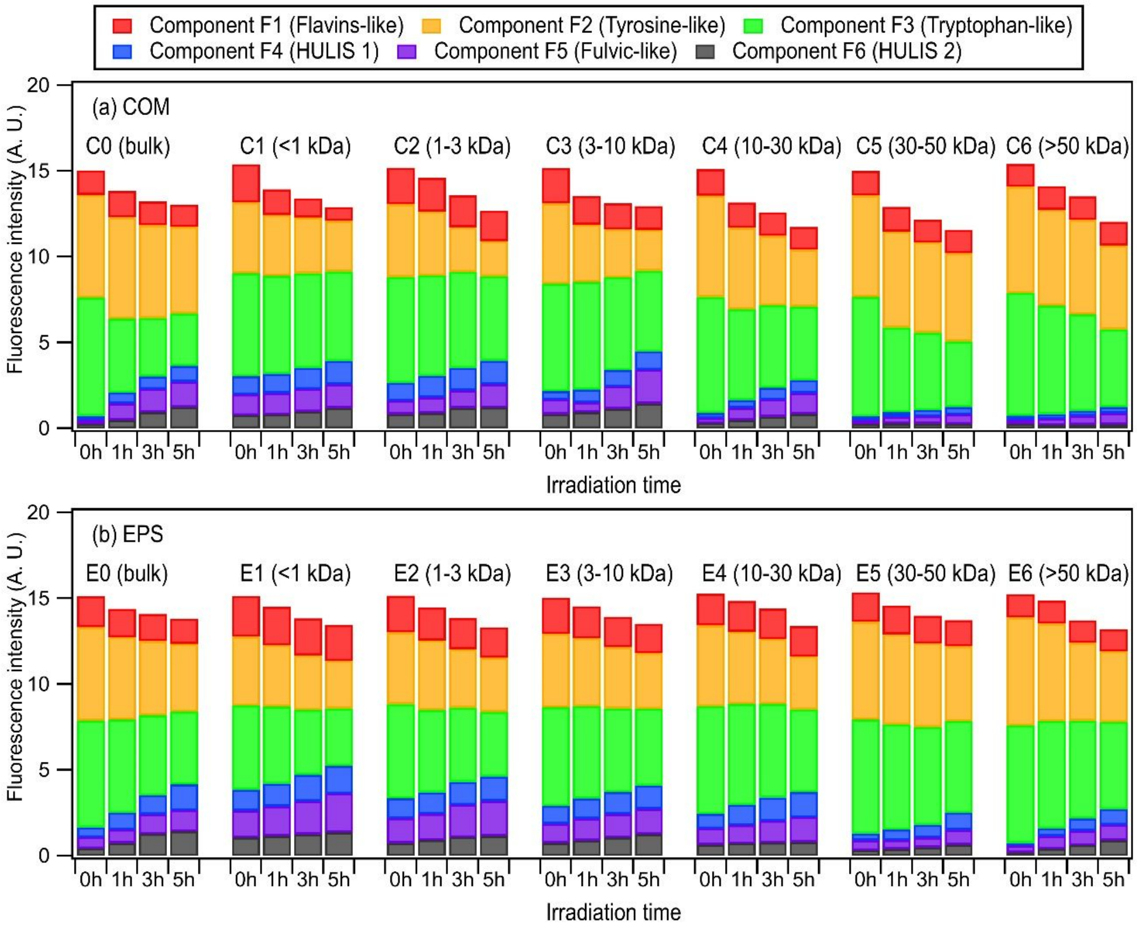

Figure S20. Time evolution of the six PARAFAC-extracted components for bulk and MW-fractionated (a) COM and (b) EPS samples extracted from *P. putida* ATCC 23467 during irradiation. The DOC of each fraction was fixed to 5 mg C/L before irradiation.

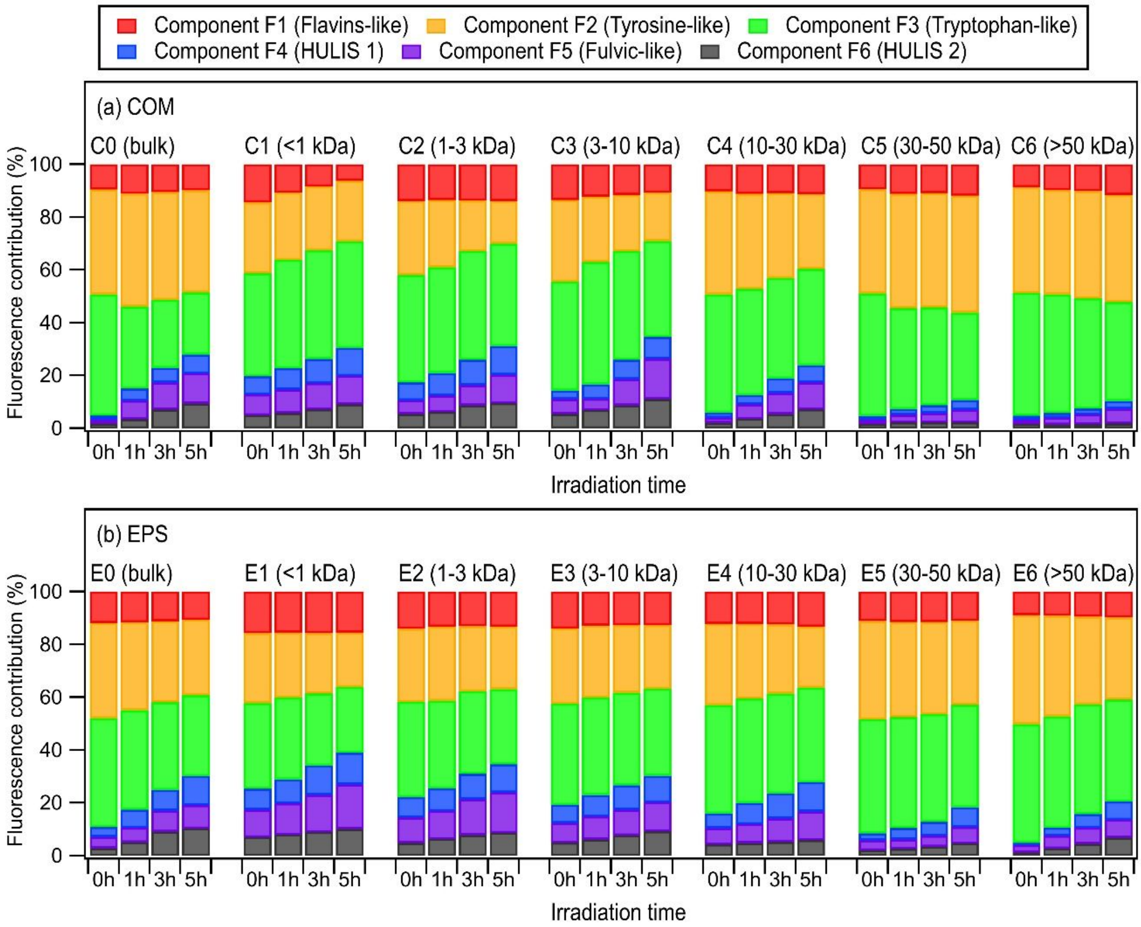

Figure S21. Time evolution of the relative contributions of the six PARAFAC-extracted components for bulk and MW-fractionated (a) COM and (b) EPS samples extracted from *P. putida* ATCC 23467 during irradiation. The DOC of each fraction was fixed to 5 mg C/L before irradiation.

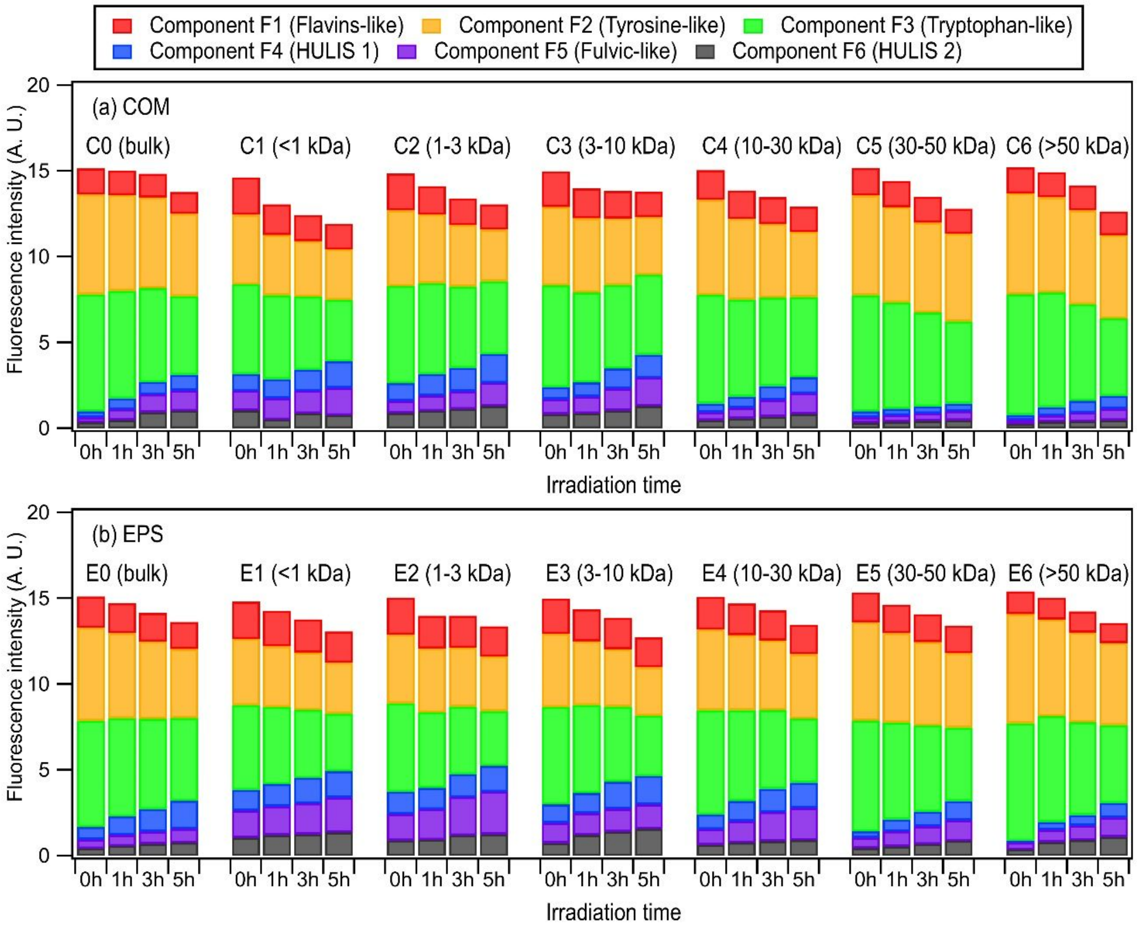

Figure S22. Time evolution of the six PARAFAC-extracted components for bulk and MW-fractionated (a) COM and (b) EPS samples extracted from *E. hormaechei* B0910 during irradiation. The DOC of each fraction was fixed to 5 mg C/L before irradiation.

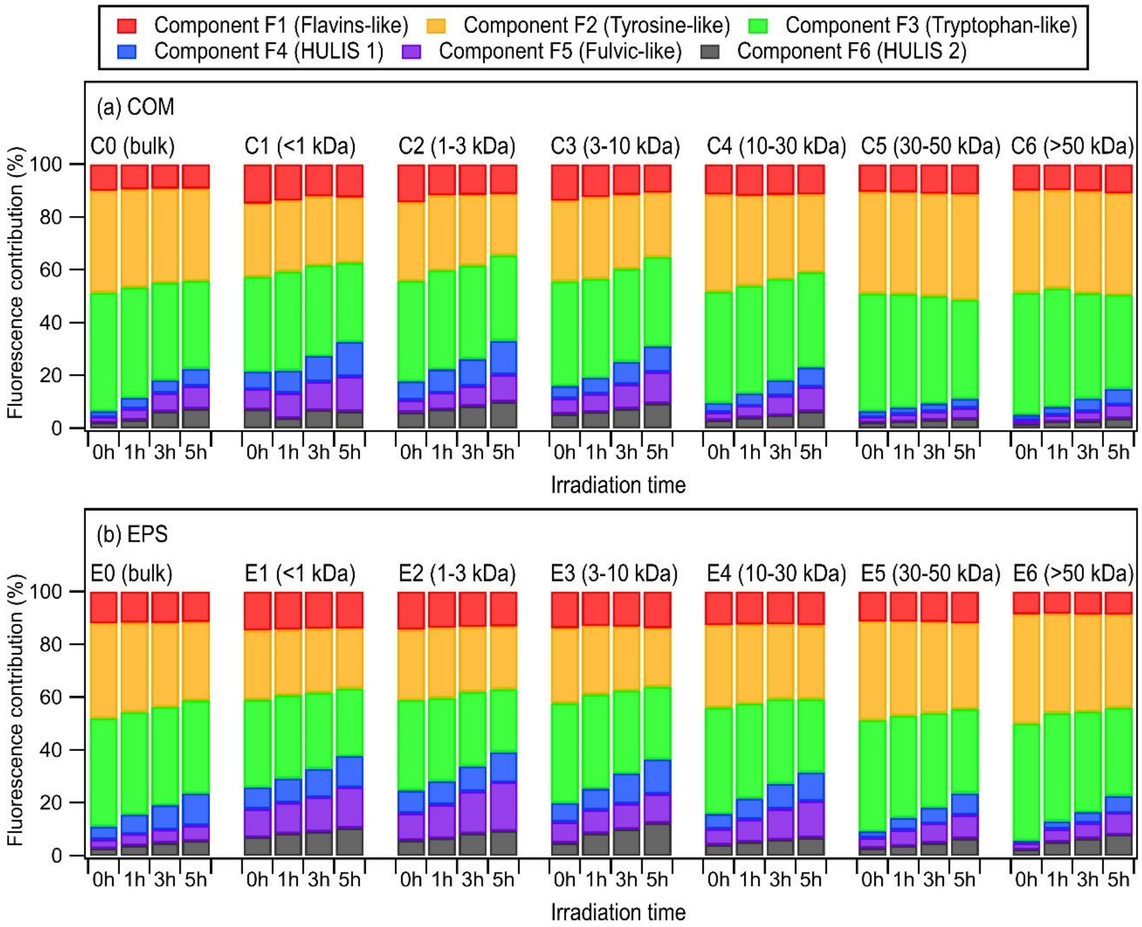

Figure S23. Time evolution of the relative contributions of the six PARAFAC-extracted components for bulk and MW-fractionated (a) COM and (b) EPS samples extracted from *E. hormaechei* B0910 during irradiation. The DOC of each fraction was fixed to 5 mg C/L before irradiation.

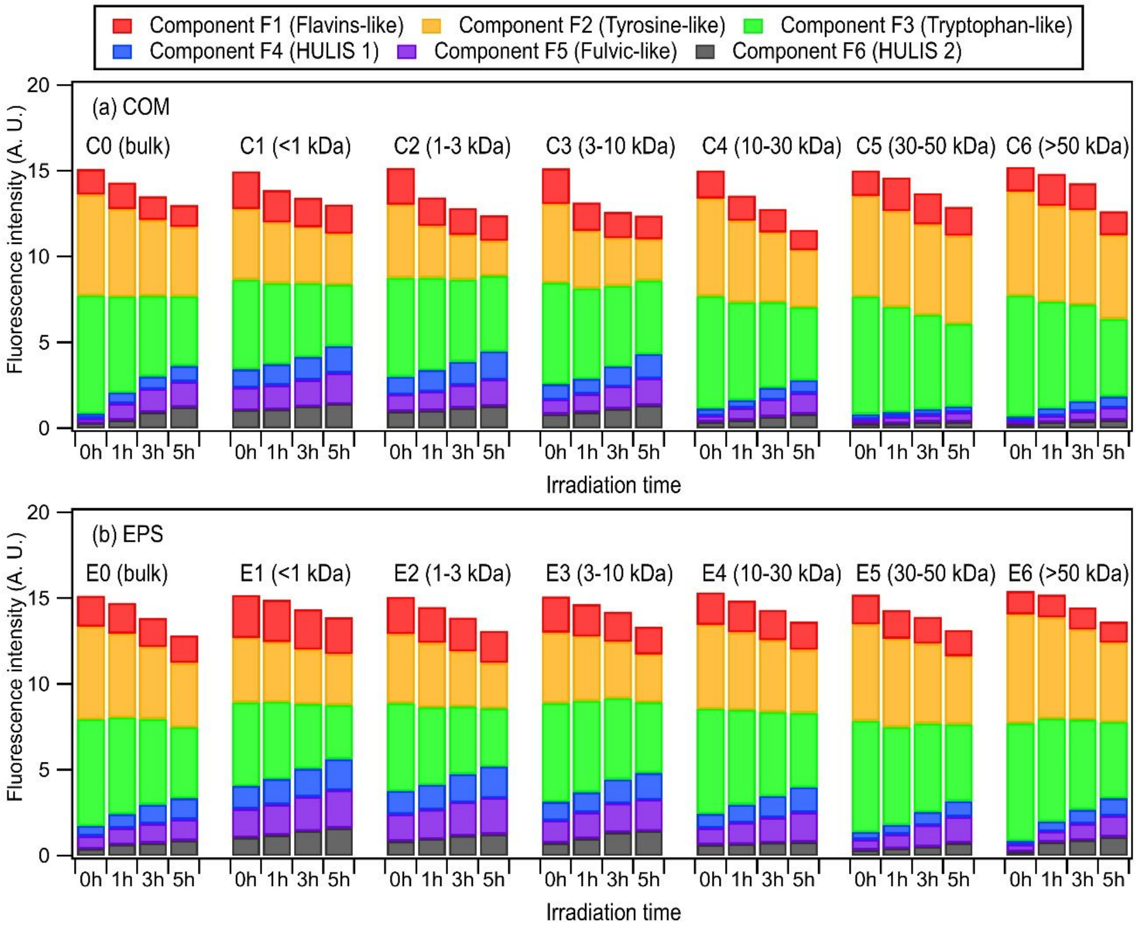

Figure S24. Time evolution of the six PARAFAC-extracted components for bulk and MW-fractionated (a) COM and (b) EPS samples extracted from *E. hormaechei* pf0910 during irradiation. The DOC of each fraction was fixed to 5 mg C/L before irradiation.

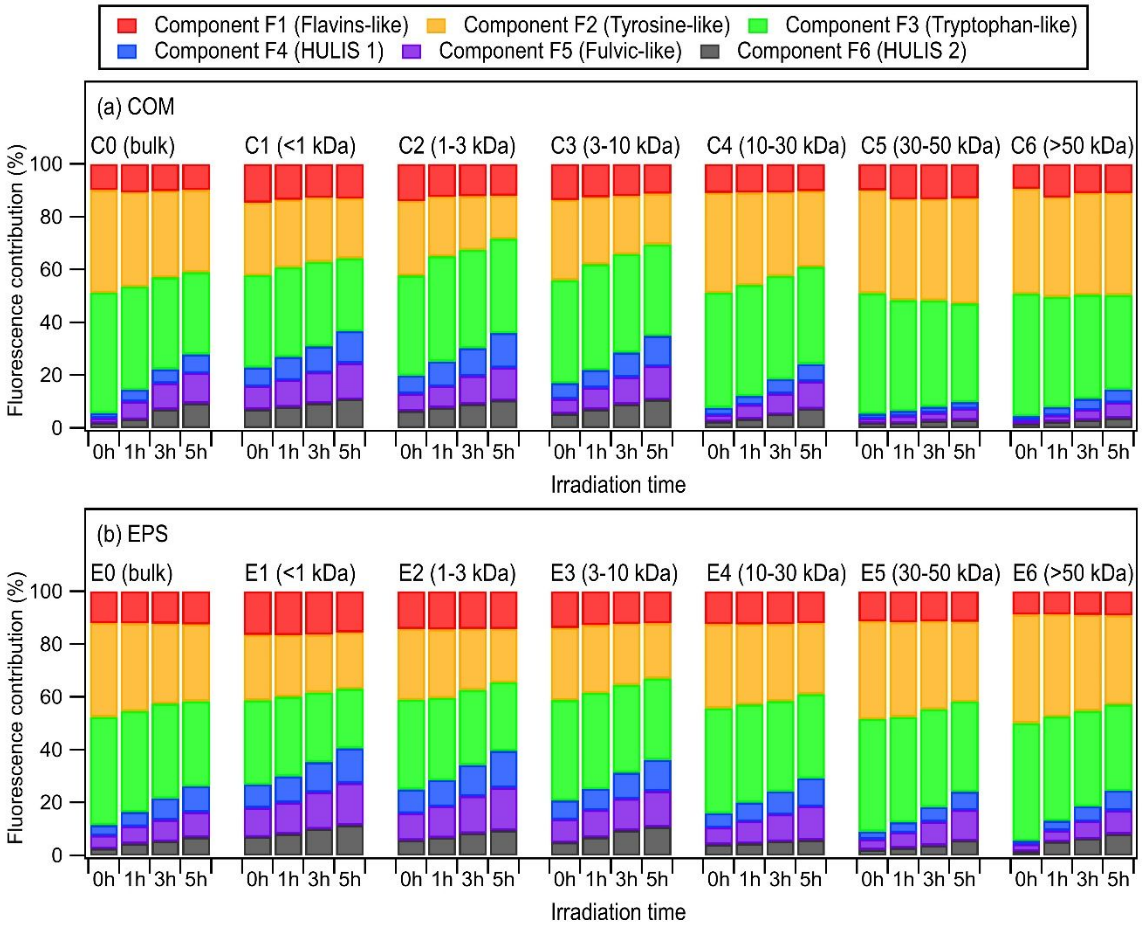

Figure S25. Time evolution of the relative contributions of the six PARAFAC-extracted components for bulk and MW-fractionated (a) COM and (b) EPS samples extracted from *E. hormaechei* pf0910 during irradiation. The DOC of each fraction was fixed to 5 mg C/L before irradiation.

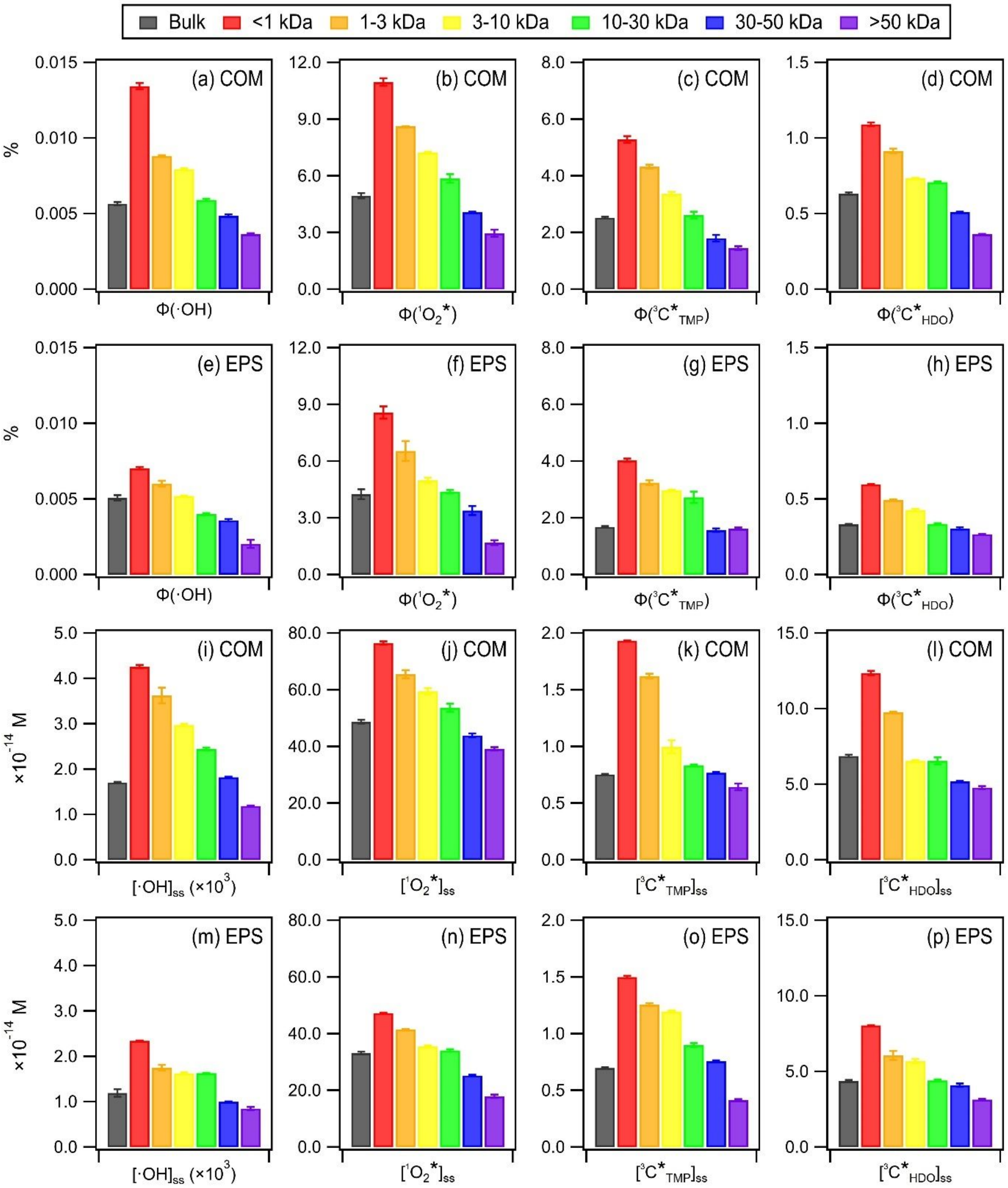

370

371 **Figure S26.** (a to h) Φ_{RI} and, (i to p) $[RI]_{ss}$ of $\cdot OH$, $^1O_2^*$, and $^3C^*$ for bulk and MW-fractionated
372 COM and EPS samples extracted from *B. subtilis* ATCC 6051-U. Error bars denote one
373 standard deviation. The DOC of each fraction was fixed to 5 mg C/L before irradiation.

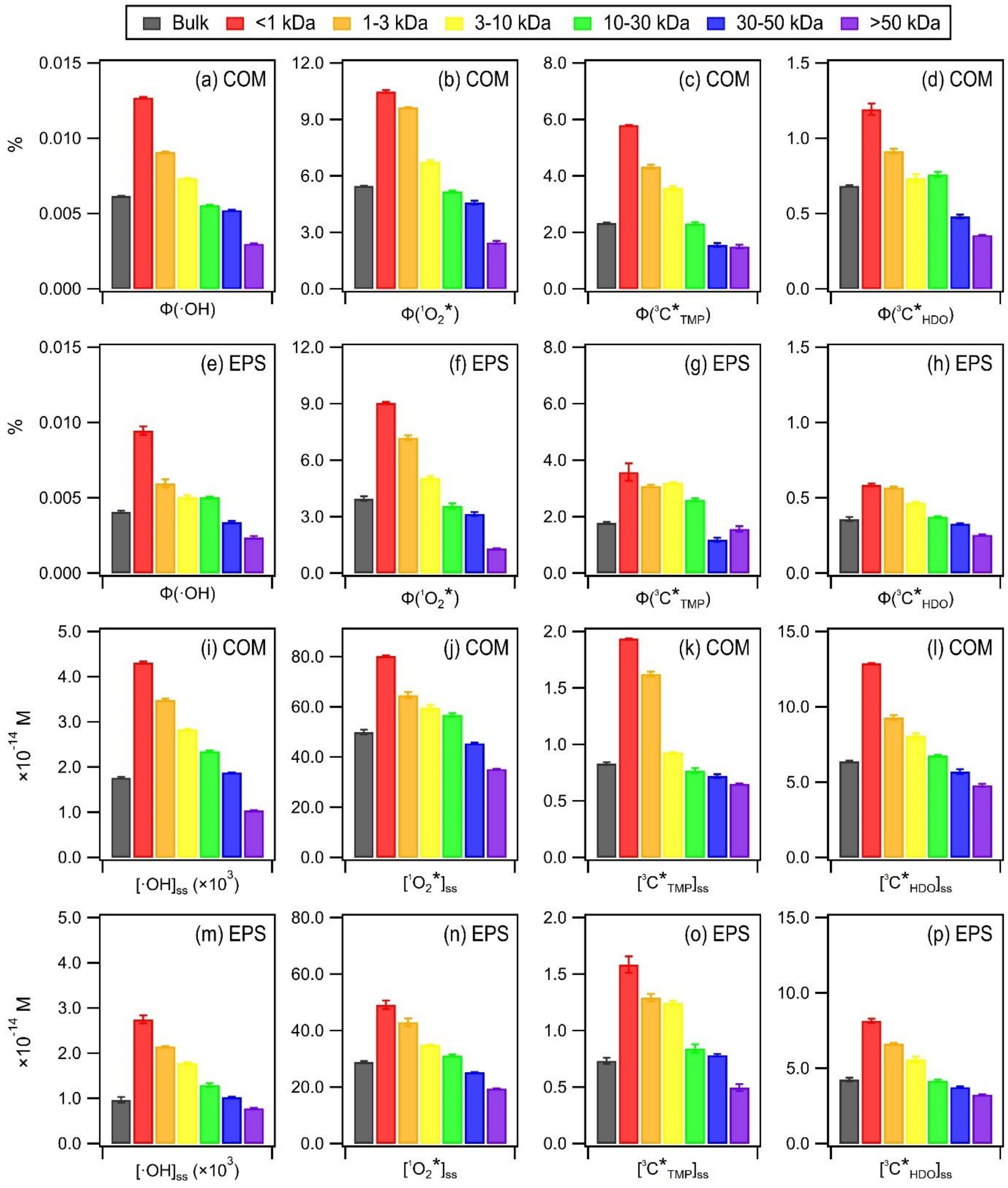

Figure S27. (a to h) Φ_{RI} and, (i to p) $[RI]_{ss}$ of $\cdot OH$, $^1O_2^*$, and $^3C^*$ for bulk and MW-fractionated COM and EPS samples extracted from *P. putida* ATCC 23467. Error bars denote one standard deviation. The DOC of each fraction was fixed to 5 mg C/L before irradiation.

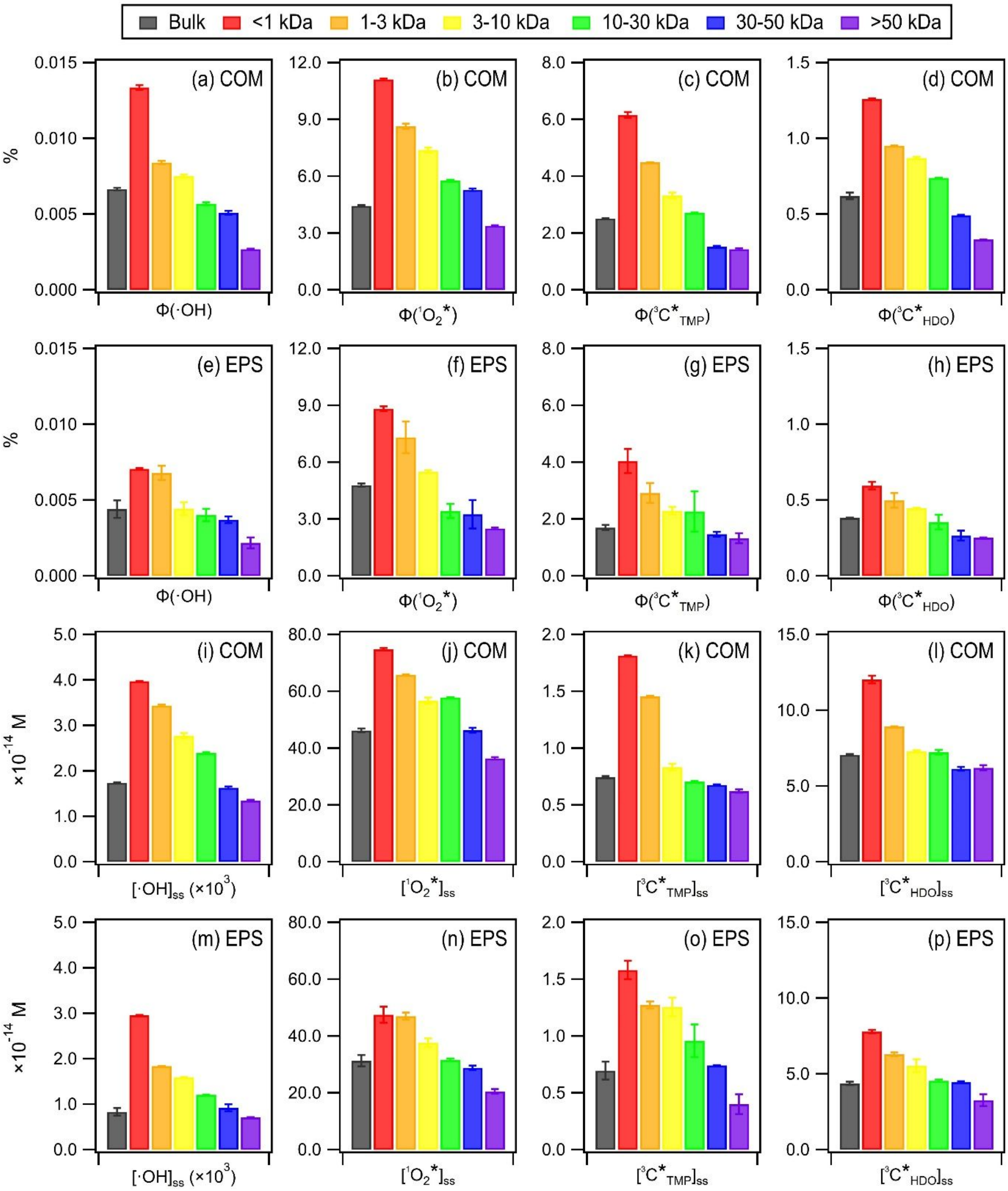

Figure S28. (a to h) Φ_{RI} and, (i to p) $[RI]_{ss}$ of $\cdot OH$, $^1O_2^*$, and $^3C^*$ for bulk and MW-fractionated COM and EPS samples extracted from *E. hormaechei* B0910. Error bars denote one standard deviation. The DOC of each fraction was fixed to 5 mg C/L before irradiation.

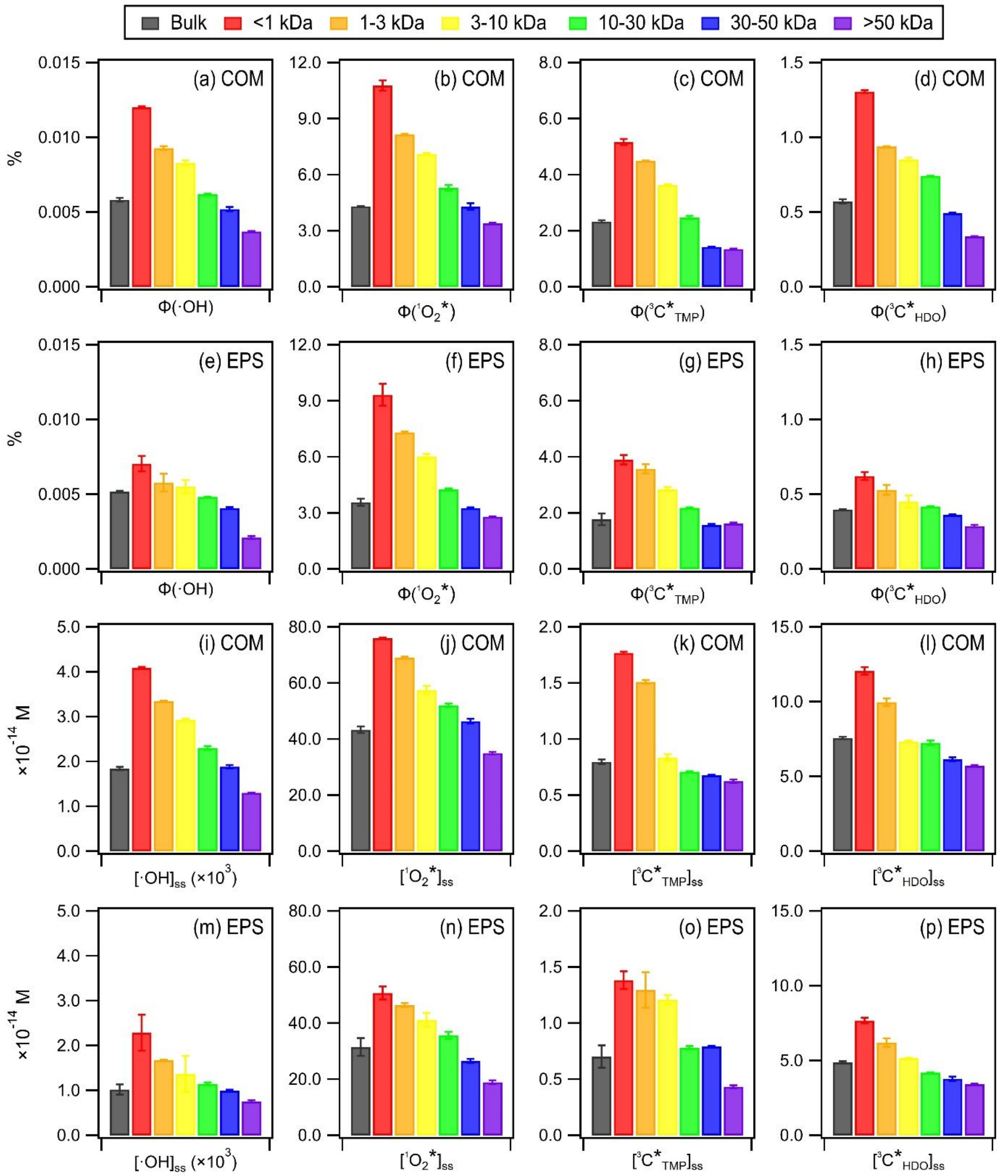

Figure S29. (a to h) Φ_{RI} and, (i to p) $[RI]_{ss}$ of $\cdot OH$, $^1O_2^*$, and $^3C^*$ for bulk and MW-fractionated COM and EPS samples extracted from *E. hormaechei* pf0910. Error bars denote one standard deviation. The DOC of each fraction was fixed to 5 mg C/L before irradiation.

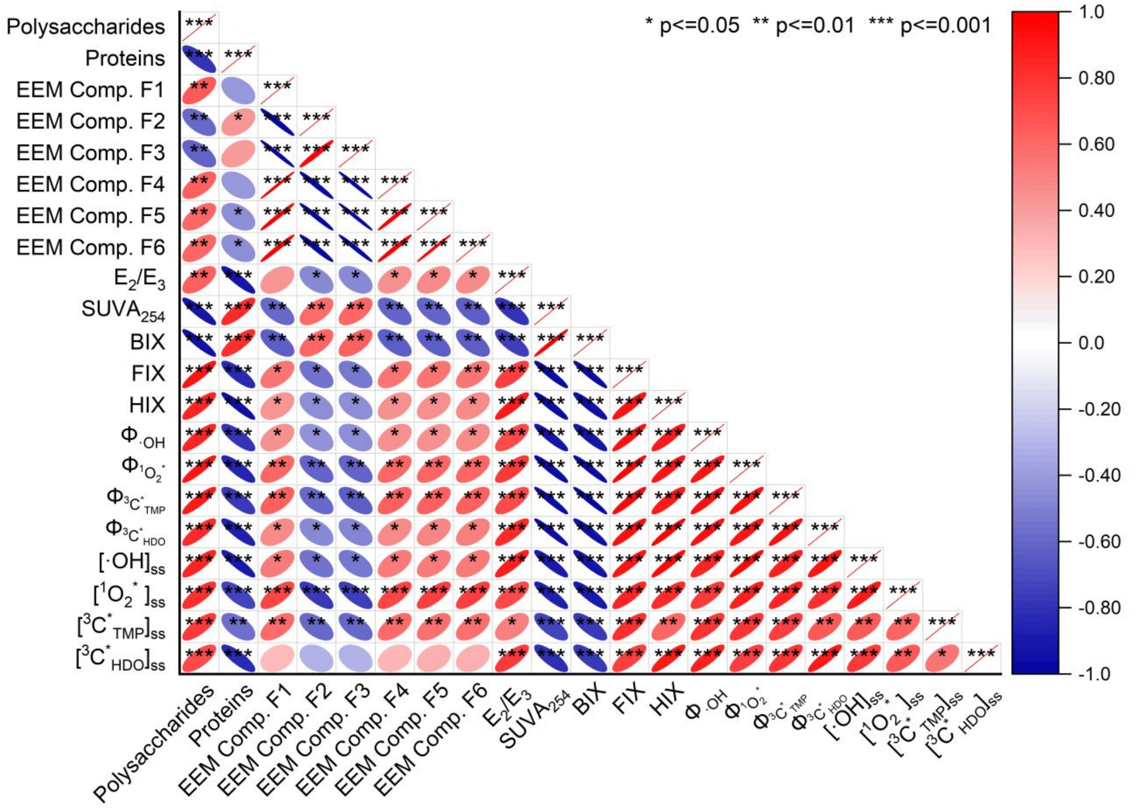

Figure S30. Correlation matrix between Φ_{RI}, $[RI]_{ss}$, protein and polysaccharide concentrations, optical and fluorescence parameters compiled from the bulk and MW-fractionated COM samples extracted from culturable bacteria in $PM_{2.5}$ ($p < 0.05$, *; $p < 0.01$, **; $p < 0.001$, ***). The color and size of the squares indicate the correlation coefficients. Red and blue indicate positive and negative correlations, respectively.

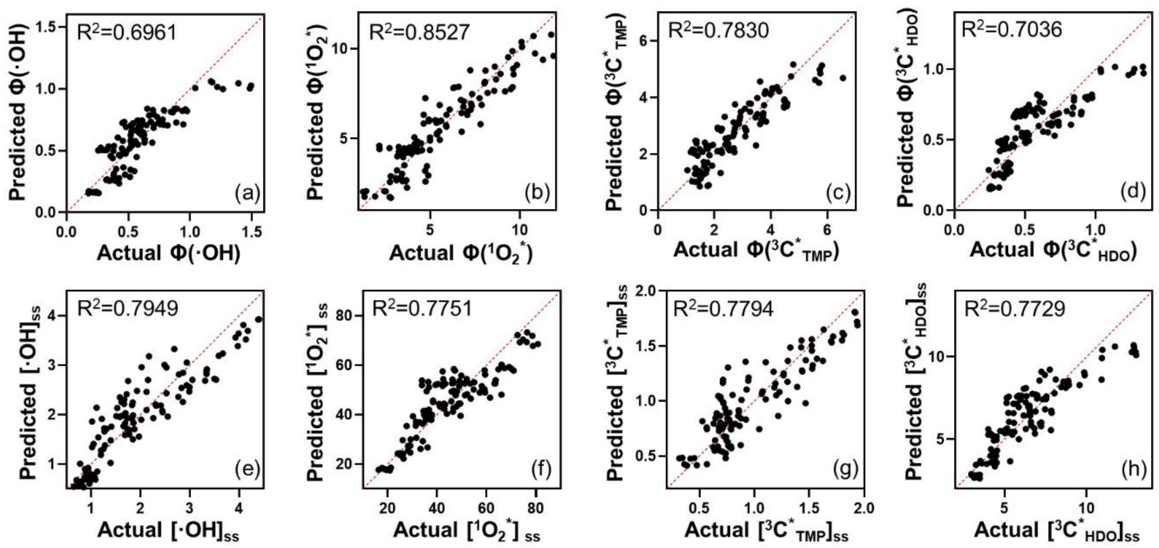

Figure S31. Measured Φ_{RI} and $[RI]_{ss}$ vs. their predicted values from combined OPLS and MLR analyses. The red lines are the 1:1 line. Details of the results of MLR models are shown in Table S15.

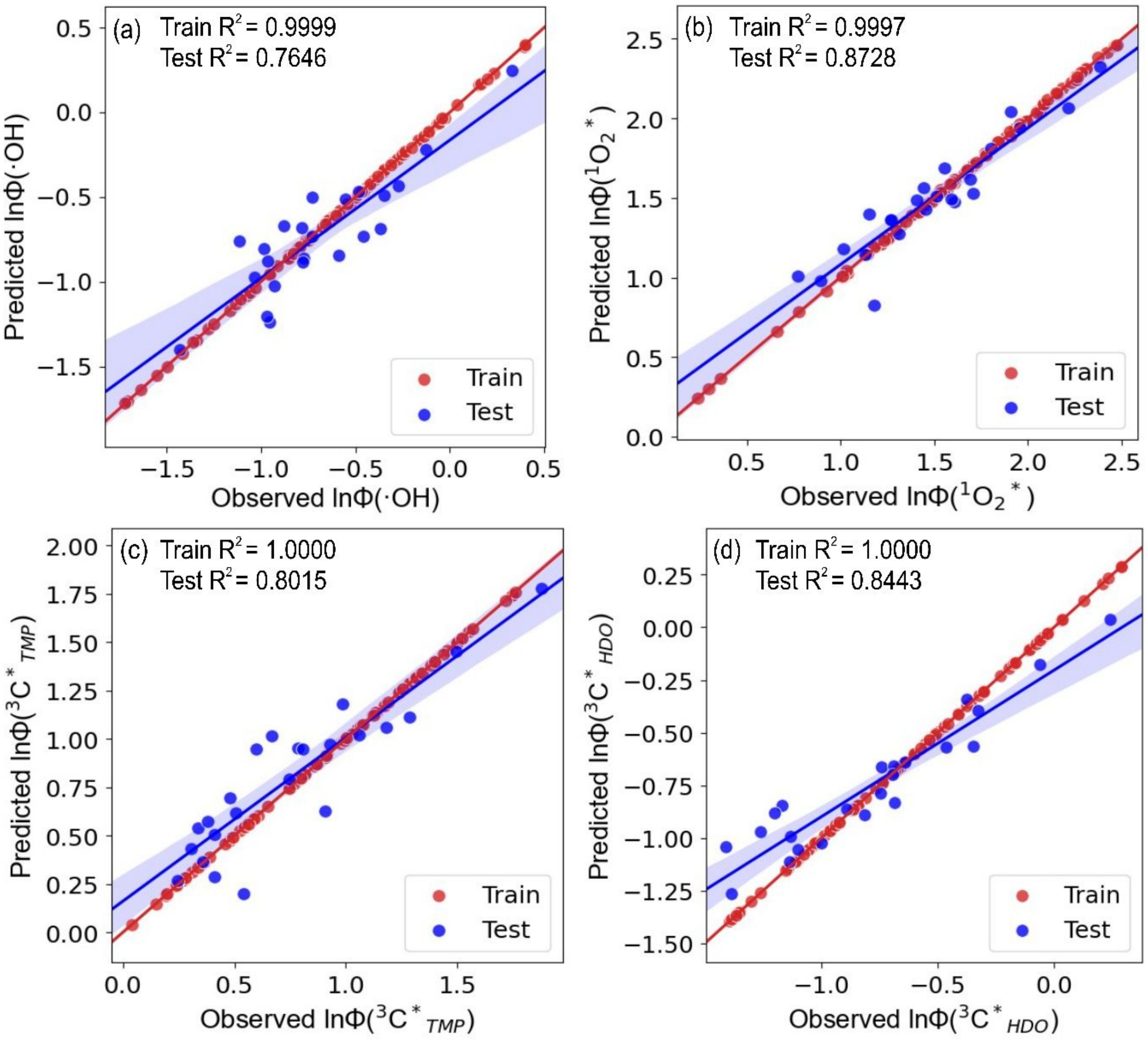

Figure S32. Optimal model predictive performance of (a) $\ln(\Phi_{\bullet OH})$, (b) $\ln(\Phi_{{}^1O_2^*})$, (c) $\ln(\Phi_{{}^3C^*_{TMP}})$, and (d) $\ln(\Phi_{{}^3C^*_{HDO}})$. The red line and blue line represent the regression lines for the training and test sets, respectively. The blue shade represents the 95 % confidence intervals on the regression line for the test set.

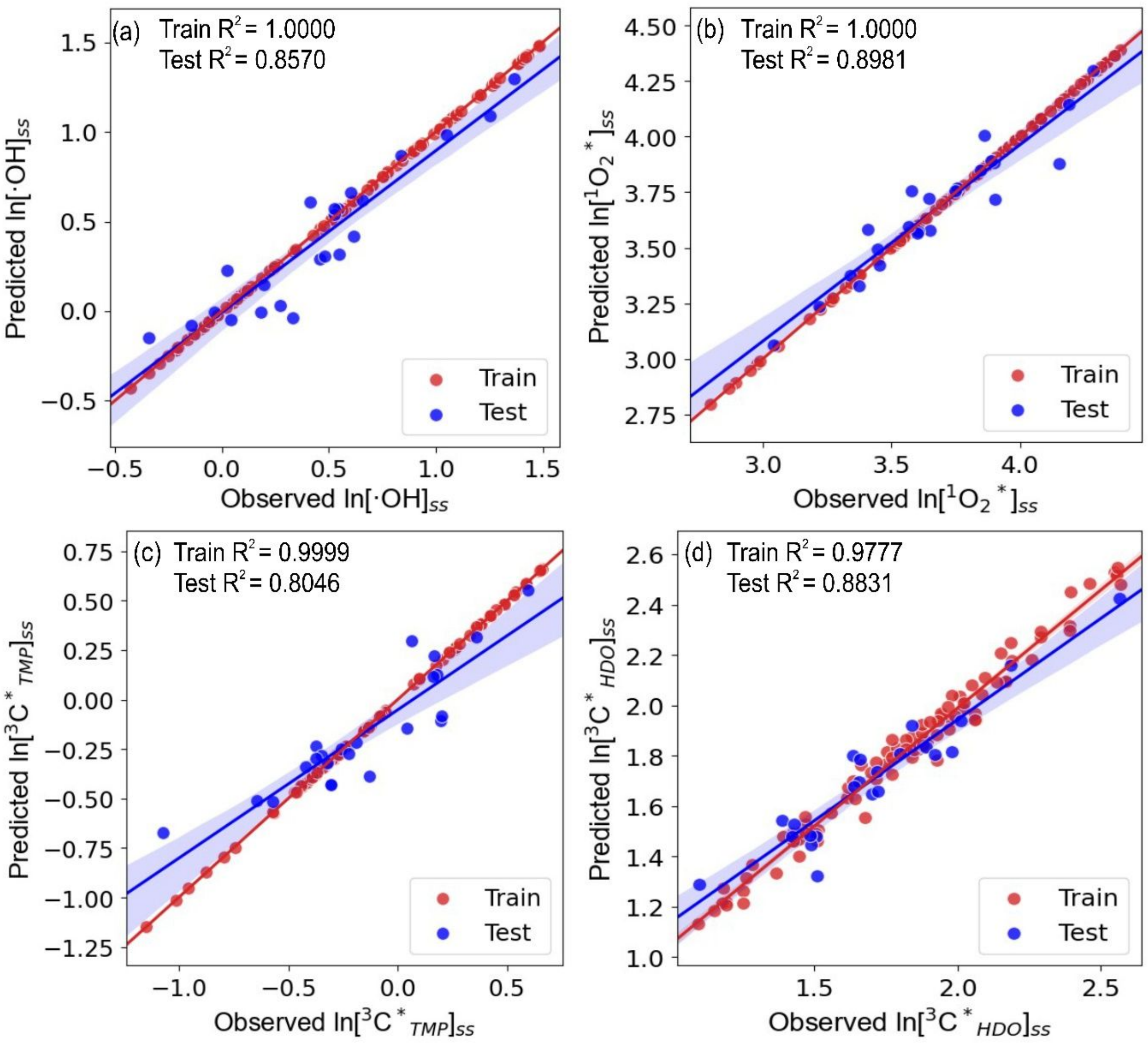

Figure S33. Optimal model predictive performance of (a) $\ln([\bullet OH]_{ss})$, (b) $\ln([{}^1O_2^*]_{ss})$, (c) $\ln([{}^3C_{TMP}^*]_{ss})$, and (d) $\ln([{}^3C_{HDO}^*]_{ss})$. The red line and blue line represent the regression lines for the training and test sets, respectively. The blue shade represents the 95 % confidence intervals on the regression line for the test set.

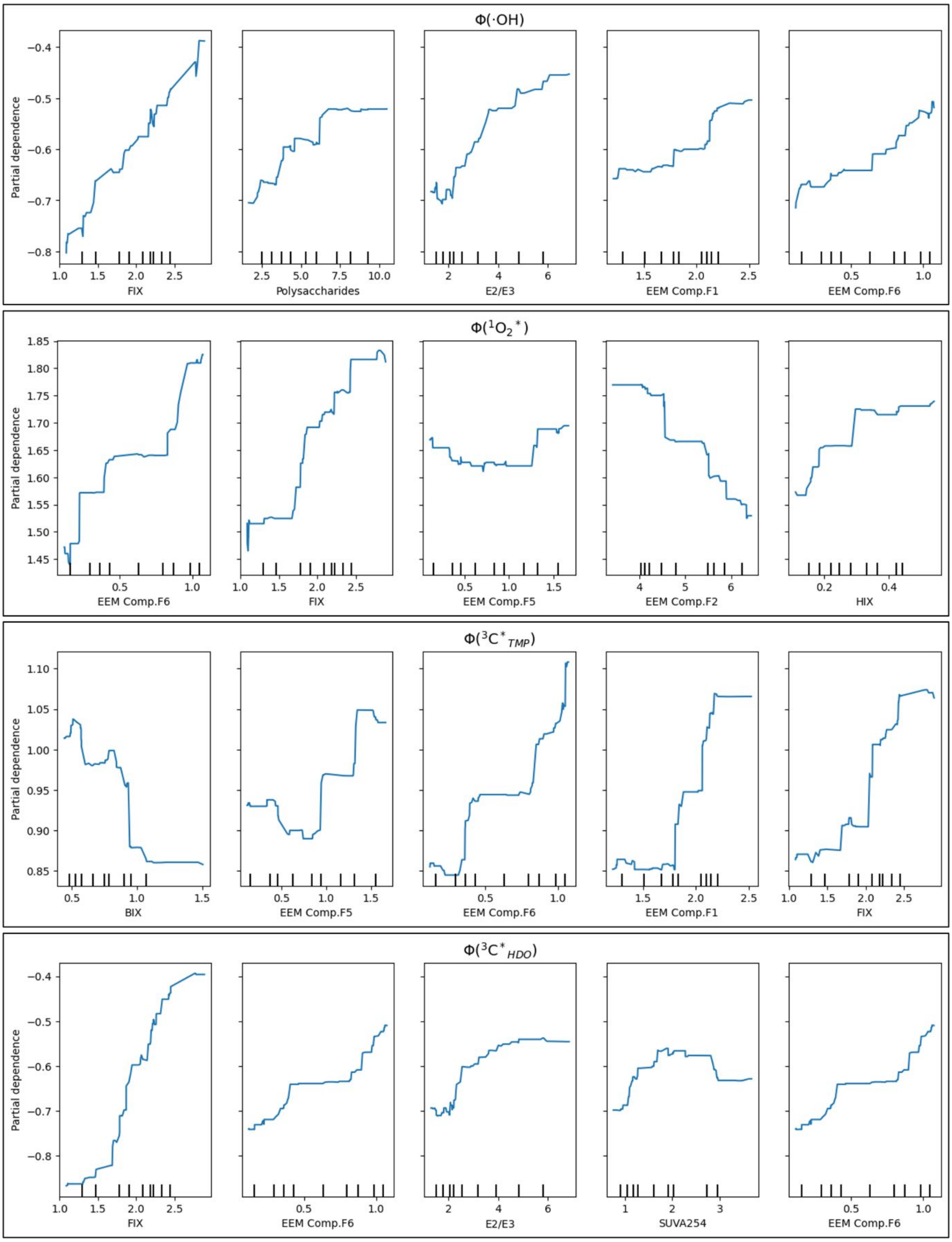

Figure S34. PDP results for the important features for Φ_{RI}. Blue lines indicate the average levels of model prediction.

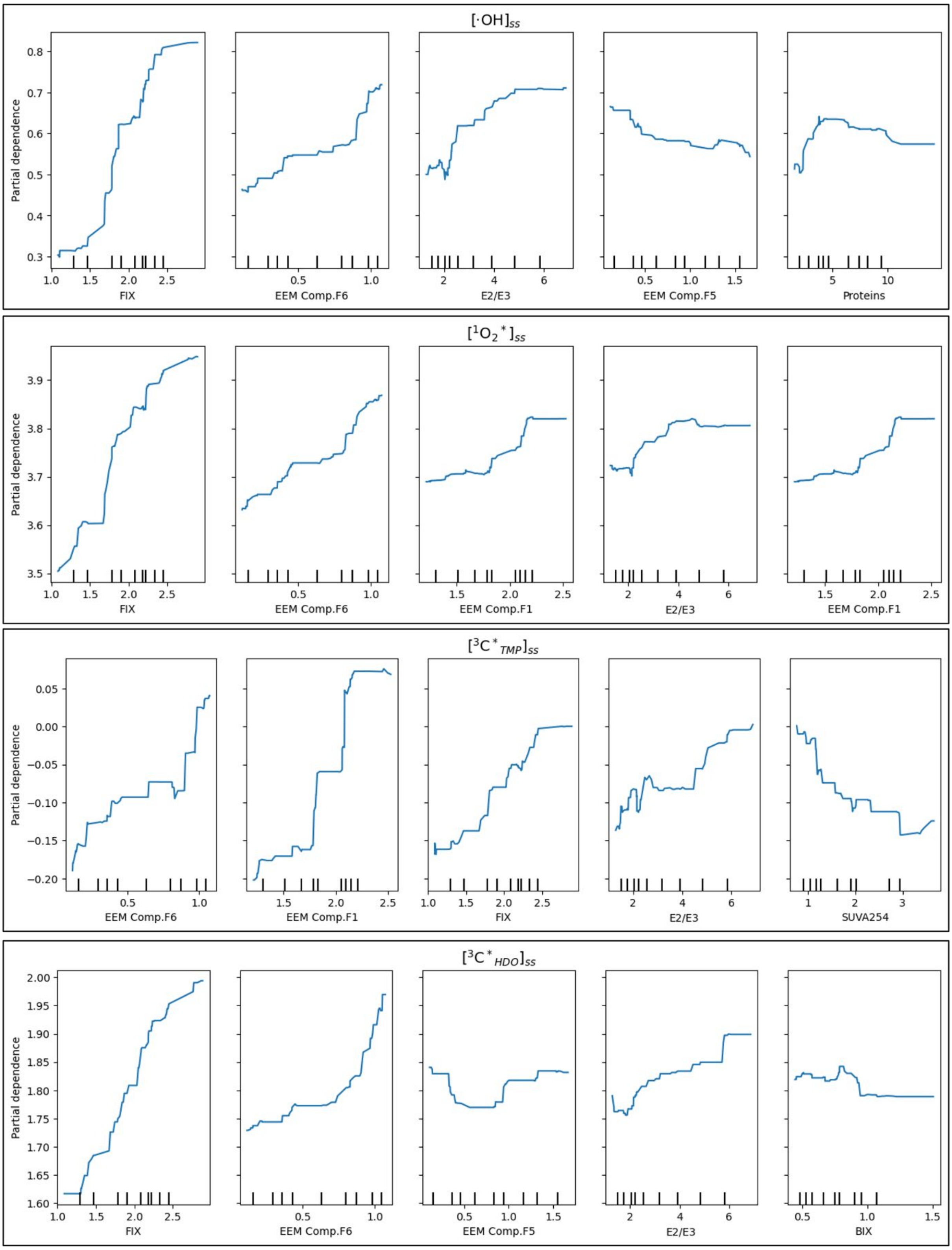

Figure S35. PDP results for the important features for $[RI]_{ss}$. Blue lines indicate the average levels of model prediction.

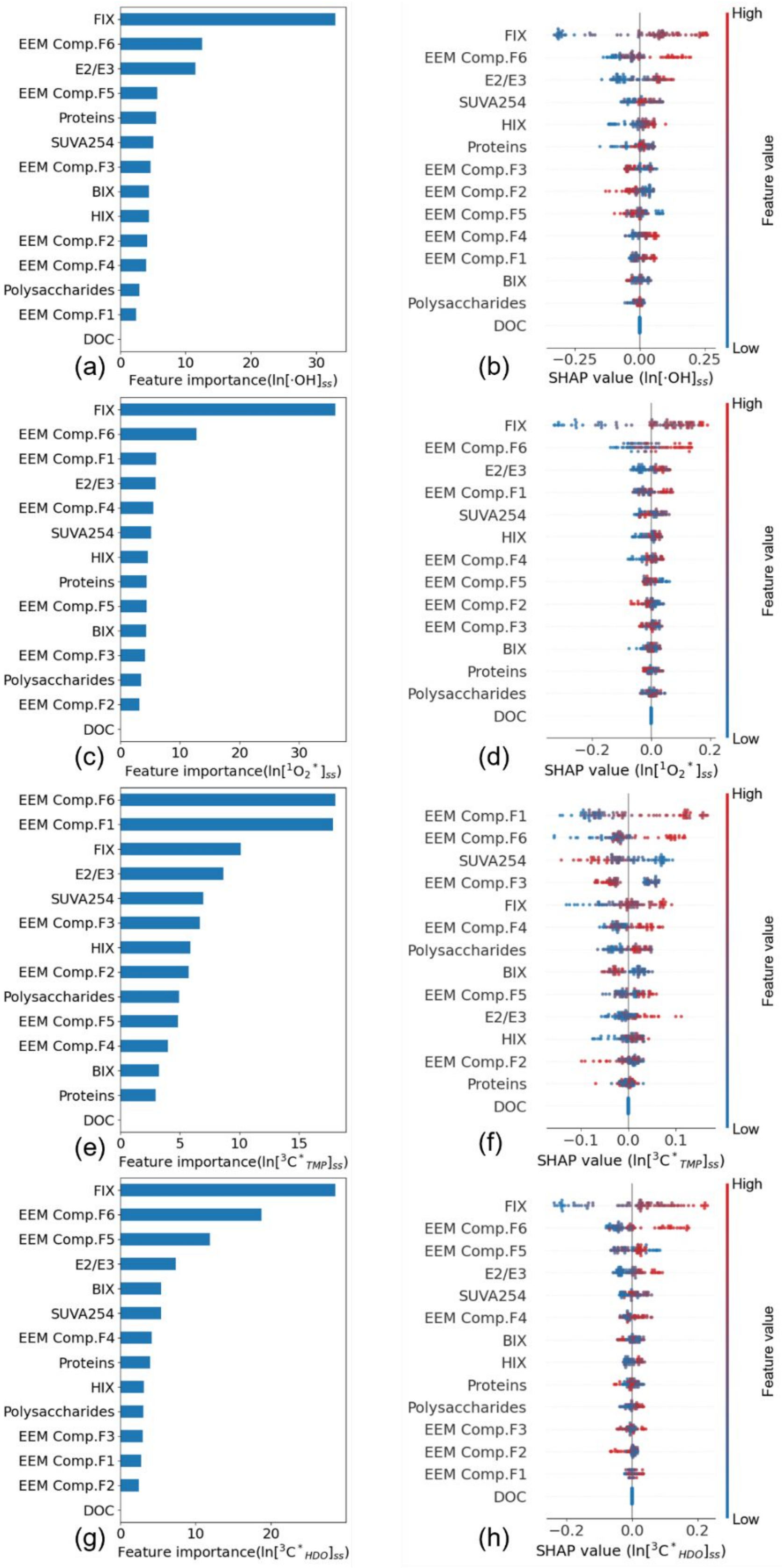

Figure S36. Feature importance rankings from tree-based feature importance and SHAP analysis of (a, b) $\ln([\bullet OH]_{ss})$, (c, d) $\ln([^1O_2^*]_{ss})$, (e, f) $\ln([^3C_{TMP}^*]_{ss})$, and (g, h) $\ln([^3C_{HDO}^*]_{ss}$

). For the SHAP summary interpretation plots (b, d, f, and h), the x-axes are the SHAP values, and the y-axes are the features ranked based on their importance. A positive (negative) SHAP value of a feature in a scenario denotes it positive (negative) contribution to the model prediction. A greater absolute SHAP value implies a greater contribution to the model prediction. The scatter pattern for each feature is composed of the SHAP values for the training data in that feature.

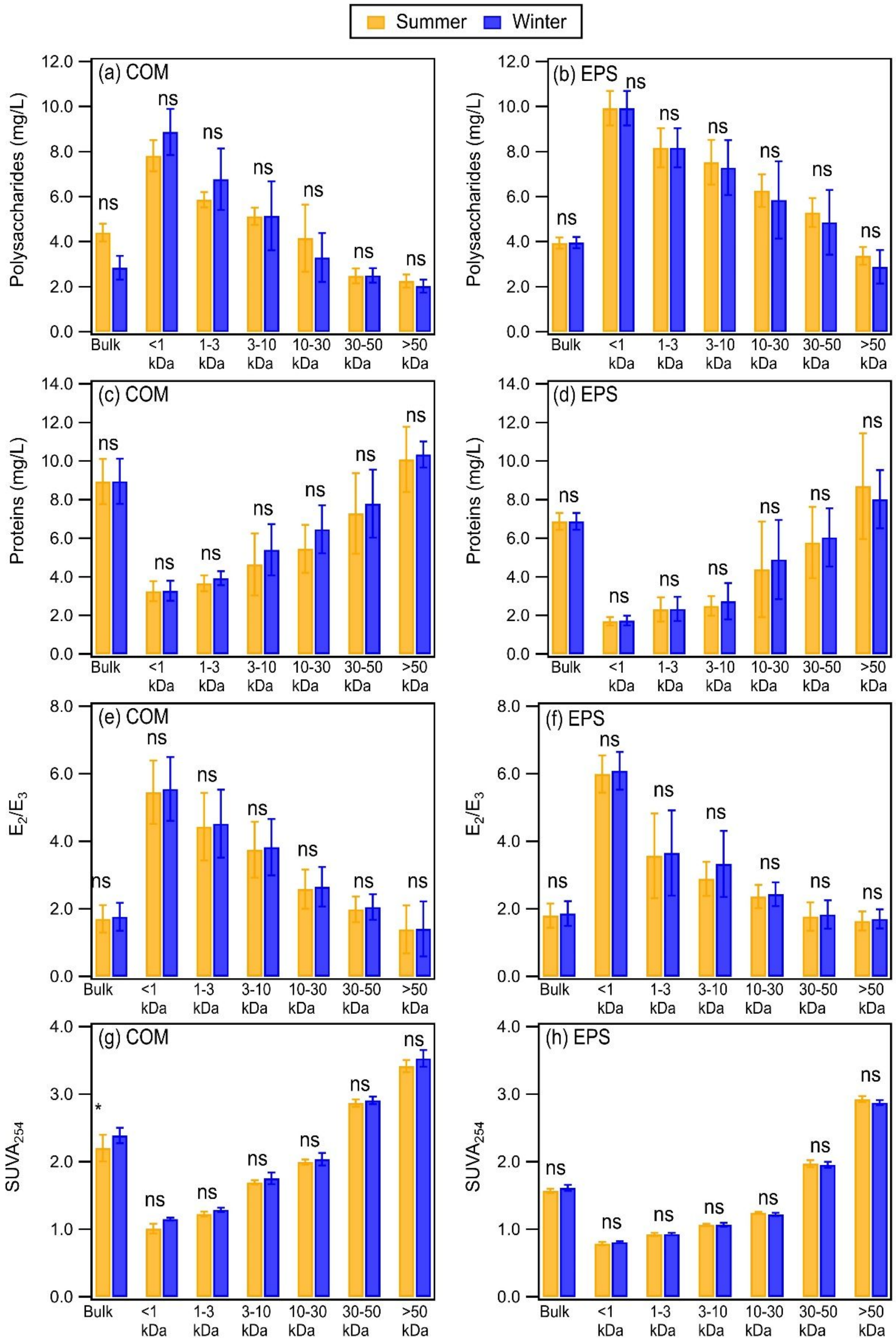

Figure S37. Comparison of the polysaccharides and protein concentrations, E_2/E_3, and $SUVA_{254}$ of bulk and MW-fractionated COM and EPS samples from culturable bacteria in $PM_{2.5}$ collected in summer vs. winter. Differences were found to be not significant ($p > 0.05$).

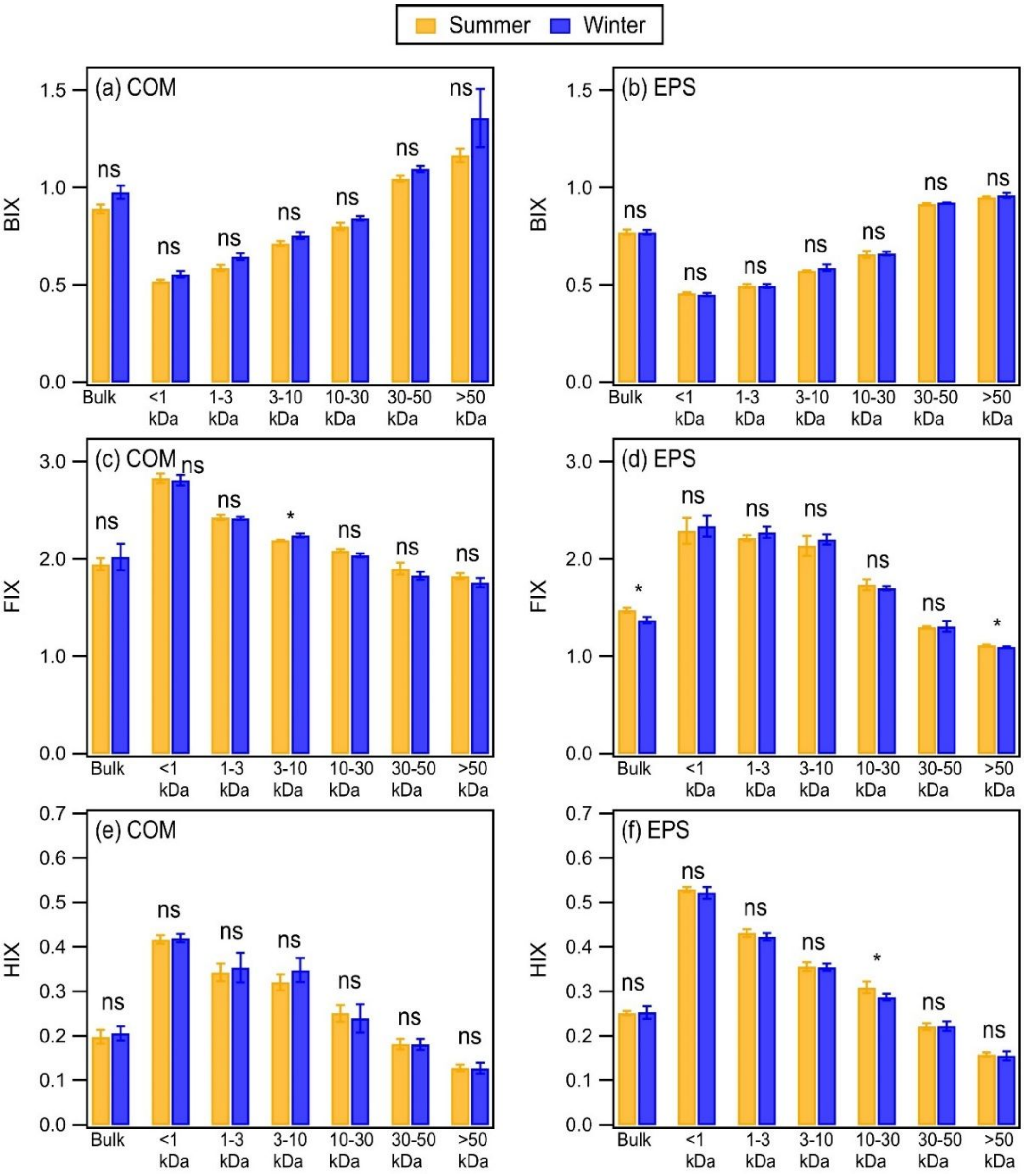

Figure S38. Comparison of the BIX, HIX, and FIX of bulk and MW-fractionated COM and EPS samples from culturable bacteria in $PM_{2.5}$ collected in summer vs. winter ($p < 0.05$, *; $p > 0.05$, not significant (ns)).

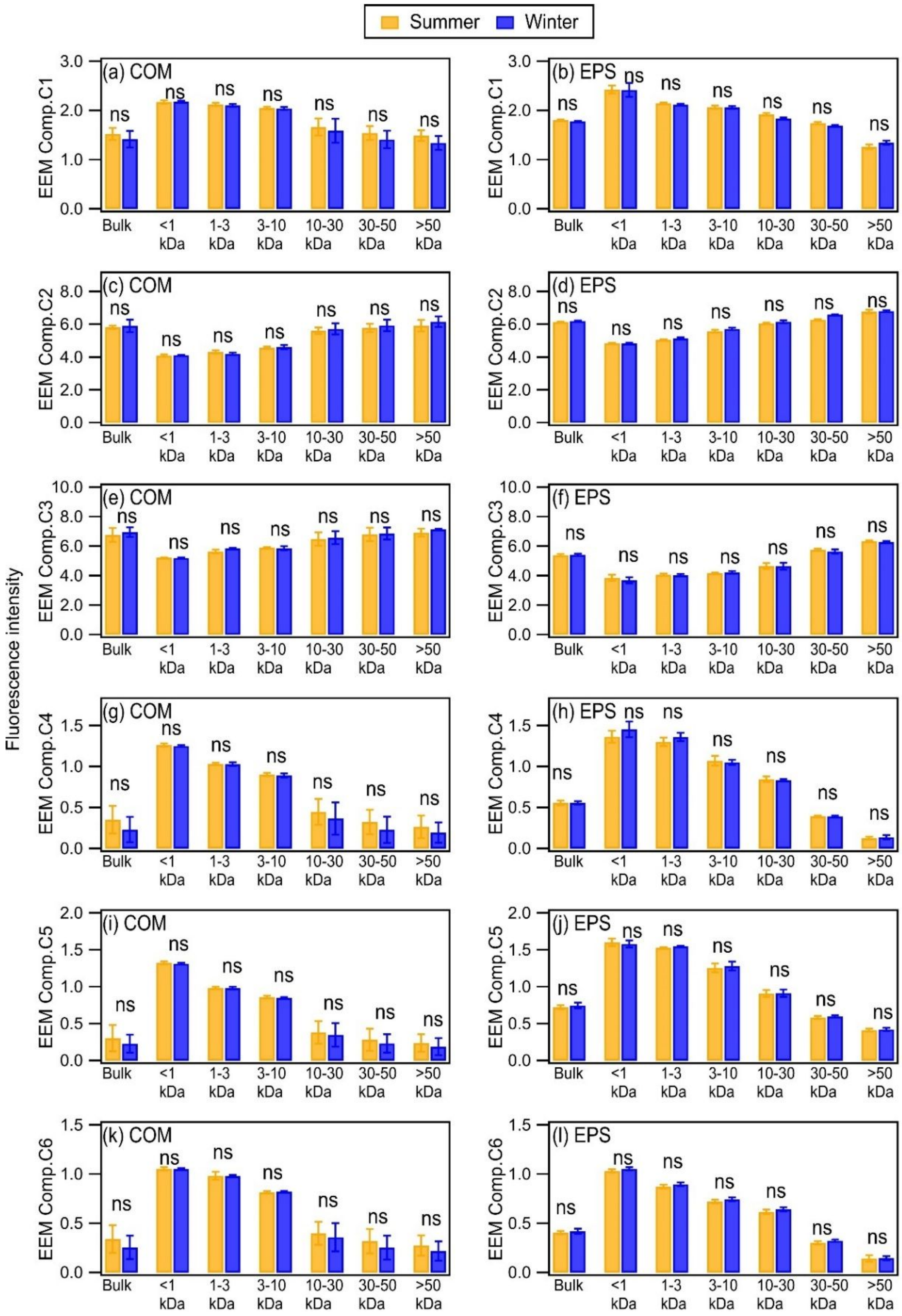

Figure S39. Comparison of the six PARAFAC-extracted components for bulk and MW-fractionated COM and EPS samples from culturable bacteria in $PM_{2.5}$ collected in summer vs. winter. Differences were found to be not significant ($p > 0.05$).

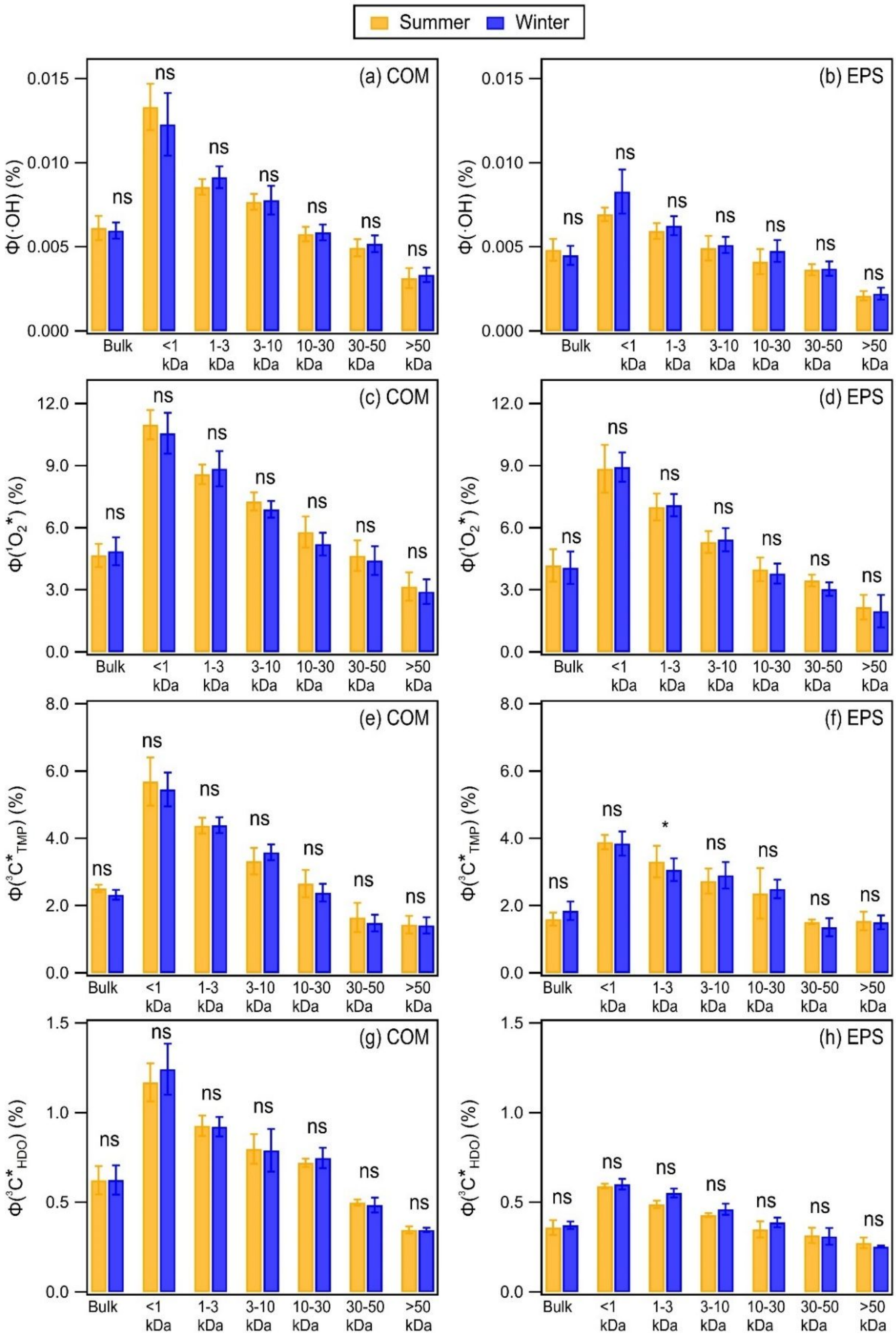

Figure S40. Comparison of the Φ_{RI} for bulk and MW-fractionated COM and EPS samples from culturable bacteria in in $PM_{2.5}$ collected in summer vs. winter ($p < 0.05$, *; $p > 0.05$, not significant (ns)).

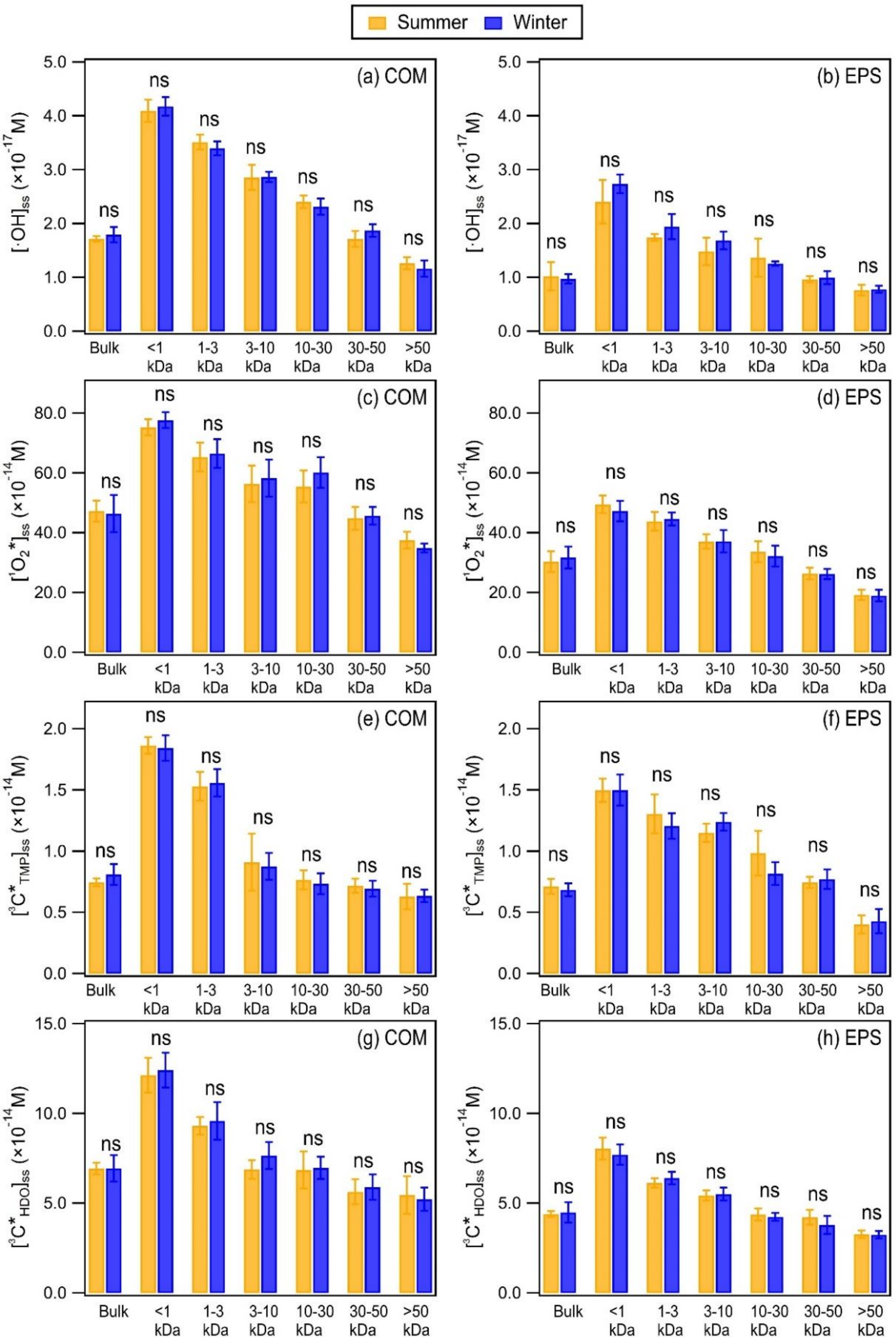

Figure S41. Comparison of the $[RI]_{ss}$ for bulk and MW-fractionated COM and EPS samples from culturable bacteria in $PM_{2.5}$ collected in summer vs. winter. Differences were found to be not significant ($p > 0.05$).

Table S1. Sampling date and relevant information for the $PM_{2.5}$ samples.

Seasons	Date	Bacteria culture media	Component	MW fractions
Summer	Sep. 15, 2023<br>Aug. 1, 2023<br>Aug. 15, 2023	Luria-Bertani broth (LB)<br>Nutrient Broth (NB)<br>Tryptic Soy Broth (TSB)<br>Yeast Extract Peptone Dextrose medium (YPD)	Cellular organic matter (COM)<br><br>Extracellular polymeric substances (EPS)	C1 and E1 (<1 kDa)<br>C2 and E2 (1-3 kDa)<br>C3 and E3 (3-10 kDa)<br>C4 and E4 (10-30 kDa)<br>C5 and E5 (30-50 kDa)<br>C6 and E6 (>50 kDa)<br>C0 and E0 (Bulk)
Winter [a]	Jan. 15, 2024<br>Feb. 1, 2024<br>Feb. 15, 2024<br>Mar. 1, 2024<br>Mar. 15, 2024<br>Mar. 20, 2024			
2 seasons × 4 culture media × 2 components × 7 fractions = 112 samples				

a: There was only one bacteria genus cultivated from winter $PM_{2.5}$ samples collected in Jan and Feb 2024. Thus, three additional $PM_{2.5}$ samples were collected in Mar 2024 to ensure that the winter dataset was not biased towards one bacteria genus. The DNA data of winter samples was taken as an average of the six days.

Table S2. Identified components from EEM fluorescence spectra by PARAFAC analysis.

Component	Excitation (nm) /Emission (nm)	Putative chromophores
F1	280, 373, 445/523	Flavins-like[40]
F2	275/300	Tyrosine-like[15, 40]
F3	290/340	Tryptophan-like[15, 40]
F4	250, 355/455	HULIS-1 (highly oxygenated species)[13, 15, 41]
F5	245/420	Fulvic-like[13]
F6	251,314/415	HULIS-2 (less oxygenated species)[13, 15, 41]

Table S3. UPLC-PDA methods

Analyte	Column	Mobile phase	Flowrate	Injection volume	Detected wavelength	Retention time
Phenol	Kinetex Polar C18 LC column (100 × 2.1 mm, 2.6 µm, 100 Å, Phenomenex, USA) with a security guard and a Polar C18 pre-column	A: water; B: ACN<br>0 min 80% A<br>1.0 min 80% A<br>2.0 min 65% A<br>5.0 min 65% A<br>6.0 min 80% A<br>7.0 min 80% A	0.3 ml/min	15 µL	210 nm	2.86 min
FFA		A: water; B: ACN<br>0-6.0 min 97% A	0.3 ml/min	10 µL	216 nm	3.12 min

TMP	A: water; B: ACN<br>0 min 60% A<br>3.0 min 60% A<br>5.0 min 20% A<br>6.0 min 20% A<br>7.0 min 60% A<br>8.0 min 60% A	0.3 ml/min	10 μL	278 nm	3.75 min
t,t-HDO	A: FA; B: ACN<br>0 min 90% A<br>2.0 min 90% A<br>10.0 min 85% A<br>12.0 min 85% A<br>12.5 min 90% A<br>13.0 min 90% A	0.2 ml/min	5 μL	230 nm	t,t-HDO: 10.08 min<br>c,c-HDO: 8.69 min

Table S4. Performance of the ML (RF, GBDT, LGB, XGB, and CB) models in predicting the $\ln(\Phi_{RI})$ and $\ln([RI]_{ss})$ values.

Dataset	Metrics	Default RF	Default GBDT	Default XGB	Default LGB	Default CB
$\ln(\Phi_{\cdot OH})$	R2 train	0.9859	0.9985	0.9999	0.8952	0.9994
	R2 CV	0.8627	0.8512	0.8038	0.7613	0.8669
	$RMSE_{train}$	0.0544	0.0176	0.0006	0.1486	0.0105
	$RMSE_{CV}$	0.1649	0.1685	0.1890	0.2193	0.1622
$\ln(\Phi_{^1O_2^*})$	R2 train	0.9803	0.9982	0.9999	0.9178	0.9995
	R2 CV	0.8489	0.8400	0.7729	0.8161	0.8478
	$RMSE_{train}$	0.0689	0.0203	0.0007	0.1409	0.0099
	$RMSE_{CV}$	0.1864	0.1889	0.2228	0.2062	0.1872
$\ln(\Phi_{^3C^*_{TMP}})$	R2 train	0.9812	0.9979	0.9999	0.9386	0.9992
	R2 CV	0.8331	0.7907	0.7693	0.8032	0.8312
	$RMSE_{train}$	0.0616	0.0201	0.0006	0.1114	0.0125
	$RMSE_{CV}$	0.1778	0.1983	0.2032	0.1935	0.1789
$\ln(\Phi_{^3C^*_{HDO}})$	R2 train	0.9883	0.9994	0.9999	0.9338	0.9996
	R2 CV	0.9040	0.9151	0.8618	0.7934	0.9295
	$RMSE_{train}$	0.0465	0.0097	0.0005	0.1111	0.0076
	$RMSE_{CV}$	0.1314	0.1242	0.1567	0.1929	0.1138
$\ln([\cdot OH]_{ss})$	R2 train	0.9887	0.9991	0.9999	0.9475	0.9995
	R2 CV	0.9010	0.9193	0.8647	0.8494	0.9121
	$RMSE_{train}$	0.0540	0.0144	0.0007	0.1165	0.0108
	$RMSE_{CV}$	0.1548	0.1394	0.1798	0.1927	0.1434
$\ln([^1O_2^*]_{ss})$	R2 train	0.9901	0.9988	0.9999	0.9342	0.9995
	R2 CV	0.9066	0.9174	0.8298	0.7528	0.9140
	$RMSE_{train}$	0.0379	0.0131	0.0006	0.0983	0.0080
	$RMSE_{CV}$	0.1077	0.1050	0.1440	0.1792	0.1058
$\ln([^3C^*_{TMP}]_{ss})$	R2 train	0.9900	0.9990	0.9999	0.9491	0.9996
	R2 CV	0.9162	0.9196	0.8835	0.8527	0.9122
	$RMSE_{train}$	0.0422	0.0130	0.0006	0.0953	0.0077
	$RMSE_{CV}$	0.1191	0.1169	0.1392	0.1575	0.1221
$\ln([^3C^*_{HDO}]_{ss})$	R2 train	0.9863	0.9978	0.9999	0.9082	0.9992
	R2 CV	0.8702	0.8655	0.8436	0.7332	0.8527
	$RMSE_{train}$	0.0422	0.0167	0.0007	0.1095	0.0098
	$RMSE_{CV}$	0.1171	0.1185	0.1323	0.1772	0.1239

Table S5. Optimal performance of the CB model for predicting the $\ln(\Phi_{RI})$ and $\ln([RI]_{ss})$ values.

Dataset	R2 train	R2 test	R2 CV	$RMSE_{train}$	$RMSE_{test}$	$RMSE_{CV}$
$\ln(\Phi_{\cdot OH})$	0.9999	0.7646	0.8277 ± 0.0388	0.0048	0.1801	0.1847 ± 0.0136
$\ln(\Phi_{^1O_2^*})$	0.9997	0.8728	0.8790 ± 0.0304	0.0087	0.1391	0.1678 ± 0.0342
$\ln(\Phi_{^3C_{TMP}^*})$	1	0.8015	0.8213 ± 0.0769	0.0009	0.1825	0.1834 ± 0.0429
$\ln(\Phi_{^3C_{HDO}^*})$	1	0.8443	0.9164 ± 0.0205	0.0001	0.1646	0.1236 ± 0.0203
$\ln(\Phi_{^1O_2^*})$	1	0.8570	0.9101 ± 0.0137	0.0001	0.155	0.1483 ± 0.0041
$\ln([^1O_2^*]_{ss})$	1	0.8981	0.8974 ± 0.0352	0.0005	0.0966	0.1163 ± 0.0178
$\ln([^3C_{TMP}^*]_{ss})$	0.9999	0.8046	0.9159 ± 0.0298	0.0035	0.1602	0.1179 ± 0.0198
$\ln([^3C_{HDO}^*]_{ss})$	0.9777	0.8831	0.8551 ± 0.1033	0.054	0.1019	0.1236 ± 0.0324

Table S6. Composition and optical properties of bulk and MW- fractionated COM samples from culturable bacteria in $PM_{2.5}$ (DOC = 5 mg C/L).

	**Seasons**	**C0 (Bulk)**	**C1 (<1 kDa)**	**C2 (1–3 kDa)**	**C3 (3–10 kDa)**	**C4 (10–30 kDa)**	**C5 (30–50 kDa)**	**C6 (>50 kDa)**
Polysaccharides (mg/L)	Summer	4.41 ± 0.39	7.82 ± 0.69	5.87 ± 0.35	5.13 ± 0.38	4.16 ± 1.48	2.49 ± 0.33	2.26 ± 0.29
	Winter	2.84 ± 0.52	8.88 ± 1.03	6.77 ± 1.36	5.15 ± 1.53	3.30 ± 1.08	2.50 ± 0.33	2.03 ± 0.29
Proteins (mg/L)	Summer	8.95 ± 1.16	3.26 ± 0.52	3.66 ± 0.41	4.64 ± 1.60	5.46 ± 1.24	7.29 ± 2.08	10.09 ± 2.78
	Winter	8.95 ± 1.17	3.28 ± 0.52	3.93 ± 0.36	5.41 ± 1.33	6.47 ± 1.25	7.80 ± 1.77	10.34 ± 0.67
E_2/E_3	Summer	1.70 ± 0.41	5.45 ± 0.94	4.44 ± 1.00	3.75 ± 0.83	2.59 ± 0.58	1.99 ± 0.38	1.39 ± 0.71
	Winter	1.76± 0.41	5.55± 0.94	4.52± 1.01	3.83± 0.84	2.66± 0.59	2.05± 0.38	1.87± 0.21
$SUVA_{254}$	Summer	2.20 ± 0.20	1.01 ± 0.07	1.23 ± 0.03	1.70 ± 0.03	2.00 ± 0.04	2.87 ± 0.05	3.42 ± 0.09
	Winter	2.39 ± 0.12	1.15 ± 0.02	1.29 ± 0.03	1.76 ± 0.09	2.04 ± 0.09	2.91 ± 0.06	3.53 ± 0.12
BIX	Summer	0.89 ± 0.02	0.52 ± 0.01	0.59 ± 0.02	0.71 ± 0.01	0.80 ± 0.02	1.05 ± 0.02	1.17 ± 0.03
	Winter	0.98 ± 0.03	0.55 ± 0.02	0.65 ± 0.02	0.76 ± 0.02	0.84 ± 0.01	1.10 ± 0.02	1.36 ± 0.15
FIX	Summer	1.95 ± 0.06	2.83 ± 0.05	2.43 ± 0.03	2.19 ± 0.01	2.08 ± 0.02	1.90 ± 0.06	1.82 ± 0.03
	Winter	2.02 ± 0.14	2.81 ± 0.05	2.42 ± 0.02	2.24 ± 0.02	2.04 ± 0.02	1.83 ± 0.04	1.76 ± 0.05
HIX	Summer	0.20 ± 0.02	0.42 ± 0.01	0.34 ± 0.02	0.32 ± 0.02	0.25 ± 0.02	0.18 ± 0.01	0.13 ± 0.01
	Winter	0.21 ± 0.02	0.42 ± 0.01	0.35 ± 0.03	0.35 ± 0.03	0.24 ± 0.03	0.18 ± 0.01	0.13 ± 0.01

Table S7. Composition and optical properties of bulk and MW- fractionated EPS samples from culturable bacteria in $PM_{2.5}$ (DOC = 5 mg C/L).

	**Seasons**	**E0 (Bulk)**	**E1 (<1 kDa)**	**E2 (1–3 kDa)**	**E3 (3–10 kDa)**	**E4 (10–30 kDa)**	**E5 (30–50 kDa)**	**E6 (>50 kDa)**
Polysaccharides (mg/L)	Summer	3.95 ± 0.23	9.93 ± 0.71	8.17 ± 0.81	7.41 ± 1.04	6.06 ± 1.24	5.08 ± 1.05	3.13 ± 0.61
	Winter	3.96 ± 0.25	9.93 ± 0.77	8.18 ± 0.87	7.29 ± 1.22	5.85 ± 1.72	4.86 ± 1.43	2.89 ± 0.74
Proteins (mg/L)	Summer	6.88 ± 0.41	1.72 ± 0.22	2.33 ± 0.58	2.62 ± 0.71	4.65 ± 2.12	5.92 ± 1.57	8.36 ± 2.08
	Winter	6.88 ± 0.44	1.74 ± 0.26	2.34 ± 0.63	2.74 ± 0.95	4.90 ± 2.05	6.05 ± 1.51	8.02 ± 1.51
E_2/E_3	Summer	1.8 ± 0.36	5.99 ± 0.55	3.57 ± 1.25	2.89 ± 0.51	2.37 ± 0.35	1.77 ± 0.42	1.64 ± 0.28
	Winter	1.86± 0.36	6.09± 0.56	3.65± 1.26	3.33± 0.98	2.44± 0.35	1.84± 0.42	1.71± 0.29
$SUVA_{254}$	Summer	1.59 ± 0.04	0.8 ± 0.02	0.93 ± 0.02	1.07 ± 0.02	1.23 ± 0.02	1.96 ± 0.05	2.90 ± 0.05
	Winter	1.61 ± 0.05	0.81 ± 0.01	0.93 ± 0.02	1.07 ± 0.03	1.22 ± 0.03	1.95 ± 0.05	2.88 ± 0.04
BIX	Summer	0.77 ± 0.01	0.45 ± 0.01	0.5 ± 0.01	0.58 ± 0.01	0.66 ± 0.01	0.92 ± 0.01	0.96 ± 0.01
	Winter	0.77 ± 0.01	0.45 ± 0.01	0.50 ± 0.01	0.59 ± 0.02	0.66 ± 0.01	0.92 ± 0.00	0.96 ± 0.01
FIX	Summer	1.42 ± 0.06	2.31 ± 0.12	2.24 ± 0.05	2.17 ± 0.08	1.72 ± 0.04	1.30 ± 0.04	1.10 ± 0.01
	Winter	1.37 ± 0.03	2.34 ± 0.11	2.27 ± 0.06	2.20 ± 0.05	1.70 ± 0.02	1.31 ± 0.05	1.09 ± 0.01
HIX	Summer	0.25 ± 0.01	0.53 ± 0.01	0.43 ± 0.01	0.36 ± 0.01	0.3 ± 0.02	0.22 ± 0.01	0.16 ± 0.01
	Winter	0.25 ± 0.01	0.52 ± 0.01	0.42 ± 0.01	0.36 ± 0.01	0.29 ± 0.01	0.22 ± 0.01	0.15 ± 0.01

Table S8. Composition and optical properties of bulk and MW- fractionated COM samples extracted from pure laboratory cultures (all were fixed to DOC = 5 mg C/L).

	**Strains**	**C0 (Bulk)**	**C1 (<1 kDa)**	**C2 (1–3 kDa)**	**C3 (3–10 kDa)**	**C4 (10–30 kDa)**	**C5 (30–50 kDa)**	**C6 (>50 kDa)**
Polysaccharides (mg/L)	*B. subtilis*	4.72 ± 0.08	8.16 ± 0.20	5.96 ± 0.28	4.94 ± 0.44	4.26 ± 0.48	2.50 ± 0.27	2.30 ± 0.03
	P. putida	3.07 ± 0.31	8.26 ± 0.22	6.76 ± 0.54	4.45 ± 0.12	2.52 ± 0.54	2.51 ± 0.30	1.81 ± 0.27
	E. hormaechei B0910	4.15 ± 0.04	7.59 ± 0.09	5.85 ± 0.12	5.40 ± 0.20	4.11 ± 0.21	2.51 ± 0.12	2.25 ± 0.02
	E. hormaechei pf0910	2.66 ± 0.12	9.60 ± 0.09	6.87 ± 0.21	5.91 ± 0.05	4.12 ± 0.21	2.53 ± 0.12	2.27 ± 0.11
Proteins (mg/L)	*B. subtilis*	8.66 ± 0.41	3.74 ± 0.05	4.00 ± 0.09	5.70 ± 0.35	6.56 ± 0.17	8.14 ± 0.11	10.87 ± 0.12
	P. putida	8.67 ± 0.46	3.75 ± 0.05	4.01 ± 0.29	5.72 ± 0.24	7.07 ± 0.43	9.15 ± 0.35	10.86 ± 0.26
	E. hormaechei B0910	9.34 ± 0.18	2.83 ± 0.02	3.38 ± 0.04	3.64 ± 0.15	4.43 ± 0.08	5.53 ± 0.05	8.43 ± 0.06
	E. hormaechei pf0910	9.34 ± 0.18	2.85 ± 0.02	3.90 ± 0.12	5.17 ± 0.10	5.95 ± 0.17	6.54 ± 0.14	9.94 ± 0.10
E_2/E_3	*B. subtilis*	1.98 ± 0.06	5.3 ± 0.20	4.72 ± 0.10	3.84 ± 0.18	2.5 ± 0.14	1.86 ± 0.20	1.16 ± 0.18
	P. putida	2.04 ± 0.16	5.42 ± 0.04	4.64 ± 0.12	3.92 ± 0.26	2.58 ± 0.06	1.92 ± 0.22	1.12 ± 0.18
	E. hormaechei B0910	1.46 ± 0.06	4.76 ± 0.02	4.04 ± 0.06	3.72 ± 0.08	2.72 ± 0.10	2.16 ± 0.10	1.66 ± 0.14
	E. hormaechei pf0910	1.52 ± 0.16	4.84 ± 0.18	4.12 ± 0.14	3.8 ± 0.18	2.78 ± 0.22	2.22 ± 0.18	1.72 ± 0.16
$SUVA_{254}$	*B. subtilis*	2.14 ± 0.10	1.04 ± 0.17	1.24 ± 0.34	1.71 ± 0.36	2.04 ± 0.24	2.89 ± 0.15	3.52 ± 0.28
	P. putida	2.38 ± 0.08	1.16 ± 0.31	1.33 ± 0.39	1.74 ± 0.41	2.12 ± 0.27	2.97 ± 0.17	3.56 ± 0.36
	E. hormaechei B0910	2.30 ± 0.08	1.00 ± 0.12	1.23 ± 0.14	1.71 ± 0.24	1.98 ± 0.19	2.89 ± 0.28	3.37 ± 0.26
	E. hormaechei pf0910	2.43 ± 0.19	1.17 ± 0.10	1.28 ± 0.14	1.81 ± 0.32	1.98 ± 0.07	2.89 ± 0.20	3.55 ± 0.16
BIX	*B. subtilis*	0.91 ± 0.04	0.53 ± 0.02	0.60 ± 0.03	0.72 ± 0.03	0.81 ± 0.04	1.06 ± 0.03	1.17 ± 0.02
	P. putida	0.98 ± 0.02	0.56 ± 0.03	0.65 ± 0.02	0.77 ± 0.05	0.85 ± 0.04	1.11 ± 0.04	1.44 ± 0.03
	E. hormaechei B0910	0.89 ± 0.02	0.52 ± 0.02	0.59 ± 0.02	0.72 ± 0.01	0.81 ± 0.02	1.05 ± 0.01	1.28 ± 0.04
	E. hormaechei pf0910	0.99 ± 0.03	0.56 ± 0.03	0.65 ± 0.03	0.76 ± 0.02	0.85 ± 0.03	1.01 ± 0.03	1.36 ± 0.02
FIX	*B. subtilis*	2.01 ± 0.07	2.87 ± 0.05	2.44 ± 0.05	2.21 ± 0.02	2.10 ± 0.04	1.88 ± 0.05	1.86 ± 0.02
	P. putida	2.14 ± 0.04	2.80 ± 0.01	2.44 ± 0.04	2.27 ± 0.03	2.06 ± 0.04	1.85 ± 0.05	1.80 ± 0.02
	E. hormaechei B0910	1.92 ± 0.05	2.83 ± 0.03	2.46 ± 0.03	2.21 ± 0.01	2.10 ± 0.01	1.95 ± 0.01	1.81 ± 0.04
	E. hormaechei pf0910	1.93 ± 0.07	2.87 ± 0.04	2.44 ± 0.02	2.25 ± 0.02	2.06 ± 0.03	1.84 ± 0.05	1.74 ± 0.04
HIX	*B. subtilis*	0.20 ± 0.01	0.43 ± 0.01	0.34 ± 0.02	0.32 ± 0.01	0.25 ± 0.01	0.19 ± 0.01	0.14 ± 0.01
	P. putida	0.21 ± 0.01	0.42 ± 0.01	0.36 ± 0.02	0.36 ± 0.02	0.25 ± 0.02	0.18 ± 0.01	0.14 ± 0.01
	E. hormaechei B0910	0.20 ± 0.01	0.42 ± 0.01	0.36 ± 0.01	0.33 ± 0.01	0.27 ± 0.01	0.19 ± 0.01	0.13 ± 0.02
	E. hormaechei pf0910	0.21 ± 0.01	0.44 ± 0.01	0.36 ± 0.01	0.35 ± 0.01	0.25 ± 0.02	0.19 ± 0.01	0.13 ± 0.01

Table S9. Composition and optical properties of bulk and MW- fractionated EPS samples extracted from pure laboratory cultures (all were fixed to DOC = 5 mg C/L).

	**Strains**	**E0 (Bulk)**	**E1 (<1 kDa)**	**E2 (1–3 kDa)**	**E3 (3–10 kDa)**	**E4 (10–30 kDa)**	**E5 (30–50 kDa)**	**E6 (>50 kDa)**
Polysaccharides (mg/L)	*B. subtilis*	4.05 ± 0.16	9.23 ± 0.16	7.92 ± 0.34	7.21 ± 0.23	5.98 ± 0.30	5.16 ± 0.30	3.67 ± 0.45
	P. putida	4.07 ± 0.50	9.73 ± 0.12	7.93 ± 1.01	7.22 ± 0.33	5.14 ± 0.87	4.27 ± 0.11	2.69 ± 0.08
	E. hormaechei B0910	4.30 ± 0.08	8.67 ± 0.28	7.70 ± 0.16	6.71 ± 0.99	5.89 ± 0.06	5.00 ± 0.08	2.91 ± 0.09
	E. hormaechei pf0910	3.89 ± 0.08	9.03 ± 0.08	8.35 ± 0.14	7.70 ± 0.62	6.38 ± 0.33	5.03 ± 0.48	3.33 ± 0.09
Proteins (mg/L)	*B. subtilis*	7.07 ± 0.23	1.82 ± 0.23	2.78 ± 0.12	2.79 ± 0.14	5.92 ± 0.26	6.71 ± 0.26	8.59 ± 0.26
	P. putida	7.08 ± 0.56	1.89 ± 1.26	2.79 ± 1.25	3.26 ± 0.91	5.93 ± 0.91	6.72 ± 0.50	9.23 ± 0.43
	E. hormaechei B0910	6.49 ± 0.28	1.66 ± 0.60	1.84 ± 0.59	2.35 ± 0.56	3.27 ± 0.95	4.29 ± 0.27	6.38 ± 0.13
	E. hormaechei pf0910	7.03 ± 2.16	1.55 ± 0.61	1.99 ± 0.58	2.14 ± 0.57	3.59 ± 0.08	6.09 ± 0.72	7.45 ± 0.44
E_2/E_3	*B. subtilis*	1.8 0± 0.04	5.58 ± 0.04	4.56 ± 0.04	2.66 ± 0.04	2.68 ± 0.04	1.94 ± 0.04	1.66 ± 0.04
	P. putida	1.86 ± 0.10	5.72 ± 0.08	4.66 ± 0.08	3.46 ± 0.18	2.76 ± 0.18	2.00 ± 0.20	1.72 ± 0.20
	E. hormaechei B0910	2.20 ± 0.10	5.36 ± 0.14	4.08 ± 0.10	3.48 ± 0.12	2.92 ± 0.10	2.44 ± 0.10	2.04 ± 0.10
	E. hormaechei pf0910	1.54 ± 0.1	5.84 ± 0.12	3.26 ± 0.12	2.96 ± 0.12	2.14 ± 0.02	2.12 ± 0.02	1.40 ± 0.02
$SUVA_{254}$	*B. subtilis*	1.56 ± 0.02	0.81 ± 0.05	0.94 ± 0.02	1.07 ± 0.02	1.25 ± 0.03	1.95 ± 0.01	2.98 ± 0.02
	P. putida	1.64 ± 0.01	0.81 ± 0.01	0.93 ± 0.03	1.10 ± 0.02	1.24 ± 0.06	1.98 ± 0.01	2.89 ± 0.02
	E. hormaechei B0910	1.61 ± 0.07	0.80 ± 0.08	0.94 ± 0.06	1.07 ± 0.01	1.24 ± 0.03	1.98 ± 0.08	2.91 ± 0.13
	E. hormaechei pf0910	1.61 ± 0.04	0.81 ± 0.06	0.93 ± 0.05	1.06 ± 0.07	1.24 ± 0.02	2.01 ± 0.02	2.92 ± 0.02
BIX	*B. subtilis*	0.79 ± 0.01	0.47 ± 0.01	0.50 ± 0.01	0.58 ± 0.01	0.68 ± 0.01	0.92 ± 0.01	0.96 ± 0.01
	P. putida	0.77 ± 0.02	0.46 ± 0.01	0.50 ± 0.02	0.59 ± 0.01	0.67 ± 0.01	0.93 ± 0.01	0.96 ± 0.01
	E. hormaechei B0910	0.78 ± 0.02	0.46 ± 0.02	0.51 ± 0.01	0.60 ± 0.06	0.66 ± 0.03	0.89 ± 0.01	0.97 ± 0.03
	E. hormaechei pf0910	0.78 ± 0.05	0.46 ± 0.01	0.50 ± 0.01	0.59 ± 0.02	0.66 ± 0.01	0.75 ± 0.01	1.00 ± 0.01
FIX	*B. subtilis*	1.49 ± 0.01	2.35 ± 0.01	2.25 ± 0.01	2.21 ± 0.01	1.78 ± 0.03	1.31 ± 0.02	1.13 ± 0.01
	P. putida	1.38 ± 0.01	2.32 ± 0.01	2.26 ± 0.02	2.17 ± 0.01	1.70 ± 0.01	1.31 ± 0.01	1.11 ± 0.01
	E. hormaechei B0910	1.45 ± 0.07	2.30 ± 0.20	2.29 ± 0.13	2.13 ± 0.35	1.69 ± 0.07	1.35 ± 0.12	1.11 ± 0.02
	E. hormaechei pf0910	1.42 ± 0.16	2.35 ± 0.04	2.25 ± 0.07	2.24 ± 0.11	1.75 ± 0.01	1.29 ± 0.02	1.11 ± 0.01
HIX	*B. subtilis*	0.26 ± 0.01	0.54 ± 0.02	0.44 ± 0.01	0.37 ± 0.01	0.31 ± 0.01	0.23 ± 0.01	0.17 ± 0.01
	P. putida	0.25 ± 0.01	0.52 ± 0.01	0.43 ± 0.01	0.37 ± 0.01	0.29 ± 0.01	0.23 ± 0.01	0.15 ± 0.01
	E. hormaechei B0910	0.26 ± 0.03	0.54 ± 0.03	0.44 ± 0.02	0.37 ± 0.02	0.31 ± 0.07	0.22 ± 0.01	0.16 ± 0.02
	E. hormaechei pf0910	0.26 ± 0.01	0.54 ± 0.02	0.43 ± 0.01	0.35 ± 0.01	0.31 ± 0.01	0.22 ± 0.01	0.17 ± 0.01

Table S10. Descriptions of the optical (absorbance and fluorescence) parameters used in this work.

Parameter	Calculation	Description
E_2/E_3	$E_2/E_3 = \frac{\text{Absorbance at 254 nm}}{\text{Absorbance at 365 nm}}$ E_2/E_3 is the ratio of the absorbance at 250 nm to the absorbance at 365 nm.	The E_2/E_3 ratio denotes the size of chromophores. A larger E_2/E_3 value indicates smaller molecular size.
$SUVA_{254}$	$SUVA_{254} = \frac{\text{Absorbance at 254 nm}}{[DOC] \times l}$ $SUVA_{254}$ is the DOC-normalized absorbance coefficient. [DOC] is the dissolved (water-soluble) organic concentration. l is the pathlength.	$SUVA_{254}$ can be used as an indication of the degree of aromaticity for the chromophores. A larger $SUVA_{254}$ value indicates a higher degree of aromaticity.
FIX	$\text{FIX} = \frac{\text{Fluorescence intensity at 470 nm}}{\text{Fluorescence intensity at 520 nm}}$ FIX is the fluorescence index.	FIX can be used to determine the aromaticity of fluorophores. A smaller FIX value indicates a higher degree of aromaticity.
HIX	$\text{FIX} = \frac{\text{Sum of Fluorescence intensit ies } (435 - 480\text{nm})}{\text{Sum of Fluorescence intensit ies } (300 - 345\text{nm})}$ HIX is the humidification index.	HIX denotes the extent of humidification. A larger HIX value indicates a higher degree of humification or a larger abundance of humic substances.
BIX	$\text{BIX} = \frac{\text{Fluorescence intensity at 380 nm}}{\text{Fluorescence intensity at 430 nm}}$ BIX is the biological index.	BIX denotes the microbial-derived contribution to fluorophores. A larger BIX value indicates a larger microbial-derived contribution.

Table S11. Φ_{RI} and $[RI]_{ss}$ of bulk and MW- fractionated COM samples from culturable bacteria in $PM_{2.5}$ (DOC = 5 mg C/L).

	**Seasons**	**C0 (Bulk)**	**C1 (<1 kDa)**	**C2 (1–3 kDa)**	**C3 (3–10 kDa)**	**C4 (10–30 kDa)**	**C5 (30–50 kDa)**	**C6 (>50 kDa)**
$\Phi_{\cdot OH}$ (× 10^{-2} %)	Summer	0.61 ± 0.07	1.33 ± 0.14	0.86 ± 0.05	0.77 ± 0.05	0.58 ± 0.04	0.50 ± 0.05	0.31 ± 0.06
	Winter	0.60 ± 0.05	1.23 ± 0.19	0.91 ± 0.06	0.78 ± 0.09	0.59 ± 0.05	0.52 ± 0.05	0.33 ± 0.04
$\Phi_{^1O_2^*}$ (%)	Summer	4.67 ± 0.56	10.98 ± 0.70	8.58 ± 0.47	7.27 ± 0.44	5.79 ± 0.76	4.65 ± 0.74	3.16 ± 0.69
	Winter	4.87 ± 0.67	10.57 ± 0.98	8.86 ± 0.86	6.89 ± 0.40	5.21 ± 0.55	4.42 ± 0.70	2.92 ± 0.59
$\Phi_{^3C^*_{TMP}}$ (%)	Summer	2.51 ± 0.11	5.69 ± 0.72	4.38 ± 0.23	3.33 ± 0.39	2.65 ± 0.41	1.65 ± 0.43	1.43 ± 0.26
	Winter	2.32 ± 0.14	5.45 ± 0.51	4.39 ± 0.23	3.58 ± 0.24	2.38 ± 0.27	1.48 ± 0.25	1.42 ± 0.24
$\Phi_{^3C^*_{HDO}}$ (%)	Summer	0.62 ± 0.08	1.17 ± 0.11	0.93 ± 0.06	0.80 ± 0.08	0.72 ± 0.02	0.50 ± 0.02	0.35 ± 0.02
	Winter	0.62 ± 0.08	1.24 ± 0.14	0.92 ± 0.05	0.79 ± 0.12	0.75 ± 0.06	0.48 ± 0.04	0.35 ± 0.01
$[\cdot OH]_{ss}$ (× 10^{-17} M)	Summer	1.72 ± 0.05	4.09 ± 0.21	3.51 ± 0.14	2.86 ± 0.23	2.41 ± 0.12	1.72 ± 0.15	1.26 ± 0.11
	Winter	1.79 ± 0.14	4.18 ± 0.17	3.40 ± 0.13	2.87 ± 0.10	2.31 ± 0.15	1.87 ± 0.12	1.16 ± 0.15
$[^1O_2^*]_{ss}$(× 10^{-14} M)	Summer	47.25 ± 3.49	75.22 ± 2.71	65.30 ± 4.82	56.34 ± 6.12	55.45 ± 5.35	44.87 ± 3.81	37.58 ± 2.78
	Winter	46.39 ± 6.24	77.69 ± 2.65	66.52 ± 4.84	58.26 ± 6.23	60.11 ± 5.13	45.73 ± 2.97	34.93 ± 1.50
$[^3C^*_{TMP}]_{ss}$ (× 10^{-14} M)	Summer	0.75 ± 0.03	1.86 ± 0.07	1.53 ± 0.12	0.91 ± 0.23	0.77 ± 0.08	0.72 ± 0.06	0.63 ± 0.10
	Winter	0.81 ± 0.09	1.84 ± 0.10	1.56 ± 0.11	0.88 ± 0.11	0.73 ± 0.08	0.70 ± 0.07	0.63 ± 0.05
$[^3C^*_{HDO}]_{ss}$ (× 10^{-14} M)	Summer	6.94 ± 0.33	12.13 ± 0.98	9.30 ± 0.49	6.88 ± 0.52	6.85 ± 1.03	5.63 ± 0.69	5.46 ± 1.05
	Winter	6.95 ± 0.74	12.41 ± 0.97	9.58 ± 1.05	7.65 ± 0.75	6.97 ± 0.62	5.90 ± 0.71	5.23 ± 0.64

Table S12. Φ_{RI} and $[RI]_{ss}$ of bulk and MW- fractionated EPS samples from culturable bacteria in $PM_{2.5}$ (DOC = 5 mg C/L).

EPS	Seasons	E0 (Bulk)	E1 (<1 kDa)	E2 (1–3 kDa)	E3 (3–10 kDa)	E4 (10–30 kDa)	E5 (30–50 kDa)	E6 (>50 kDa)
$\Phi_{\cdot OH}$ (× 10^{-2} %)	Summer	0.47 ± 0.06	0.76 ± 0.12	0.61 ± 0.05	0.50 ± 0.06	0.44 ± 0.07	0.37 ± 0.04	0.22 ± 0.03
	Winter	0.45 ± 0.06	0.83 ± 0.13	0.63 ± 0.06	0.51 ± 0.05	0.48 ± 0.06	0.37 ± 0.04	0.22 ± 0.04
$\Phi_{^1O_2^*}$ (%)	Summer	4.12 ± 0.73	8.89 ± 0.88	7.05 ± 0.55	5.37 ± 0.50	3.89 ± 0.50	3.25 ± 0.36	2.06 ± 0.65
	Winter	4.07 ± 0.79	8.93 ± 0.70	7.09 ± 0.53	5.42 ± 0.56	3.79 ± 0.48	3.04 ± 0.33	1.97 ± 0.78
$\Phi_{^3C^*_{TMP}}$ (%)	Summer	1.73 ± 0.26	3.87 ± 0.27	3.19 ± 0.40	2.82 ± 0.37	2.43 ± 0.53	1.44 ± 0.20	1.53 ± 0.23
	Winter	1.85 ± 0.28	3.85 ± 0.36	3.07 ± 0.34	2.90 ± 0.39	2.49 ± 0.28	1.36 ± 0.27	1.51 ± 0.20
$\Phi_{^3C^*_{HDO}}$ (%)	Summer	0.37 ± 0.03	0.60 ± 0.02	0.52 ± 0.04	0.45 ± 0.03	0.37 ± 0.04	0.31 ± 0.04	0.26 ± 0.02
	Winter	0.37 ± 0.02	0.60 ± 0.03	0.55 ± 0.02	0.46 ± 0.03	0.39 ± 0.03	0.31 ± 0.05	0.25 ± 0.01
$[\cdot OH]_{ss}$ (× 10^{-17} M)	Summer	1.00 ± 0.18	2.57 ± 0.34	1.84 ± 0.19	1.58 ± 0.23	1.31 ± 0.24	0.98 ± 0.09	0.77 ± 0.08
	Winter	0.97 ± 0.09	2.74 ± 0.17	1.95 ± 0.23	1.69 ± 0.17	1.26 ± 0.04	1.00 ± 0.12	0.78 ± 0.06
$[^1O_2^*]_{ss}$(× 10^{-14} M)	Summer	31.03 ± 3.39	48.40 ± 3.19	44.23 ± 2.52	37.12 ± 2.89	32.93 ± 3.30	26.31 ± 1.69	19.11 ± 1.69
	Winter	31.73 ± 3.67	47.28 ± 3.41	44.63 ± 2.20	37.15 ± 3.71	32.21 ± 3.46	26.21 ± 1.70	19.00 ± 1.92
$[^3C^*_{TMP}]_{ss}$ (× 10^{-14} M)	Summer	0.70 ± 0.06	1.50 ± 0.10	1.26 ± 0.14	1.20 ± 0.08	0.90 ± 0.16	0.76 ± 0.06	0.41 ± 0.08
	Winter	0.68 ± 0.05	1.50 ± 0.13	1.21 ± 0.10	1.24 ± 0.07	0.82 ± 0.09	0.77 ± 0.08	0.43 ± 0.10
$[^3C^*_{HDO}]_{ss}$ (× 10^{-14} M)	Summer	4.45 ± 0.39	7.88 ± 0.57	6.26 ± 0.32	5.47 ± 0.31	4.31 ± 0.27	4.00 ± 0.48	3.26 ± 0.20
	Winter	4.49 ± 0.57	7.71 ± 0.57	6.40 ± 0.35	5.51 ± 0.36	4.25 ± 0.21	3.79 ± 0.50	3.25 ± 0.21

Table S13. Φ_{RI} and $[RI]_{ss}$ of bulk and MW- fractionated COM samples extracted from pure laboratory cultures (DOC = 5 mg C/L).

	**Strains**	**C0 (Bulk)**	**C1 (<1 kDa)**	**C2 (1–3 kDa)**	**C3 (3–10 kDa)**	**C4 (10–30 kDa)**	**C5 (30–50 kDa)**	**C6 (>50 kDa)**
$\Phi_{\cdot OH}$ (× 10^{-2} %)	*B. subtilis*	0.57 ± 0.02	1.35 ± 0.03	0.89 ± 0.01	0.80 ± 0.01	0.60 ± 0.01	0.49 ± 0.01	0.37 ± 0.01
	P. putida	0.62 ± 0.01	1.27 ± 0.01	0.91 ± 0.01	0.74 ± 0.01	0.56 ± 0.01	0.53 ± 0.01	0.31 ± 0.01
	E. hormaechei B0910	0.67 ± 0.01	1.34 ± 0.02	0.85 ± 0.02	0.76 ± 0.01	0.57 ± 0.01	0.51 ± 0.02	0.27 ± 0.01
	E. hormaechei pf0910	0.59 ± 0.02	1.21 ± 0.01	0.93 ± 0.02	0.83 ± 0.02	0.63 ± 0.01	0.53 ± 0.02	0.37 ± 0.01
$\Phi_{^1O_2^*}$ (%)	*B. subtilis*	4.95 ± 0.15	10.97 ± 0.21	8.63 ± 0.01	7.24 ± 0.04	5.87 ± 0.23	4.07 ± 0.02	2.97 ± 0.20
	P. putida	5.48 ± 0.02	10.49 ± 0.08	9.65 ± 0.01	6.75 ± 0.10	5.18 ± 0.06	4.59 ± 0.11	2.47 ± 0.07
	E. hormaechei B0910	4.44 ± 0.04	11.12 ± 0.06	8.64 ± 0.15	7.40 ± 0.13	5.78 ± 0.04	5.29 ± 0.07	3.39 ± 0.03
	E. hormaechei pf0910	4.32 ± 0.01	10.78 ± 0.28	8.17 ± 0.02	7.12 ± 0.05	5.30 ± 0.16	4.30 ± 0.18	3.40 ± 0.03
$\Phi_{^3C^*_{TMP}}$ (%)	*B. subtilis*	2.53 ± 0.04	5.29 ± 0.12	4.33 ± 0.07	3.37 ± 0.07	2.62 ± 0.13	1.81 ± 0.12	1.45 ± 0.07
	P. putida	2.34 ± 0.02	5.81 ± 0.01	4.34 ± 0.07	3.58 ± 0.07	2.32 ± 0.05	1.56 ± 0.07	1.51 ± 0.07
	E. hormaechei B0910	2.52 ± 0.01	6.16 ± 0.11	4.49 ± 0.01	3.33 ± 0.10	2.73 ± 0.01	1.52 ± 0.04	1.44 ± 0.04
	E. hormaechei pf0910	2.34 ± 0.04	5.17 ± 0.11	4.50 ± 0.01	3.64 ± 0.03	2.48 ± 0.06	1.43 ± 0.02	1.35 ± 0.02
$\Phi_{^3C^*_{HDO}}$ (%)	*B. subtilis*	0.64 ± 0.01	1.10 ± 0.02	0.92 ± 0.02	0.74 ± 0.01	0.71 ± 0.01	0.52 ± 0.01	0.37 ± 0.01
	P. putida	0.69 ± 0.01	1.20 ± 0.04	0.92 ± 0.02	0.74 ± 0.03	0.77 ± 0.02	0.49 ± 0.02	0.36 ± 0.01
	E. hormaechei B0910	0.63 ± 0.03	1.26 ± 0.01	0.96 ± 0.01	0.88 ± 0.01	0.74 ± 0.01	0.50 ± 0.01	0.34 ± 0.01
	E. hormaechei pf0910	0.58 ± 0.02	1.31 ± 0.01	0.94 ± 0.01	0.86 ± 0.02	0.75 ± 0.01	0.50 ± 0.01	0.34 ± 0.01
$[\cdot OH]_{ss}$ (× 10^{-17} M)	*B. subtilis*	1.71 ± 0.02	4.27 ± 0.04	3.63 ± 0.18	2.98 ± 0.03	2.45 ± 0.04	1.83 ± 0.01	1.19 ± 0.01
	P. putida	1.77 ± 0.03	4.32 ± 0.03	3.49 ± 0.03	2.84 ± 0.02	2.36 ± 0.02	1.89 ± 0.01	1.05 ± 0.01
	E. hormaechei B0910	1.75 ± 0.02	3.98 ± 0.01	3.44 ± 0.02	2.78 ± 0.06	2.40 ± 0.02	1.63 ± 0.03	1.35 ± 0.02
	E. hormaechei pf0910	1.85 ± 0.04	4.09 ± 0.02	3.35 ± 0.01	2.94 ± 0.03	2.31 ± 0.05	1.89 ± 0.04	1.31 ± 0.01
$[^1O_2^*]_{ss}$(× 10^{-14} M)	*B. subtilis*	48.78 ± 0.68	76.48 ± 0.62	65.48 ± 1.44	59.53 ± 1.07	53.65 ± 1.43	43.90 ± 0.69	39.17 ± 0.55
	P. putida	49.93 ± 0.93	80.22 ± 0.25	64.69 ± 1.20	59.68 ± 1.07	56.81 ± 0.70	45.51 ± 0.33	35.24 ± 0.07
	E. hormaechei B0910	46.25 ± 0.67	74.81 ± 0.45	65.84 ± 0.16	56.78 ± 1.13	57.88 ± 0.07	46.34 ± 0.82	36.42 ± 0.42
	E. hormaechei pf0910	43.36 ± 1.17	76.01 ± 0.21	69.09 ± 0.35	57.49 ± 1.49	52.08 ± 0.56	46.46 ± 0.82	35.01 ± 0.45
$[^3C^*_{TMP}]_{ss}$ (× 10^{-14} M)	*B. subtilis*	0.76 ± 0.01	1.94 ± 0.01	1.63 ± 0.03	1.00 ± 0.06	0.84 ± 0.01	0.77 ± 0.01	0.65 ± 0.03
	P. putida	0.84 ± 0.02	1.94 ± 0.01	1.63 ± 0.03	0.93 ± 0.01	0.77 ± 0.03	0.73 ± 0.02	0.66 ± 0.01
	E. hormaechei B0910	0.75 ± 0.01	1.82 ± 0.01	1.46 ± 0.01	0.84 ± 0.03	0.71 ± 0.01	0.68 ± 0.01	0.63 ± 0.02
	E. hormaechei pf0910	0.80 ± 0.03	1.77 ± 0.02	1.52 ± 0.02	0.84 ± 0.03	0.71 ± 0.01	0.68 ± 0.01	0.63 ± 0.02
$[^3C^*_{HDO}]_{ss}$ (× 10^{-14} M)	*B. subtilis*	6.88 ± 0.08	12.37 ± 0.14	9.77 ± 0.04	6.55 ± 0.05	6.56 ± 0.24	5.19 ± 0.04	4.79 ± 0.10
	P. putida	6.39 ± 0.05	12.90 ± 0.02	9.30 ± 0.16	8.08 ± 0.18	6.78 ± 0.04	5.71 ± 0.16	4.80 ± 0.10
	E. hormaechei B0910	7.07 ± 0.06	12.03 ± 0.26	8.95 ± 0.01	7.30 ± 0.07	7.24 ± 0.17	6.14 ± 0.13	6.21 ± 0.18
	E. hormaechei pf0910	7.59 ± 0.08	12.06 ± 0.26	9.98 ± 0.25	7.33 ± 0.07	7.26 ± 0.17	6.16 ± 0.13	5.72 ± 0.05

Table S14. Φ_{RI} and $[RI]_{ss}$ of bulk and MW- fractionated EPS samples extracted from pure laboratory cultures (DOC = 5 mg C/L).

	**Strains**	**E0 (Bulk)**	**E1 (<1 kDa)**	**E2 (1–3 kDa)**	**E3 (3–10 kDa)**	**E4 (10–30 kDa)**	**E5 (30–50 kDa)**	**E6 (>50 kDa)**
$\Phi_{\cdot OH}$ (× 10^{-2} %)	*B. subtilis*	0.51 ± 0.02	0.71 ± 0.01	0.61 ± 0.02	0.53 ± 0.01	0.40 ± 0.01	0.36 ± 0.01	0.21 ± 0.03
	P. putida	0.41 ± 0.01	0.95 ± 0.03	0.60 ± 0.03	0.51 ± 0.02	0.51 ± 0.01	0.34 ± 0.01	0.24 ± 0.02
	E. hormaechei B0910	0.45 ± 0.06	0.71 ± 0.01	0.69 ± 0.05	0.45 ± 0.05	0.41 ± 0.05	0.37 ± 0.03	0.22 ± 0.04
	E. hormaechei pf0910	0.52 ± 0.01	0.71 ± 0.06	0.58 ± 0.06	0.56 ± 0.05	0.49 ± 0.01	0.41 ± 0.01	0.22 ± 0.01
$\Phi_{^1O_2^*}$ (%)	*B. subtilis*	4.26 ± 0.27	8.58 ± 0.32	6.54 ± 0.53	4.99 ± 0.14	4.39 ± 0.09	3.39 ± 0.24	1.70 ± 0.13
	P. putida	3.97 ± 0.13	9.05 ± 0.05	7.21 ± 0.13	5.07 ± 0.11	3.58 ± 0.14	3.15 ± 0.11	1.32 ± 0.02
	E. hormaechei B0910	4.79 ± 0.09	8.83 ± 0.13	7.31 ± 0.84	5.51 ± 0.08	3.43 ± 0.39	3.26 ± 0.76	2.50 ± 0.06
	E. hormaechei pf0910	3.58 ± 0.20	9.33 ± 0.59	7.32 ± 0.06	6.03 ± 0.15	4.27 ± 0.05	3.27 ± 0.04	2.80 ± 0.01
$\Phi_{^3C^*_{TMP}}$ (%)	*B. subtilis*	1.69 ± 0.03	4.04 ± 0.06	3.25 ± 0.09	2.98 ± 0.02	2.73 ± 0.21	1.57 ± 0.06	1.62 ± 0.05
	P. putida	1.79 ± 0.03	3.59 ± 0.31	3.10 ± 0.04	3.21 ± 0.02	2.60 ± 0.06	1.19 ± 0.08	1.57 ± 0.10
	E. hormaechei B0910	1.70 ± 0.09	4.05 ± 0.43	2.92 ± 0.36	2.29 ± 0.14	2.28 ± 0.71	1.47 ± 0.09	1.33 ± 0.18
	E. hormaechei pf0910	1.78 ± 0.21	3.91 ± 0.17	3.58 ± 0.17	2.86 ± 0.08	2.19 ± 0.02	1.58 ± 0.04	1.63 ± 0.04
$\Phi_{^3C^*_{HDO}}$ (%)	*B. subtilis*	0.34 ± 0.01	0.60 ± 0.01	0.50 ± 0.01	0.43 ± 0.01	0.34 ± 0.01	0.31 ± 0.01	0.27 ± 0.01
	P. putida	0.36 ± 0.02	0.59 ± 0.01	0.57 ± 0.01	0.47 ± 0.01	0.38 ± 0.01	0.33 ± 0.01	0.26 ± 0.01
	E. hormaechei B0910	0.39 ± 0.01	0.60 ± 0.03	0.50 ± 0.05	0.45 ± 0.01	0.36 ± 0.05	0.27 ± 0.04	0.26 ± 0.01
	E. hormaechei pf0910	0.40 ± 0.01	0.63 ± 0.03	0.54 ± 0.04	0.46 ± 0.05	0.42 ± 0.01	0.37 ± 0.01	0.29 ± 0.01
$[\cdot OH]_{ss}$ (× 10^{-17} M)	*B. subtilis*	1.20 ± 0.09	2.35 ± 0.01	1.75 ± 0.07	1.63 ± 0.02	1.64 ± 0.01	1.01 ± 0.01	0.85 ± 0.04
	P. putida	0.97 ± 0.07	2.76 ± 0.10	2.16 ± 0.01	1.79 ± 0.02	1.30 ± 0.05	1.03 ± 0.02	0.79 ± 0.02
	E. hormaechei B0910	0.84 ± 0.09	2.96 ± 0.01	1.84 ± 0.01	1.60 ± 0.01	1.21 ± 0.01	0.93 ± 0.08	0.72 ± 0.01
	E. hormaechei pf0910	1.02 ± 0.12	2.29 ± 0.41	1.68 ± 0.01	1.38 ± 0.40	1.15 ± 0.04	1.00 ± 0.03	0.76 ± 0.03
$[^1O_2^*]_{ss}$(× 10^{-14} M)	*B. subtilis*	33.13 ± 0.52	47.23 ± 0.19	41.47 ± 0.11	35.49 ± 0.34	34.12 ± 0.39	25.21 ± 0.22	17.95 ± 0.57
	P. putida	28.89 ± 0.38	49.19 ± 1.50	43.02 ± 1.32	35.03 ± 0.01	31.15 ± 0.42	25.24 ± 0.09	19.48 ± 0.12
	E. hormaechei B0910	31.30 ± 1.99	47.48 ± 2.87	46.99 ± 1.28	37.64 ± 1.54	31.56 ± 0.52	28.87 ± 0.76	20.51 ± 0.83
	E. hormaechei pf0910	31.48 ± 3.21	50.77 ± 2.35	46.42 ± 0.76	41.15 ± 2.47	35.65 ± 1.22	26.51 ± 0.73	18.95 ± 0.68
$[^3C^*_{TMP}]_{ss}$ (× 10^{-14} M)	*B. subtilis*	0.68 ± 0.03	1.49 ± 0.01	1.19 ± 0.02	1.10 ± 0.03	1.04 ± 0.04	0.74 ± 0.01	0.35 ± 0.01
	P. putida	0.74 ± 0.03	1.59 ± 0.08	1.30 ± 0.04	1.25 ± 0.02	0.85 ± 0.04	0.79 ± 0.01	0.50 ± 0.04
	E. hormaechei B0910	0.70 ± 0.08	1.59 ± 0.09	1.28 ± 0.04	1.26 ± 0.09	0.96 ± 0.15	0.75 ± 0.01	0.40 ± 0.09
	E. hormaechei pf0910	0.71 ± 0.11	1.39 ± 0.08	1.30 ± 0.16	1.22 ± 0.04	0.79 ± 0.02	0.80 ± 0.01	0.44 ± 0.02
$[^3C^*_{HDO}]_{ss}$ (× 10^{-14} M)	*B. subtilis*	4.38 ± 0.07	8.05 ± 0.02	6.07 ± 0.31	5.70 ± 0.15	4.42 ± 0.07	4.11 ± 0.12	3.15 ± 0.05
	P. putida	4.26 ± 0.13	8.16 ± 0.15	6.64 ± 0.05	5.61 ± 0.19	4.17 ± 0.08	3.76 ± 0.06	3.26 ± 0.03
	E. hormaechei B0910	4.39 ± 0.12	7.80 ± 0.11	6.29 ± 0.13	5.54 ± 0.42	4.56 ± 0.08	4.46 ± 0.07	3.28 ± 0.40
	E. hormaechei pf0910	4.88 ± 0.08	7.68 ± 0.19	6.21 ± 0.29	5.17 ± 0.01	4.19 ± 0.02	3.79 ± 0.14	3.43 ± 0.03

Table S15. Statistics of MLR models for the oxidants.

	Coefficient	Estimate	Standard Error	VIF	p value
$\Phi_{\cdot OH}$	β0 (Intercept)	-0.3901	0.05919		<0.0001
	β1 (E_2/E_3)	0.5025	0.02943	1.000	<0.0001
	β0 (Intercept)	-2.843	0.4440		<0.0001
$\Phi_{^1O_2^*}$	β1 (E_2/E_3)	0.6073	0.09096	2.268	<0.0001
	β2 (FIX)	3.395	0.3062	2.268	<0.0001
	β0 (Intercept)	-1.318	0.2674		<0.0001
$\Phi_{^3C_{TMP}^*}$	β1 (E_2/E_3)	0.2827	0.05477	2.268	<0.0001
	β2 (FIX)	1.633	0.1844	2.268	<0.0001
$\Phi_{^3C_{HDO}^*}$	β0 (Intercept)	-0.3642	0.06586		<0.0001
	β1 (E_2/E_3)	0.007675	0.01349	2.268	0.576
	β3 (FIX)	0.4655	0.04541	2.268	<0.0001
	β0 (Intercept)	-2.015	0.2863		<0.0001
$[\cdot OH]_{ss}$	β1 (E_2/E_3)	0.4496	0.08669	2.828	<0.0001
	β2 (FIX)	1.504	0.1320	2.218	<0.0001
	β3 ($SUVA_{254}$)	0.2035	0.06783	1.844	<0.0001
$[^1O_2^*]_{ss}$	β0 (Intercept)	-16.41	3.411		<0.0001
	β1 (E_2/E_3)	-1.884	1.347	2.193	0.1646
$[^3C_{TMP}^*]_{ss}$	β2 (FIX)	32.60	2.315	2.193	<0.0001
	β0 (Intercept)	-0.2453	0.08858		0.0066
	β1 (E_2/E_3)	0.2341	0.03497	2.193	<0.0001
$[^3C_{HDO}^*]_{ss}$	β2 (FIX)	0.4518	0.06011	2.193	<0.0001
	β0 (Intercept)	-1.893	0.5173		0.0004
	β1 (E_2/E_3)	0.5710	0.2042	2.193	0.0061

References

1. Caporaso, J. G.; Kuczynski, J.; Stombaugh, J.; Bittinger, K.; Bushman, F. D.; Costello, E. K.; Fierer, N.; Peña, A. G.; Goodrich, J. K.; Gordon, J. I.; Huttley, G. A.; Kelley, S. T.; Knights, D.; Koenig, J. E.; Ley, R. E.; Lozupone, C. A.; McDonald, D.; Muegge, B. D.; Pirrung, M.; Reeder, J.; Sevinsky, J. R.; Turnbaugh, P. J.; Walters, W. A.; Widmann, J.; Yatsunenko, T.; Zaneveld, J.; Knight, R., QIIME allows analysis of high-throughput community sequencing data. *Nature Methods* **2010,** *7*, (5), 335-336.

2. Caporaso, J. G.; Lauber, C. L.; Walters, W. A.; Berg-Lyons, D.; Huntley, J.; Fierer, N.; Owens, S. M.; Betley, J.; Fraser, L.; Bauer, M., Ultra-high-throughput microbial community analysis on the Illumina HiSeq and MiSeq platforms. *The ISME journal* **2012,** *6*, (8), 1621-1624.

3. Yu, J.; Tang, S. N.; Lee, P. K. H., Microbial Communities in Full-Scale Wastewater Treatment Systems Exhibit Deterministic Assembly Processes and Functional Dependency over Time. *Environmental Science & Technology* **2021,** *55*, (8), 5312-5323.

4. Zhou, Y.; Leung, M. H. Y.; Tong, X.; Lee, J. Y. Y.; Lee, P. K. H., City-Scale Meta-Analysis of Indoor Airborne Microbiota Reveals that Taxonomic and Functional Compositions Vary with Building Types. *Environmental Science & Technology* **2021,** *55*, (22), 15051-15062.

5. Callahan, B. J.; McMurdie, P. J.; Rosen, M. J.; Han, A. W.; Johnson, A. J. A.; Holmes, S. P., DADA2: High-resolution sample inference from Illumina amplicon data. *Nature Methods* **2016,** *13*, (7), 581-583.

6. Quast, C.; Pruesse, E.; Yilmaz, P.; Gerken, J.; Schweer, T.; Yarza, P.; Peplies, J.; Glöckner, F. O., The SILVA ribosomal RNA gene database project: improved data processing and web-based tools. *Nucleic Acids Res* **2013,** *41*, (Database issue), D590-6.

7. Li, Y.; Zhang, K.; Apell, J.; Ruan, Y.; Huang, X.; Nah, T., Photoproduction of reactive intermediates from dissolved organic matter in coastal seawater around an urban metropolis in South China: Characterization and predictive modeling. *Science of The Total Environment* **2024**, 170998.

8. Mielnik, L.; Kowalczuk, P., Optical characteristic of humic acids from lake sediments by excitation-emission matrix fluorescence with PARAFAC model. *Journal of Soils and Sediments* **2018,** *18*, (8), 2851-2862.

9. Sierra, M. M. D.; Giovanela, M.; Parlanti, E.; Soriano-Sierra, E. J., Fluorescence fingerprint of fulvic and humic acids from varied origins as viewed by single-scan and excitation/emission matrix techniques. *Chemosphere* **2005,** *58*, (6), 715-733.

10. McKnight, D. M.; Boyer, E. W.; Westerhoff, P. K.; Doran, P. T.; Kulbe, T.; Andersen, D. T., Spectrofluorometric characterization of dissolved organic matter for indication of precursor organic material and aromaticity. *Limnology and Oceanography* **2001,** *46*, (1), 38-48.

11. Pucher, M.; Wünsch, U.; Weigelhofer, G.; Murphy, K.; Hein, T.; Graeber, D., staRdom: versatile software for analyzing spectroscopic data of dissolved organic matter in R. *Water* **2019,** *11*, (11), 2366.

12. Yue, S.; Ren, L.; Song, T.; Li, L.; Xie, Q.; Li, W.; Kang, M.; Zhao, W.; Wei, L.; Ren, H., Abundance and diurnal trends of fluorescent bioaerosols in the troposphere over Mt. Tai, China, in spring. *Journal of Geophysical Research: Atmospheres* **2019,** *124*, (7), 4158-4173.

13. Chen, Q.; Miyazaki, Y.; Kawamura, K.; Matsumoto, K.; Coburn, S.; Volkamer, R.; Iwamoto, Y.; Kagami, S.; Deng, Y.; Ogawa, S., Characterization of chromophoric water-soluble organic matter in urban, forest, and marine aerosols by HR-ToF-AMS analysis and excitation–emission matrix spectroscopy. *Environmental Science & Technology* **2016,** *50*, (19), 10351-10360.

14. Pöhlker, C.; Huffman, J.; Pöschl, U., Autofluorescence of atmospheric bioaerosols–fluorescent biomolecules and potential interferences. *Atmospheric Measurement Techniques* **2012,** *5*, (1), 37-71.

15. Wu, G.; Fu, P.; Ram, K.; Song, J.; Chen, Q.; Kawamura, K.; Wan, X.; Kang, S.; Wang, X.; Laskin, A.; Cong, Z., Fluorescence characteristics of water-soluble organic carbon in atmospheric aerosol☆. *Environmental Pollution* **2021,** *268*, 115906.

16. Ossola, R.; Jönsson, O. M.; Moor, K.; McNeill, K., Singlet Oxygen Quantum Yields in Environmental Waters. *Chemical Reviews* **2021,** *121*, (7), 4100-4146.

17. Vione, D.; Falletti, G.; Maurino, V.; Minero, C.; Pelizzetti, E.; Malandrino, M.; Ajassa, R.; Olariu, R.-I.; Arsene, C., Sources and Sinks of Hydroxyl Radicals upon Irradiation of Natural Water Samples. *Environmental Science & Technology* **2006,** *40*, (12), 3775-3781.

18. Buxton, G. V.; Greenstock, C. L.; Helman, W. P.; Ross, A. B., Critical Review of rate constants for reactions of hydrated electrons, hydrogen atoms and hydroxyl radicals (·OH/·O− in Aqueous Solution. *Journal of Physical and Chemical Reference Data* **1988,** *17*, (2), 513-886.

19. Anastasio, C.; McGregor, K. G., Chemistry of fog waters in California's Central Valley: 1. In situ photoformation of hydroxyl radical and singlet molecular oxygen. *Atmospheric Environment* **2001,** *35*, (6), 1079-1089.

20. Zhou, X.; Mopper, K., Determination of photochemically produced hydroxyl radicals in seawater and freshwater. *Marine Chemistry* **1990,** *30*, 71-88.

21. Kaur, R.; Anastasio, C., Light absorption and the photoformation of hydroxyl radical and singlet oxygen in fog waters. *Atmospheric Environment* **2017,** *164*, 387-397.

22. Wan, D.; Sharma, V. K.; Liu, L.; Zuo, Y.; Chen, Y., Mechanistic insight into the effect of metal ions on photogeneration of reactive species from dissolved organic matter. *Environmental science & technology* **2019,** *53*, (10), 5778-5786.

23. Bregnhøj, M.; Westberg, M.; Jensen, F.; Ogilby, P. R., Solvent-dependent singlet oxygen lifetimes: temperature effects implicate tunneling and charge-transfer interactions. *Physical Chemistry Chemical Physics* **2016,** *18*, (33), 22946-22961.

24. Housari, F. a.; Vione, D.; Chiron, S.; Barbati, S., Reactive photoinduced species in estuarine waters. Characterization of hydroxyl radical, singlet oxygen and dissolved organic matter triplet state in natural oxidation processes. *Photochemical & Photobiological Sciences* **2010,** *9*, (1), 78-86.

25. Canonica, S.; Hellrung, B.; Wirz, J., Oxidation of Phenols by Triplet Aromatic Ketones in Aqueous Solution. *The Journal of Physical Chemistry A* **2000,** *104*, (6), 1226-1232.

26. Wasswa, J.; Driscoll, C. T.; Zeng, T., Photochemical Characterization of Surface Waters from Lakes in the Adirondack Region of New York. *Environmental Science & Technology* **2020,** *54*, (17), 10654-10667.

27. Erickson, P. R.; Moor, K. J.; Werner, J. J.; Latch, D. E.; Arnold, W. A.; McNeill, K., Singlet Oxygen Phosphorescence as a Probe for Triplet-State Dissolved Organic Matter Reactivity. *Environmental Science & Technology* **2018,** *52*, (16), 9170-9178.

28. McCabe, A. J.; Arnold, W. A., Reactivity of Triplet Excited States of Dissolved Natural Organic Matter in Stormflow from Mixed-Use Watersheds. *Environmental Science & Technology* **2017,** *51*, (17), 9718-9728.

29. Canonica, S.; Laubscher, H.-U., Inhibitory effect of dissolved organic matter on triplet-induced oxidation of aquatic contaminants. *Photochemical & Photobiological Sciences* **2008,** *7*, (5), 547-551.

30. Zhou, H.; Yan, S.; Ma, J.; Lian, L.; Song, W., Development of Novel Chemical Probes for Examining Triplet Natural Organic Matter under Solar Illumination. *Environmental Science & Technology* **2017,** *51*, (19), 11066-11074.

31. Trygg, J.; Wold, S., Orthogonal projections to latent structures (O-PLS). *Journal of Chemometrics* **2002,** *16*, (3), 119-128.

32. Galindo-Prieto, B.; Eriksson, L.; Trygg, J., Variable influence on projection (VIP) for orthogonal projections to latent structures (OPLS). *Journal of Chemometrics* **2014,** *28*, (8), 623-632.

33. Wasswa, J.; Driscoll, C. T.; Zeng, T., Photochemical Characterization of Surface Waters from Lakes in the Adirondack Region of New York. *Environ Sci Technol* **2020,** *54*, (17), 10654-10667.

34. Palansooriya, K. N.; Li, J.; Dissanayake, P. D.; Suvarna, M.; Li, L.; Yuan, X.; Sarkar, B.; Tsang, D. C.; Rinklebe, J. r.; Wang, X., Prediction of soil heavy metal immobilization by biochar using machine learning. *Environmental science & technology* **2022,** *56*, (7), 4187-4198.

35. Yuan, X.; Suvarna, M.; Low, S.; Dissanayake, P. D.; Lee, K. B.; Li, J.; Wang, X.; Ok, Y. S., Applied machine learning for prediction of CO2 adsorption on biomass waste-derived porous carbons. *Environmental Science & Technology* **2021,** *55*, (17), 11925-11936.

36. Molnar, C., *Interpretable machine learning*. Lulu. com: 2020.

37. Lundberg, S. M.; Lee, S.-I., A unified approach to interpreting model predictions. *Advances in neural information processing systems* **2017,** *30*.

38. Shapley, L. S., 17. A Value for n-Person Games. In *Contributions to the Theory of Games, Volume II*, Harold William, K.; Albert William, T., Eds. Princeton University Press: Princeton, 1953; pp 307-318.

39. Liao, Z.; Lu, J.; Xie, K.; Wang, Y.; Yuan, Y., Prediction of photochemical properties of dissolved organic matter using machine learning. *Environmental Science & Technology* **2023,** *57*, (46), 17971-17980.

40. Pöhlker, C.; Huffman, J. A.; Pöschl, U., Autofluorescence of atmospheric bioaerosols – fluorescent biomolecules and potential interferences. *Atmospheric Measurement Techniques* **2012,** *5*, (1), 37-71.

41. Chen, Q.; Ikemori, F.; Mochida, M., Light absorption and excitation–emission fluorescence of urban organic aerosol components and their relationship to chemical structure. *Environmental Science & Technology* **2016,** *50*, (20), 10859-10868.
